# Advances in TENGs for Marine Energy Harvesting and In Situ Electrochemistry

**DOI:** 10.1007/s40820-024-01640-w

**Published:** 2025-01-31

**Authors:** Chuguo Zhang, Yijun Hao, Xiangqian Lu, Wei Su, Hongke Zhang, Zhong Lin Wang, Xiuhan Li

**Affiliations:** 1 School of Electronic and Information Engineering, Beijing Jiaotong University, Beijing, 100044 People’s Republic of China; 2 CAS Center for Excellence in Nanoscience, Beijing Key Laboratory of Micro-Nano Energy and Sensor, Beijing, 100190 People’s Republic of China; 3 Beijing Institute of Nanoenergy and Nanosystems, Chinese Academy of Sciences, Beijing, 101400 People’s Republic of China

**Keywords:** Triboelectric nanogenerator, Marine energy, Power take-off, Self-powered, Electrochemistry

## Abstract

The large-scale use of ample marine energy will be one of the most important ways for human to achieve sustainable development through carbon neutral development plans. As a burgeoning technological method for electromechanical conversion, triboelectric nanogenerator (TENG) has significant advantages in marine energy for its low weight, cost-effectiveness, and high efficiency in low-frequency range. It can realize the efficient and economical harvesting of low-frequency blue energy by constructing the floating marine energy harvesting TENG. This paper firstly introduces the power transfer process and structural composition of TENG for marine energy harvesting in detail. In addition, the latest research works of TENG on marine energy harvesting in basic research and structural design are systematically reviewed by category. Finally, the advanced research progress in the power take-off types and engineering study of TENG with the marine energy are comprehensively generalized. Importantly, the challenges and problems faced by TENG in marine energy and in situ electrochemical application are summarized and the corresponding prospects and suggestions are proposed for the subsequent development direction and prospects to look forward to promoting the commercialization process of this field.

## Highlights

- The basic information of triboelectric nanogenerator (TENG), the power conversion process, and key points of the marine energy harvesting TENGs was introduced in detail.
- An in-depth introduction and analysis of relevant research with the marine energy harvesting were conducted through gradient classification.
- This review not only provided a deeper summary of the latest research progress, discoveries, and challenges, but also made a rational outlook on solutions to related issues and future development directions.

## Graphical abstract

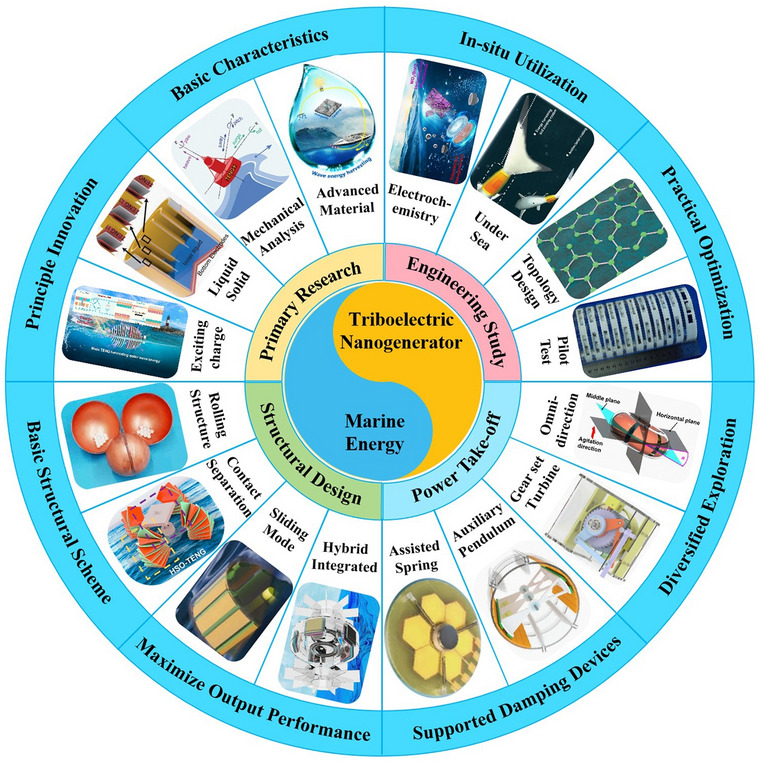

## Introduction

Since the industrial revolution marked by the steam engine, the human demand for fossil fuels has increased exponentially [1]. At the same time, due to the large-scale use of fossil fuels, billions of tons of CO_2_ are emitted into the Earth‘s atmospheric environment every year, which has a very serious impact on our human ecological environment [2]. Therefore, the countries around the world have formulated corresponding “carbon peak” and “carbon neutrality” development strategies, which were based on their own development [3]. Although the energy structure of the new era based on electrification heavily relies on pollution-free electricity, many countries around the world still rely on fossil fuels to obtain a large amount of electricity [4]. According to relevant research reports, vigorously developing clean renewable energy power systems and large-scale use of clean fuels will be important ways to achieve the dual carbon development strategy [5]. The ocean, which accounts for over seven-tenths of the global surface area, contains abundant renewable energy sources (water wave energy, osmotic energy, tidal energy, temperature difference energy, etc.) and is the most important place for humans to obtain large-scale green electricity [6]. Among them, marine fluctuations contain annual reserves of over 10,000 of TWh, which is one of the important targets for obtaining ocean blue energy [7]. In addition, combining the abundant water resources contained in the ocean and *in situ* utilizing the corresponding electricity by electrochemical method will be an important method for us to obtain low-cost clean fuels [8]. Importantly, it provides a new development direction by electrochemical technology to obtain the fresh water and remove the marine organic pollutants [9, 10]. Therefore, in the past century, the harvesting of marine energy has always been one of the crucial research directions in the energy field [11]. However, due to the characteristics of low-frequency fluctuations and vast distribution in the ocean, the inherent heaviness and high-frequency efficiency of traditional electromagnetic generator (EMG) result in low mass power density; it is difficult to meet the requirements of commercial applications for high mass power density of floating marine energy harvesting devices [12].

With the development of emerging technologies, the novel power conversion technologies, such as thermoelectric generator (TG), piezoelectric nanogenerator (PENG) and solar cells (SC), have been persistently developed [13–17]. Among them, the triboelectric nanogenerator (TENG) technology, which relied on the common principles of triboelectric and electrostatic induction, was invented by scientists [18]. Thanks to the characteristic of lightweight with the thin film material preparation method, it is benefiting for self-powered wearable sensor [19–21]. More importantly, combining with the inherent advantage of high efficiency in low frequency endows TENG an ultra-high mass power density, which is very suitable for constructing floating marine energy harvesting devices, which has attracted high attention from researching scholars in the researching area of marine energy [22]. Over the past 12 years, various research works on marine energy based on TENGs have shown explosive development [23]. Firstly, benefiting from the design integration of advanced energy capture devices, power take-off devices and other auxiliary devices, the energy capture and transmission efficiency of marine energy harvesting TENGs have been greatly improved [24]. In addition, with the rapid progress in the development of TENG, based on new materials, mechanical research, structural design and other research, the electrical output of TENG has been enhanced dozens of times, laying a solid technical foundation for the advancement of TENGs in the harvesting of marine energy [25]. According to the latest research, the volume power density of marine energy harvesting TENG can reach 1910 W m^−3^ by volume effect [26]. Finally, the design and research of corresponding topology integration schemes as well as large-scale devices have played a significant role in promoting the commercial application of this field [27]. More importantly, the tremendous advances have been made in the *in situ* development and design of advanced self-powered marine electrochemical systems, relying on the low-cost electricity obtained from high-performance marine energy harvesting devices [28]. For example, combined with corresponding power management devices, the fuel production rate clean fuel can up to 7.1 mL min^−1^. The above-mentioned TENG, which includes marine energy harvesting TENG and self-powered electrochemical system *in situ*, not only provides crucial technical guarantees for the industrial application of TENG, but also offers diverse options for achieving the sustainable development goals of human as soon as possible.

**Fig. 1:**
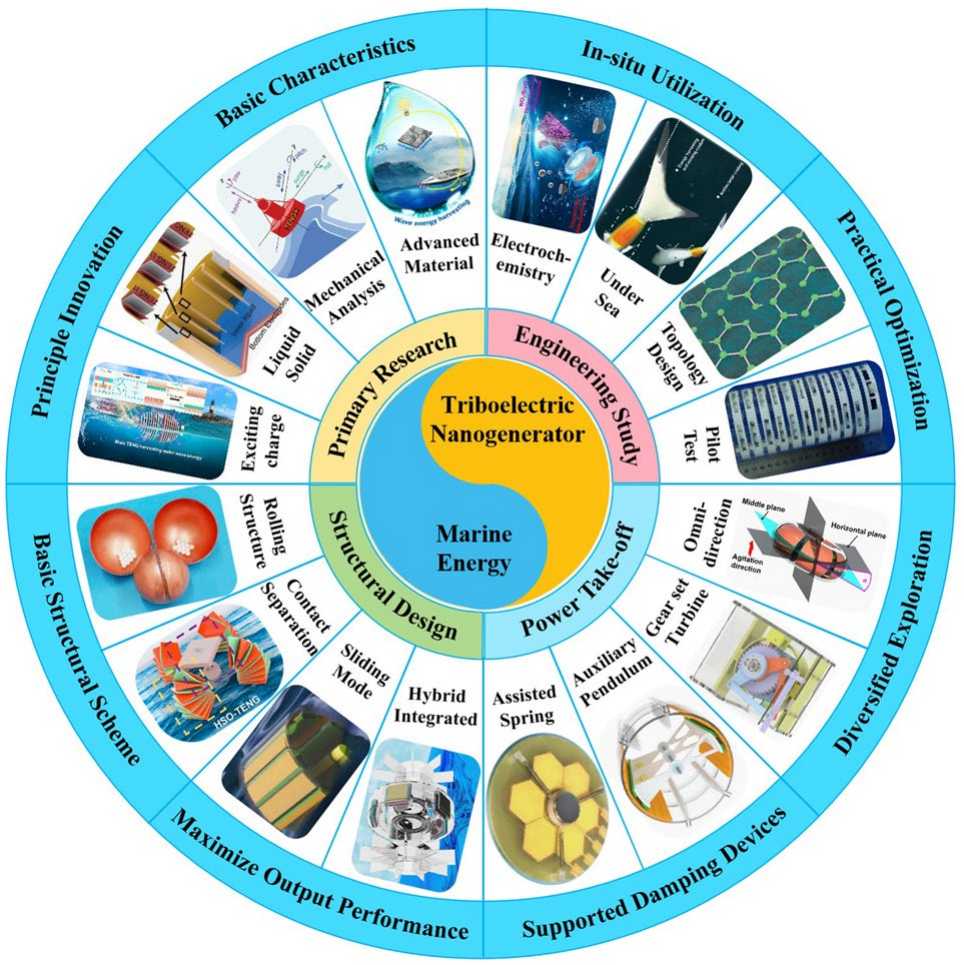
The TENG classification summary on marine energy and in situ clean electrochemistry. Reproduced with permission from Refs. [29, 35, 37, 39–42] Copyright 2020, 2020, 2018, 2021, 2019, 2022, 2020, 2020 Elsevier. Reproduced with permission from Refs. [30, 31, 33, 38, 43] Copyright 2024, 2018, 2015, 2021, 2022, Wiley. Reproduced with permission from Ref. [32] Copyright 2024, AIP Publishing. Reproduced with permission from Ref. [34] Copyright 2024, MDPI AG. Reproduced with permission from Refs. [36, 44] Copyright 2018, 2020, Springer Nature

The advanced researches of TENG's in marine energy and in situ clean fuel production system have been systematically and classified and summarized in the past decade. The overall framework of this review article is as follows. At first, we provided a detailed introduction to the basic information of TENG as well as the power conversion process and key points of the marine energy harvesting TENGs. In addition, an in-depth introduction and analysis of relevant research and applications were conducted through gradient classification: (i) The corresponding research works were divided into four major directions: primary research, structural design, power take-off, and engineering study. (ii) Based on the above four directions, the four research directions will be further classified into eight themes: basic characteristics, principle innovation, basic structural scheme, and maximizing electrical output. (iii) According to the specific research content, the relevant research work will be further subdivided into sixteen research topics: advanced material, mechanical analysis, liquid–solid, boosting power, rolling structure, contact separation, sliding mode, hybrid integration, assisted spring, auxiliary pendulum, gear set and turbine, omnidirectional design, pilot testing, topological structure, undersea energy, and electrochemical application, to facilitate readers to quickly understand the relevant research progress (Fig. 1) [29–44]. Finally, we not only provided a deeper summary of the latest research progress, discoveries, and challenges, but also made a rational outlook on solutions to related issues and future development directions. Therefore, this article will offer important research ideas for this field and novice researchers and accelerate to the sustainable development of the green energy system for humanity.

## Basic Information of TENG

**Fig. 2:**
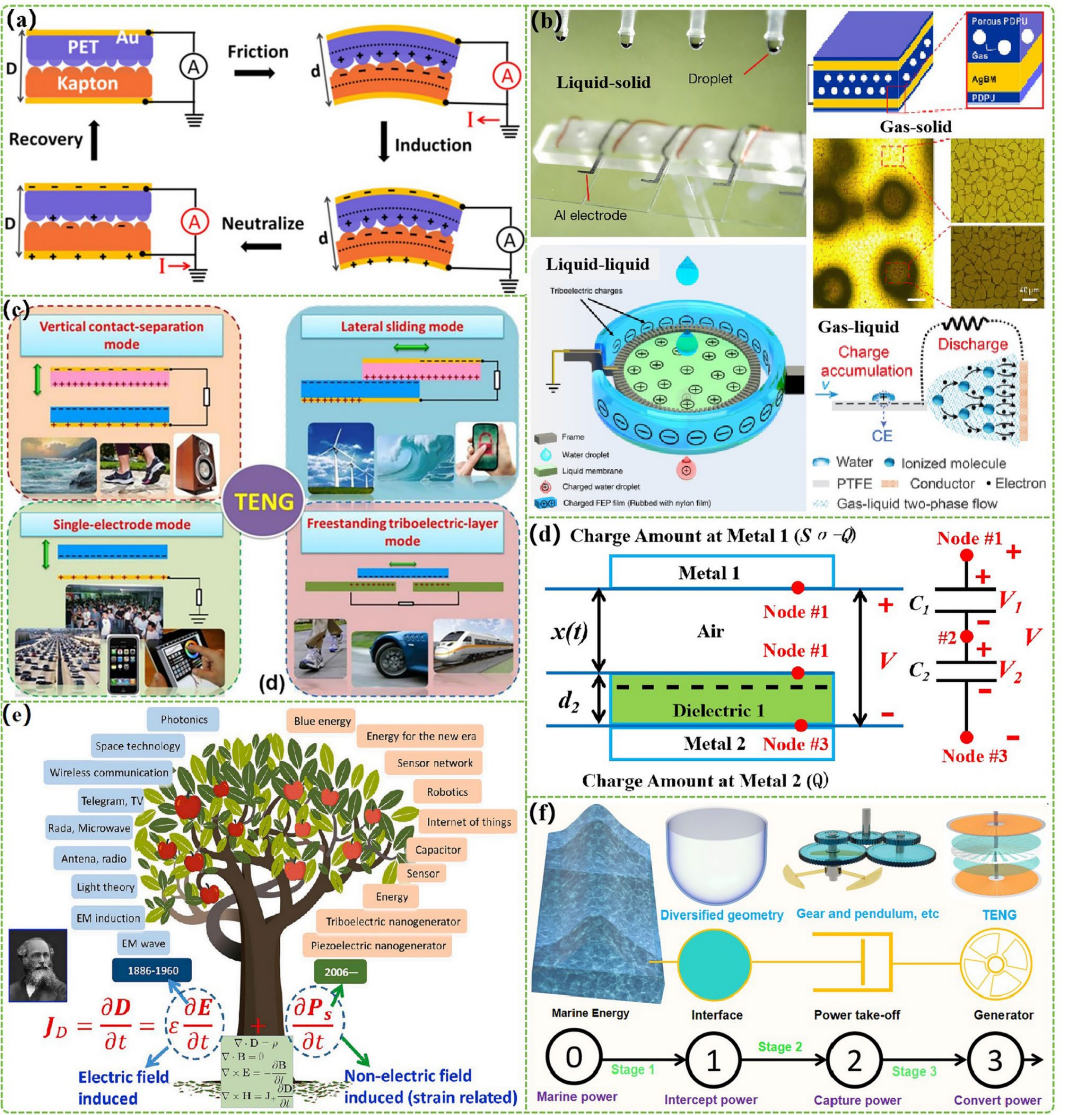
Schematic diagram of TENG with the basic information and the marine energy harvesting. **a** Structural diagram of first TENG. Reproduced with permission from Ref [46]. Copyright 2012, Elsevier. **b** Structure diagram of TENG composed of four kinds of material forms. Reproduced with permission from Refs [47, 48]. Copyright 2020, 2019, Springer Nature. Reproduced with permission from Refs [49, 50]. Copyright 2020, 2023, AAAS. **c** Schematic diagram of TENG with the four basic modes. Reproduced with permission from Ref [51]. Copyright 2014, The Royal Society of Chemistry. **d** Capacitance model diagram of the contact separation TENG. Reproduced with permission from Ref [52]. Copyright 2013, The Royal Society of Chemistry. **e** The development framework of a tree between Maxwell displacement current relation and TENG. Reproduced with permission from Ref [45]. Copyright 2020, Elsevier. **f** The power transmission chain of marine energy harvesting TENG’s

As an emerging electromechanical conversion technology, the work principle of TENG is relied on the coupling effect of triboelectric and electrostatic induction, which obtains corresponding electrical energy through a changing electric field. Its microscopic manifestation is the capacitive displacement current generated by the slight movement of electrostatic induction and bonding electrostatic charges [45]. The electrostatic effect caused by triboelectric is an extremely common natural phenomenon in people's lives, and this phenomenon has been present throughout the development of human society. Due to the fact that static electricity often poses some hazards to human production and life and may even cause serious accidents, previous scientific research has focused on how to eliminate triboelectric effect. In addition, the electrostatic effect caused by triboelectric effects is accompanied by another common natural phenomenon—electrostatic induction. Both the triboelectric effect and the principle of electrostatic induction have been rarely utilized in the past. It was not until 2012 that Wang et al*.* first proposed the concept of TENG by the above two principles when studying PENGs [46]. Its initial prototype is shown in Fig. 2a, which consists of two different dielectric material films (polyimide and polyethylene terephthalate) and metal thin film electrodes. During the process of bending and restoring a flat state, the two dielectric materials will undergo a contact separation process. Due to the different electron binding abilities of the two dielectric materials, the triboelectric effect will cause the polyimide film to carry a negative charge and the polyethylene terephthalate film to carry a positive charge during the contact process. Subsequently, a corresponding current will be generated between the two metal thin film electrodes. While two dielectric materials are separated, a relevant potential difference of two metal thin film electrodes will occur due to the principle of electrostatic induction, resulting in a current opposite to the contact process. Therefore, the TENG can make the corresponding mechanical energy convert into electric energy through the continuous contact separation process driven by mechanical force. This research has laid a solid foundation for further expanding the research work of more kinds of TENGs. As research continues to deepen, researchers have developed a variety of TENG. Accordingly, the medium composition can be divided into solid–solid, liquid–liquid, liquid–solid, gas–liquid, and gas–solid types (Fig. 2b) [46–50]. The structural design can be classified as contact separation, single electrode, sliding, and free-standing mode. The structural composition can be classified as single dielectric, double dielectric, and three dielectric structure (Fig. 2c) [51]. In order to more clearly study the operating principle of TENG with the physics face, a detailed theoretical research on the operating principle of TENG physics was established through Gauss theorem and the plate capacitance model, as shown in Fig. 2d [52]. For the contact-separated TENG, the mathematical relationship between the voltage (*V*), the transferred charges (*Q*), and the distance of the two triboelectric films (*x*) is obtained:

1 $$V = - \frac{Q}{{S\varepsilon_{0} }}\left( {d_{0} + x\left( t \right)} \right) + \frac{\sigma x\left( t \right)}{{\varepsilon_{0} }}$$

where *S* represents the surface area of the related electrode, *ε*_*0*_ represents the dielectric constant of vacuum, *σ* is the charge density of triboelectric material on the surface and *d*_*0*_ is the sum of the ratio between the thickness of the material and its relative dielectric constant between the two electrodes. Then, according to the definition of electrodynamics and related physical parameters, the intrinsic electrical output of the contact separation mode TENG can be obtained: the transfer charges (*Q*_SC_), short-circuit current (*I*_SC_), open-circuit voltage (*V*_OC_), and their relative capacitance size *C*:

2 $$Q_{{{\text{SC}}}} = \frac{S\sigma x\left( t \right)}{{d_{0} + x\left( t \right)}}$$

3 $$I_{{{\text{SC}}}} = \frac{{S\sigma d_{0} v\left( t \right)}}{{\left( {d_{0} + x\left( t \right)} \right)^{2} }}$$

4 $$V_{{{\text{OC}}}} = \frac{\sigma x\left( t \right)}{{\varepsilon_{0} }}$$

where *v(t)* is the speed at which the TENG operates. Therefore, it is obvious that the short-circuit current, open-circuit voltage, and charges density of the TENG are simultaneously quadratic, so the charge density will be the most important performance index to measure the electrical output of the TENG. Furthermore, to delve deeper into the basic physical laws of TENG, by introducing displacement vector into the classical Maxwell equations based on relevant theories, Wang et al*.* obtained the Maxwell displacement current equation after refinement:

5 $$J_{D} = \varepsilon \frac{\partial E}{{\partial t}} + \frac{\partial Ps}{{\partial t}}$$

where *J*_*D*_ expresses the displacement current, *ε* shows the dielectric constant, *E* is the electric field, *t* represents the time and *P*_*s*_ represents the newly introduced correction term of the displacement vector. The first term (*ε∂E/∂t*) represents the time-developing electric field and its induced polarization of medium, which is the theoretical basis of electromagnetic wave. Thus, it provides an important theoretical basis for various wireless communication and detection technologies. The second term (∂Ps/∂t) is the king term obtained after perfecting Maxwell’s equations, which expresses the displacement current of corresponding strain field (Fig. 2e). As the basis for the invention of nanogenerators, it has also had an important impact on the development of new communication technologies and self-powered systems in the Internet of Things [45].

## Power Conversion of TENG

It is the most important research direction in the field of efficient power transfer and conversion of TENG for marine energy harvesting as much as possible. According to the relevant research works, the power transfer chain and system structure of marine energy harvesting TENG can be simplified as shown in Fig. 2f. Firstly, the energy harvester in direct contact with the water intercepts the power of marine energy and converts it into the kinetic energy of the marine energy collecting device itself. Advanced design of energy harvester is an important basis for the efficient harvesting of marine energy and it is mainly designed by a variety of geometric structures involving fluid mechanics. Subsequently, the power take-off in the marine energy harvesting unit will capture the corresponding power through inertia and transfer it to the TENG. The inertial power capture and transfer energy of power take-off play an vital role in the effective harvesting of marine energy, which is mainly composed of gear and pendulum and other mechanical transmission components. Finally, the TENG uses the captured power by the power take-off to convert the corresponding power into electrical energy to achieve the corresponding energy harvesting. The TENG with the high electrical output, which includes materials, structure, and charge excitation and other aspects of research, has a decisive effect on the entire marine energy harvesting process. Therefore, improving the power interception of energy harvesters, the power capture of power take-off, the electrical output of TENG, and the system coordination among the three have an significant impact on the research of marine energy harvesting TENG. To more summarize the research progress of different aspects of marine energy harvesting TENG in detail, we adopt the method of combining size classification to make a more in-depth introduction.

## Primary Research

Because the primary research often plays a great role in promoting the whole research field, the relevant scientific research has carried out many studies in this researching aspect, which can be classified as basic characteristics and principle innovation research of marine energy harvesting TENG.

### Basic Characteristics

In the study of basic characteristics of marine energy harvesting TENG, triboelectric materials are closely related to the electrical output of TENG and would also give many excellent characteristics to TENG. The excellent mechanical properties conferred by the mechanical analysis of physics are an important prerequisite for the efficient harvesting of marine energy. Therefore, we had summarized the latest relevant research results from the two aspects of advanced materials and mechanical analysis.

#### Advanced Material

**Fig. 3:**
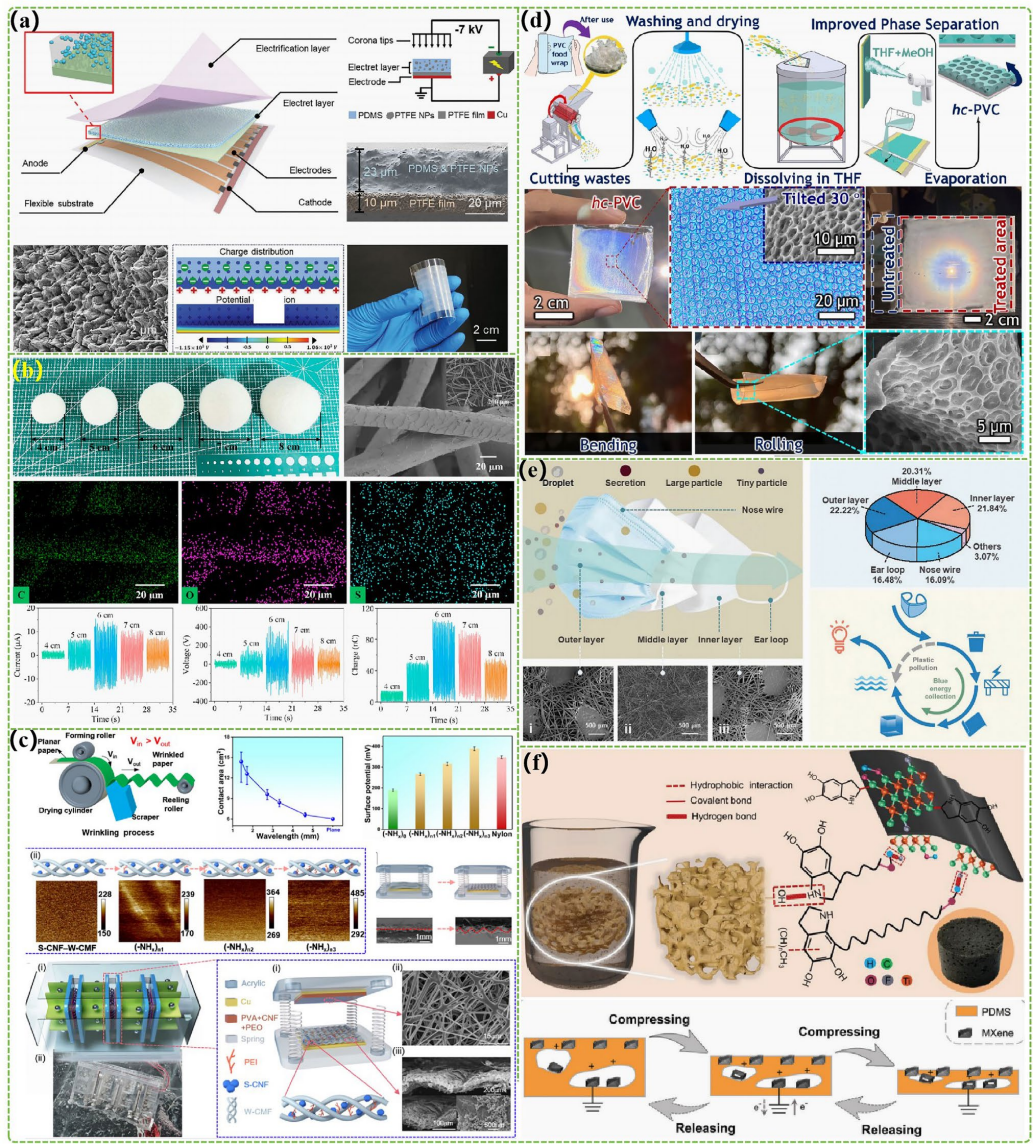
Advanced triboelectric materials of TENGs with the marine energy harvesting. **a** A nanocomposite electret layer enhanced triboelectric material. Reproduced with permission from Ref. [58] Copyright 2024, Wiley–VCH. **b** The excellent positive triboelectric properties and lightweight characteristics of wool. Reproduced with permission from Ref. [60] Copyright 2023, Elsevier. **c** A biodegradable negative triboelectric layer. Reproduced with permission from Ref. [61] Copyright 2023, Elsevier. **d** The surface of discarded PVC with the honeycomb structure. Reproduced with permission from Ref. [62] Copyright 2023, Elsevier. **e** Triboelectric materials based on the recycling of discarded medical masks. **f** The highly tribo-negative material by injecting MXene into the PDMS framework. Reproduced with permission from Ref. [65] Copyright 2023, Wiley-VCH

In order to improve the charge density of triboelectric material on the surface and add the electrical output of TENG, Jiang et al*.* connected the positive and negative electrodes of the high-voltage power supply to the copper electrode and the discharge needle, respectively, as well as fixed the discharge needle on the surface of PTFE [53]. The charge density of triboelectric materials on the surface was modified by polarization method to boost the electrical output of marine energy harvesting TENG. Subsequently, a silica gel/carbon black electrode with both high triboelectric and flexibility was developed to optimize the triboelectric efficiency of the triboelectric material through soft contact mode and doubled the electrical output compared with the relative wave energy harvesting TENG of the rigid structure of the copper electrode (the volume power density of 2.4 W m^−3^) [54]. Then, the electron affinity of silica gel was optimized by breaking the silicon–oxygen bond in silica gel and oxidizing free radicals under ultraviolet irradiation [55]. This method effectively improves the triboelectric performance of silica gel material and increases the electrical output of the wave energy harvesting TENG by about 10 times. In addition, a kind of rolling ball TENG by tribodielectric material with micro-/nano-surface structure was prepared by using the material synthesis method and its electrical output was more than 2 times higher than that of the TENG without micro-/nano-surface structure [56]. In terms of improving the stability of TENG, Wang et al*.* prepared a triboelectric composite coating material by combining acrylic resin material with fluorine material [57]. It has good micro-/nanostructure, super-hydrophobicity, and low surface energy, which could effectively improve the stability of TENG. Xu et al*.* fabricated a self-healing triboelectric material with microporous structure *in situ* on pure aluminum matrix by micro-arc oxidation technology [29]. Its self-healing properties enable TENG to effectively cope with aging, wear, and external damage during the process of application. A nanocomposite electret layer-enhanced triboelectric material was invented to effectively enhance the energy output and stability of the water wave energy harvesting TENG (Fig. 3a) [58]. The power density of the constructed TENG with an effective experimental size of 5 cm × 4.9 cm can reach 521 mW m^−2^. In the study of environmentally friendly triboelectric materials, Chen et al*.* designed a negative triboelectric material that is easily hydrolyzed, in which PLA is used as a polyester matrix and PLGA that is easily non-biohydrolyzed is used as an additional degradation accelerator [59]. This triboelectric material can be completely degraded in seawater within nine months. The TENG developed based on this material can not only achieve wave energy harvesting, but also pose no harm to the ocean. Based on the excellent positive triboelectric properties and lightweight characteristics of wool, a floating wave energy harvesting TENG based on wool ball was designed, which showed good wave following performance in marine environment (Fig. 3b) [60]. The polar layer with the superhydrophobic cellulose micro-/nanostructure has a positive triboelectric layer by the enhanced contact area and triboelectric density, Ding et al. prepared a biodegradable negative triboelectric layer by combining polyvinyl alcohol and polyethylene oxide water suspension with cellulose nanofibers by electrospinning method (Fig. 3c) [61]. Using this material to develop wave energy harvesting TENG has excellent practicability for the good moisture resistance of the porous structure of cellulose. At the same time, due to the low degradability of polymer materials, global plastic waste has cumulatively brought unprecedented challenges to environmental protection. Using waste polymer materials to construct high-performance wave energy harvesting TENG can not only effectively reduce environmental pollution, but also provide an important economic guarantee for the large-scale utilization of wave energy harvesting TENG. However, common polymer materials have very low triboelectric properties due to antistatic requirements. To effectively solve the above questions, Chau et al. found that etching the corresponding honeycomb structure on the surface of discarded PVC can improve its electrification efficiency, which obtains the advantages of low cost, environmental friendliness, and high time efficiency (Fig. 3d) [62]. The wave energy harvesting TENG, assembled with optimized PVC and aluminum, exhibits excellent electrical output, producing an open-circuit voltage of 340 V as well as an average surface power density of 2.8 W m^−2^. Compared with ordinary PVC, the above electrical output was increased by about 2.7 folds and 4 times, respectively. According to the special structure and material characteristics of disposable medical masks, a method was proposed to treat the bacteria in the film recovered from the masks and enhance the surface charge density of the film through a pulsed electric field system (Fig. 3e) [63]. In addition, the regenerated films were treated again by chemical and physical methods to obtain higher triboelectric properties. Through testing, it is easy to find that the volume power density of TENG with the wave energy harvesting reached 18.22 W m^−3^ and the medical mask materials accounted for 93.75% of the TENG. The above research is of large significance for the development of TENG technology for wave energy harvesting. Polydopamine was used to introduce highly tribo-negative MXene into the PDMS framework to improve the charge density of triboelectric materials on the surface (Fig. 3f) [64]. The introduction of MXene has a dual role, which can not only form triboelectric pairs with PDMS, but also serve as induction electroelectrode. The results of the micro-single-electrode TENG test show that the electrical output is improved by more than 5 times after modification. This material has good stability in the marine environment, so it can be widely used in wave energy harvesting and marine sensing.

#### Mechanical Analysis

**Fig. 4:**
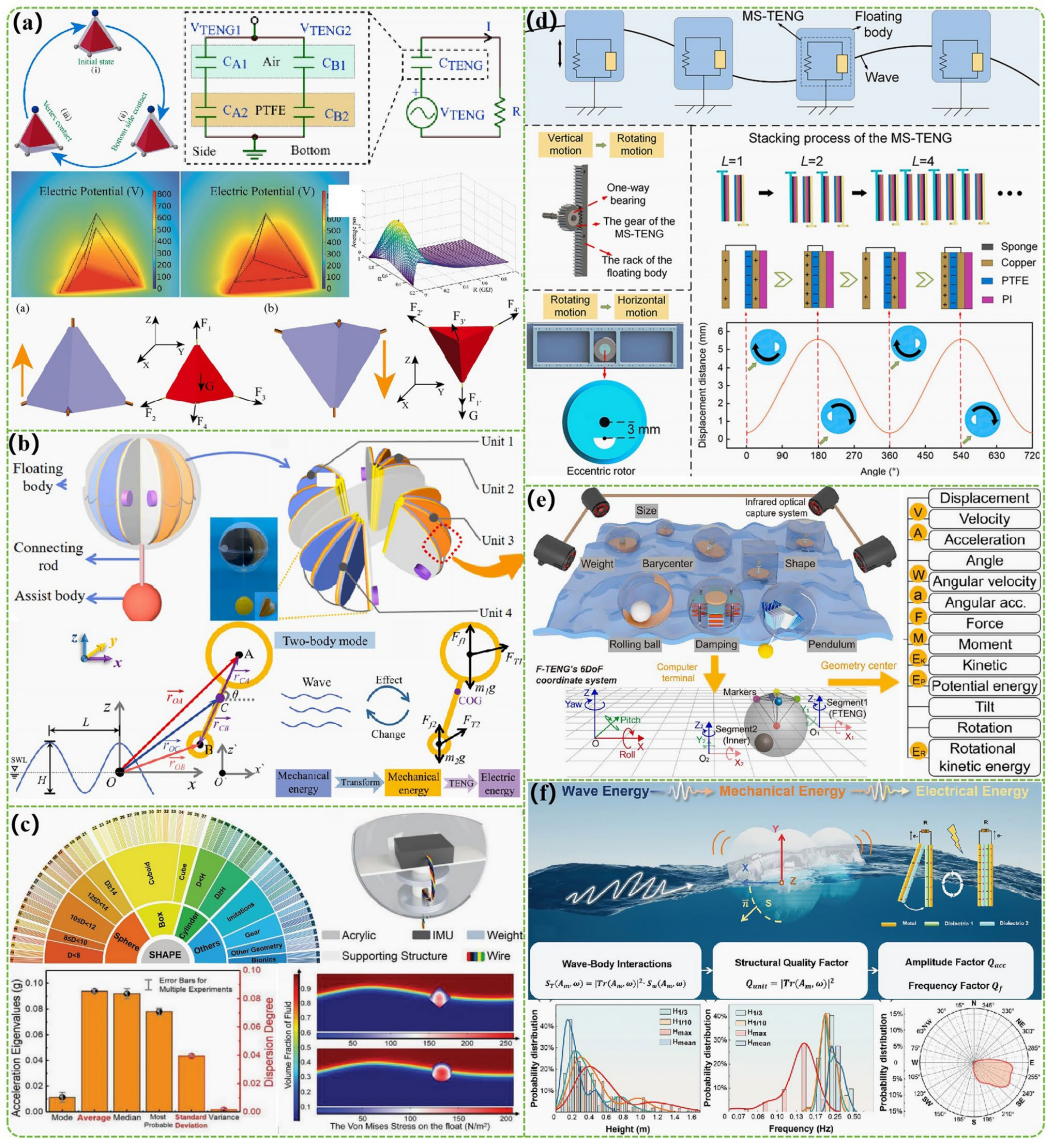
Mechanical analysis of TENGs with the marine energy harvesting. **a** The numerical simulation analysis and experimental testing of regular tetrahedron TENG. Reproduced with permission from Ref. [69] Copyright 2022, Elsevier. **b** The wave energy converting model by the linear wave theory in fluid mechanics and Morison equation. Reproduced with permission from Ref. [70] Copyright 2022, Elsevier. **c** Research on the energy harvesters of TENG based on references. Reproduced with permission from Ref. [71] Copyright 2022, Wiley–VCH. **d** A novel infrared optical harvesting system and precise numerical analysis methods for TENG. Reproduced with permission from Ref. [72] Copyright 2024, Elsevier. **e** A general model of the energy harvesting body for TENG. Reproduced with permission from Ref. [73] Copyright 2024, Elsevier. **f** A structural quality factor for quantitatively measuring the motion characteristics of TENG energy harvester and their ability to capture water wave power. Reproduced with permission from Ref. [74] Copyright 2024, Wiley–VCH

The dynamic model and the relationship *V-Q-x* theory were established to improve the electrical output of the rolling spherical [65]. Through theoretical research, it was easy to find that the radius ratio of the inner sphere to the outer sphere shell (*x*_*1*_), the thickness-to-radius ratio of the shell (*x*_*2*_), and the material density ratio of the inner sphere to the shell (*x*_*3*_) have significant effects on various output properties of the TENG. Among them, *x*_*1*_ has a dominant influence on the electrical output of rolling spherical TENG. As *x*_*1*_ increases, the peak power will increase and the internal resistance will decrease. Gravesen et al. built a mathematical model that can calculate the electrical output of a rolling spherical TENG in detail and quickly based on relevant mathematical studies [66]. The results show that this model can be used to guide the structure of TENG to achieve the best performance. In addition, compared with finite element analysis and other methods, this model has the advantages of simple calculation and is very suitable for the design of large-scale TENG network systems. Cestaro et al*.* obtained the time relationship between the buoy motion and its internal TENG output voltage by using the fast Fourier transform method and used the particle swarm optimization algorithm to construct the optimal equation between the buoy motion variable and the TENGs output voltage [67]. Although there are some errors in the relevant research, they are within the acceptable range and the more experimental data can be applied to obtain a better model for the study of wave energy harvesting TENG output power in a larger range. Wang et al*.* used ANSYS AQWA software to establish the hydrodynamic model of TENG for wave energy harvesting and adopted JONSWAP spectrum as the spectrum to simulate the electrical output of TENG under irregular waves [68]. Then the corresponding experimental prototype is made according to the corresponding simulation results. Combined with the real water wave test, the produced TENG showed excellent durability and good adaptability under the water wave drive of different amplitudes and frequencies. Based on the design of regular tetrahedron TENG, the side length ratio of the internal and external regular tetrahedrons in the whole TENG structure was continuously optimized by combining the method of numerical simulation analysis and experimental testing, thus maximizing the output power (Fig. 4a) [69]. Gonçalves et al*.* established the relation between the motion features of wave energy harvesting TENG and its electrical output based on the physical parameters of the TENG device through multiphysics field modeling analysis and diversified experimental testing [30]. Research has found that the energy generated by TENG is heavily dependent on the period of the waves and the speed of the moving elements in TENG, which are key factors affecting power output. Therefore, based on the characteristics of sea waves, optimizing the dynamics of the moving body to effectively adjust the contact velocity of the triboelectric layer is an important method to improve the sea state adaptability and electrical output of TENG for water wave energy harvesting. A water wave energy conversion model was established by the linear wave theory in fluid mechanics and Morison equation to study the conversion mechanism of wave energy harvesting and optimize the dynamic process of water wave driving TENG (Fig. 4b) [70]. Based on the relevant parameters of the installed TENG, its motion trajectory and water wave energy harvesting under water wave driving conditions were successfully calculated and the accuracy of the relevant model is verified in detail in combination with relevant experimental tests. The energy conversion efficiency of TENG after feedback optimization design can up to 14.5%. Based on published papers on water wave energy harvesting, the core parameters of TENG design were determined and corresponding energy harvesters under controllable variable conditions were fabricated (Fig. 4c) [71]. Xu et al. studied the interaction law between the geometric structure of floating TENG and water waves based on an inertial mechanics tester. Research has found that cubes have the highest absorption capacity, while the water wave response capability of spherical structures is directly related to water wave intensity, ball diameter, and center of mass. However, due to the better corresponding characteristics of energy harvester with circular interfaces for water waves in different directions, hydrodynamic simulation methods were utilized to study the water wave energy harvesting efficiency of such energy harvester with different shapes (Fig. 4d) [72]. The research results show that under the same volume and maximum cross-sectional area, the power capture efficiency of cylindrical energy harvesters is 70.14% higher than that of spherical structures. The researchers have also used a novel infrared optical harvesting system and precise numerical analysis methods to study the six-degree-of-freedom motion characteristics of various water wave energy harvesting TENGs in three-dimensional space under water wave excitation (Fig. 4e) [73]. A general model of the energy harvesting body for TENG water wave energy harvesting was established using corresponding dynamic calculations, statistical research, and ANSYS AQWA software simulation. The two important conclusions can be drawn: 1) Gradually increasing weight and number of faces, reducing size, and lowering the center of gravity will significantly reduce the plane’s rotation and oscillation capabilities. 2) Increasing the stiffness coefficient and supporting weight in the elastic mode, and adding appropriate auxiliary devices can effectively increase the power capture characteristics of entire device. Finally, Guo et al*.* proposed a structural quality factor for quantitatively measuring the motion characteristics of TENG energy harvester and their ability to capture water wave power (Fig. 4f) [74]. It can be further subdivided into amplitude structural factors that characterize the amplitude and frequency of energy harvester motion. Relied on a large number of experiments, the following important results were found: (i) compared with other types of structures, spherical structures have the best dynamic capture performance; (ii) cubes exhibit excellent power absorption in the vertical direction; (iii) when the vertical inclination angle of the energy harvesting body is about half of the complementary angle of the water wave front angle, its power absorption and conversion efficiency is the highest.

### Principle Innovation

To solve the various problems faced by the development of TENG, it will be a great important researching direction to develop a new TENG technologies through the innovation of new principles. Among them, liquid–solid TENGs have been widely studied because of good contact, very low wear and high energy converting efficiency. The effective output of electrical energy is the most important indicator to measure the performance of the TENG, and it is also indispensable to boost its output power as much as possible.

#### Liquid–Solid

**Fig. 5:**
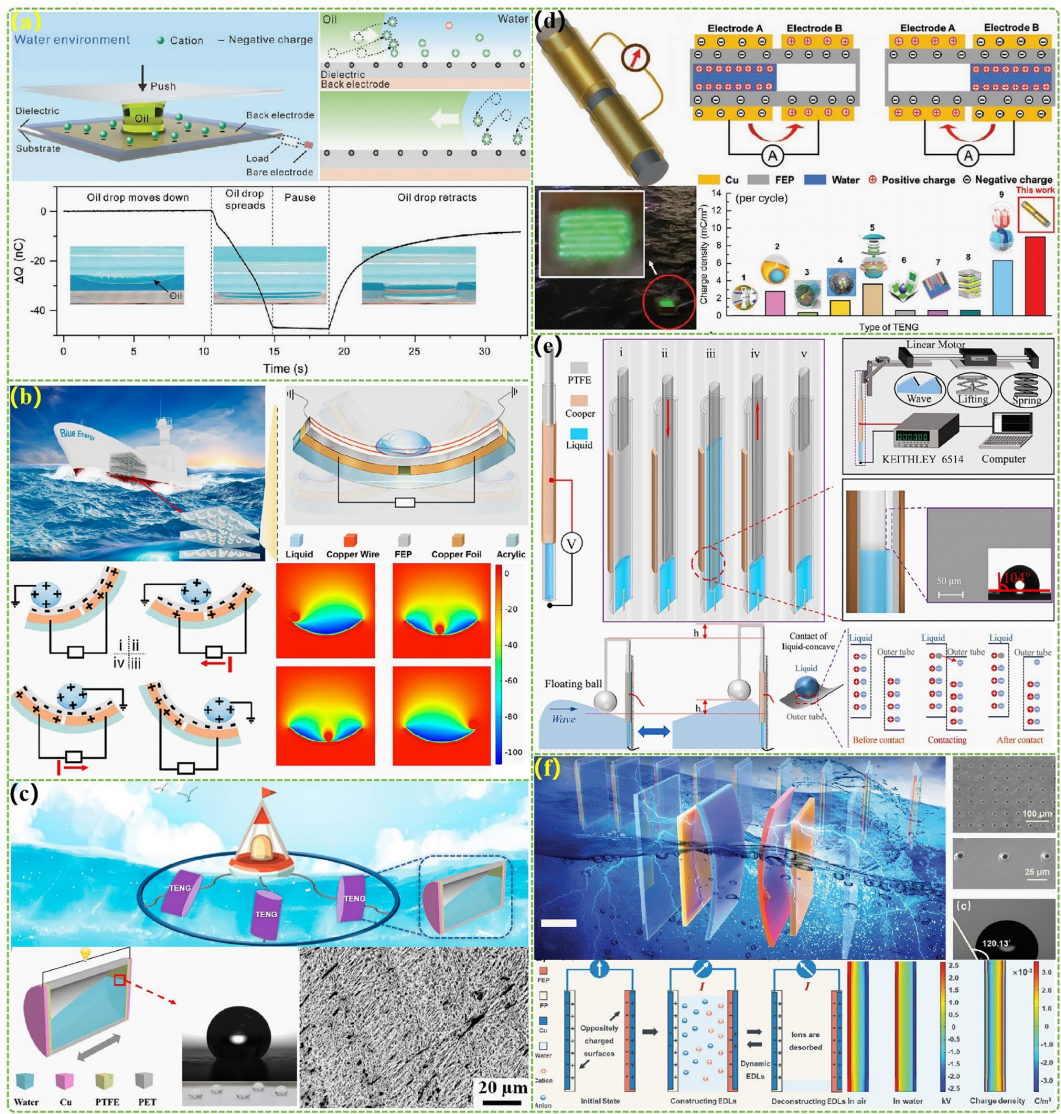
Liquid–solid TENGs for harvesting the marine energy. **a** Liquid–solid TENG with the oil phase. Reproduced with permission from Ref. [79] Copyright 2022, Wiley–VCH. **b** The high-output liquid–solid TENG with the seawater. Reproduced with permission from Ref. [81] Copyright 2020, American Chemical Society. **c** Liquid–solid TENG with the instantaneous contact structure. Reproduced with permission from Ref. [82] Copyright 2021, Elsevier. **d** Tube liquid–solid TENG with the high charge volume density. Reproduced with permission from Ref. [83] Copyright 2021, Wiley–VCH. **e** Liquid–solid TENG with the curvature effect. Reproduced with permission from Ref. [85] Copyright 2023, Elsevier. **f** A liquid–solid TENG based on a reverse dynamic double-layer structure. Reproduced with permission from Ref. [86] Copyright 2023, Wiley–VCH

A highly symmetrical three-dimensional spherical liquid–solid TENG has been designed to enhance the durability and energy harvesting efficiency of water wave energy harvesting TENG [75]. The inner and outer surface of its spherical shell was composed of two spherical liquid–solid TENG. Due to its novel three-dimensional symmetrical structure design and very small damping coefficient of liquid, this liquid–solid TENG can effectively harvest water wave energy with randomly changing directions and low energy density. Zhao et al. further boosted the output current of the entire liquid–solid TENG by preparing triboelectric materials with dense nanowire array surface morphology and combining them with segmented electrodes in a multiunit-integrated design [76]. The TENG with the size of 100 mm × 70 mm achieved a short-circuit current of 13.5 μA and a power output of 1.03 mW under driving conditions with the water wave height of 12 cm. The contact area and space efficiency of the entire liquid–solid TENG were greatly improved through the design of a circular array structure [31]. According to relevant research, thanks to the effective contact formed by the liquid–solid interface, under the same contact area conditions, the single period output energy of this liquid–solid TENG is about 48.7 folds that of the solid–solid TENG. A high electrical output liquid–solid TENG was designed by utilizing a Ū-shaped electrode composed of rod-shaped electrodes and U-shaped electrodes [77]. Since the design of Ū-shaped electrode can attract corresponding anions and effectively reduce their shielding effect on the surface of the triboelectric material with the negative charges, the electrical output of Ū-shaped TENG is about 10 folds higher than that of general single-electrode liquid–solid TENG. A novel liquid–solid TENG has been developed through the volume effect imparted by three-dimensional and arrayed electrode structure design, which can achieve an open-circuit voltage of 42 V, a short-circuit current of 4 mA and a high surface power density of 11.7 W m^−2^, respectively [78]. Subsequently, Zhou et al. increased the corresponding output voltage by about 40 times by introducing the related end electrode into the general tubular liquid–solid TENG to obtain the corresponding volume effect [26]. Meanwhile, through the study of liquids, it was obvious that the peak and average volume power density of liquid–solid TENG using NaOH solution can respectively reach 1910 and 459 W m^−3^. Through the study of introducing oil phase into the water–solid interface of liquid–solid TENG, it is found that controlling oil droplets on the surface of the dielectric material can generate corresponding current output on the electrode under the condition of water, which is related to the fact that the double electric layer on the oil–solid interface can drive the ion migration near the surface of the dielectric material due to the difference of the double electric layer on the water–solid interface and the oil-solid interface. The liquid–solid TENG can work in various water environments and can further enhance the output performance by adding charge on the surface of the dielectric material. Thanks to the need for no packaging, the TENG also demonstrated underwater energy harvesting and self-powered underwater sensing (Fig. 5a) [79]. In order to fully utilize seawater resources to construct corresponding liquid–solid TENGs, Jurado et al. developed a dual dielectric layer seawater type liquid–solid TENG with the high-output surface power density of 3.44 W m^−2^, which was based on a prepared double hydrophobic silicone rubber compound film [80]. The influence of the double layer on the electrical output of liquid–solid TENG was effectively avoided through using a rolling droplet structure (Fig. 5b) [81]. The electrical output of liquid–solid TENG was improved through increasing the water contact angle of micro-/nanostructures surface to optimize the sliding of water on PTFE surface and the corresponding TENG can achieve a short-circuit current of 2.68 μA. On this basis, the self-powered anti-corrosion technology was constructed through array design and electrochemical cathodic protection method (Fig. 5c) [82]. Wu et al. proposed a liquid–solid TENG with the simple structure and high charge output by deionized water, FEP tubes, and copper tape (Fig. 5d) [83]. The relative charge volume density can reach 9 mC m^−3^. A liquid–solid TENG with a high voltage output of 500 V was obtained through structural optimization design, which was based on the study of relative influence factors such as the length and diameter of the tube, the distance, the width of the electrodes and the connection method in the array [84]. In order to investigate the influence of contact surfaces with different curvatures on the electrical output performance of liquid–solid TENG, a liquid–solid TENG with the curvature effect was designed (Fig. 5e) [85]. After optimizing the electrode length, PTFE rod displacement, pipe diameter, concentration, and velocity, the TENG obtained an output voltage of 12 V. A liquid–solid TENG was designed by relying on a reverse dynamic double-layer structure for the effective converting of water wave power into electric energy (Fig. 5f) [86]. An open-circuit voltage of 60.0 V, a short-circuit current of 60 μA, and an average volume power density of 5.38 W m^−3^ were achieved by integrating the TENG array design. By optimizing the traditional tubular liquid–solid TENG model, which bases on the principle of interface charge transfer, we obtained a liquid–solid TENG for the water wave energy harvesting. The relative electrical output is 13 folds higher than traditional tubular liquid–solid TENG [87]. It achieved an output power of 17.74 mW, a short-circuit current of 900 μA, and an open-circuit voltage of 150 V.

#### Exciting Charge

**Fig. 6:**
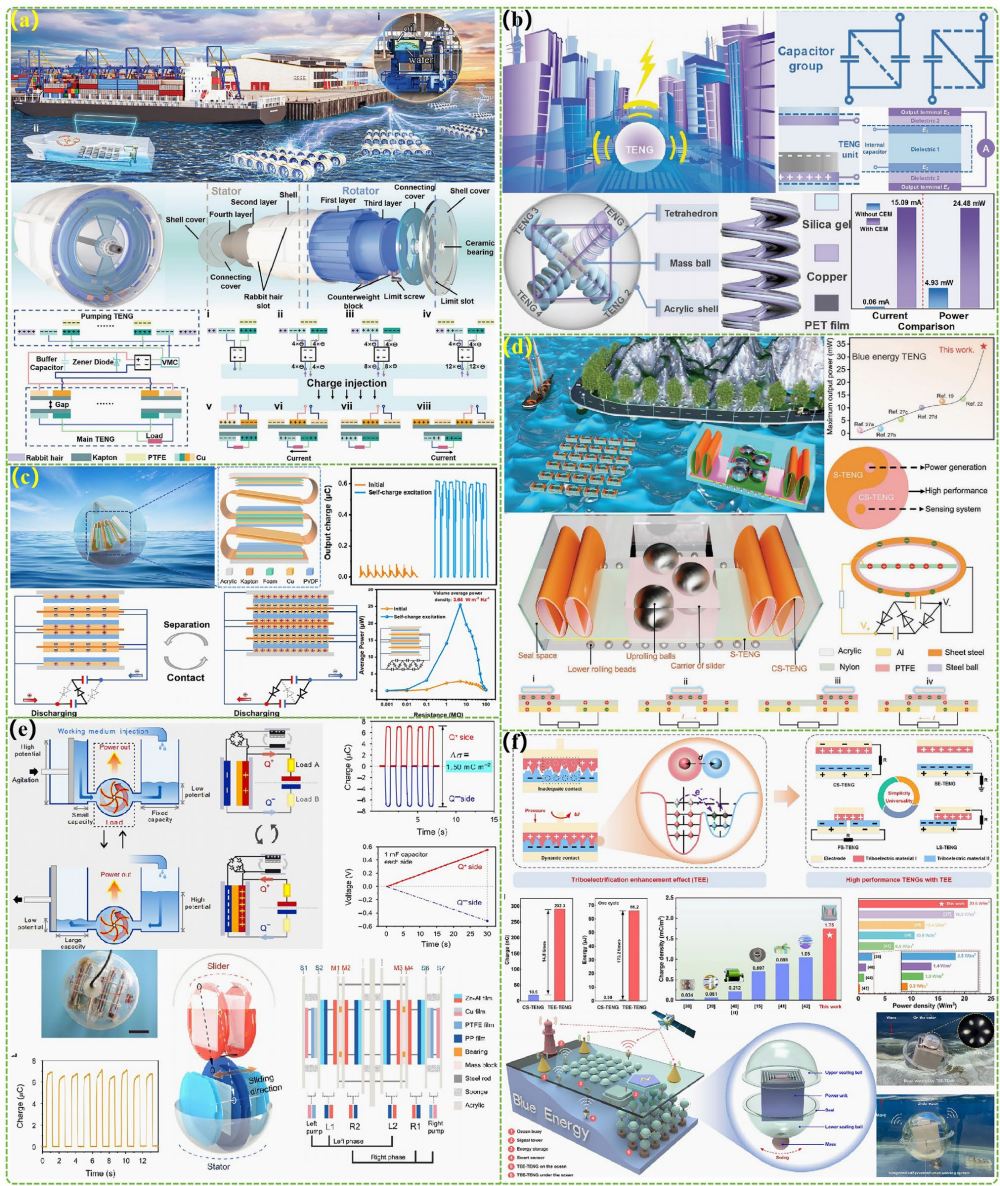
Boosting the harvesting of TENGs power from marine energy. **a** Non-contact TENG based on soft contact and voltage doubling circuit. Reproduced with permission from Ref. [92]. Copyright 2024, Wiley–VCH. **b** High-output TENG relied on capacitor series parallel mode switching. Reproduced with permission from Ref. [95]. Copyright 2022, Wiley–VCH. **c** Charge excitation TENG with the multilayer structure. Reproduced with permission from Ref. [96]. Copyright 2024, Elsevier. **d** Dual-mode TENG with the charge excitation. Reproduced with permission from Ref. [97]. Copyright 2023, Wiley–VCH. **e** A high-performance TENG by the external charge excitation and charge shuttle principle. Reproduced with permission from Ref. [98]. Copyright 2020, Springer Nature. **f** A contact separation mode TENG with high-output performance based on the principle of sliding friction. Reproduced with permission from Ref. [100]. Copyright 2024, Elsevier

In terms of increasing the effective output of TENG electrical energy, to boost the energy storage rate of TENG and avoid energy loss caused by its high-voltage output characteristics, a step-down and step-up circuit was designed by using diodes, LC oscillation circuits and buffer capacitors [88]. This circuit can effectively convert the irregular high-voltage output of TENG with the wave energy harvesting into a stable DC output, greatly promoting the development of water wave energy harvesting TENG in the field of marine Internet of Things. Liang et al. constructed a regular hexagonal structure water wave energy harvesting network using a spring-assisted multilayered spherical TENG as the basic unit [89]. By integrating the power management module TENG network, continuous and stable DC power output can be achieved by harvesting water wave energy under external load conditions, and its energy storage speed to the capacitor storage module has been increased by nearly 100 times. To improve the electrical output of TENG, Zhang et al. developed an instantaneous discharge TENG with high peak power by adding a self-controlled switch in the relating wave energy harvesting TENG [90]. TENG was composed of multiple parallel units in parallel, which can achieve a volume power density of 21.3 W m^−3^ and a maximum current of 30 μA. In addition, a whirling folded TENG with the high space efficiency and small electrostatic shielding was constructed using 3D printing and PCB technology [91]. Simultaneously, according to the pump TENG, the external charge excitation technology of two main TENG structures was studied and it was found that the charge excitation TENG working synchronously has more advantages and the corresponding electrical output was improved by tens of times compared to the original. To pertinently solve the wear of sliding TENG, an ocean wave energy harvesting rotating TENG with high space efficiency, high stability, and high electrical output was designed and installed by coupling a slight contact charge pump TENG and a non-contact main TENG, combined with a voltage doubling circuit (Fig. 6a) [92]. It can maintain good stability after 240,000 cycles and the high average volume power density can up to 3.56 W m^−3^ with the 0.8 Hz water wave driving. In addition, a charge excitation device with the voltage doubling circuit has been designed to significantly boost the output of water wave harvesting TENG [93]. The principle of synchronous storage and release of capacitor charges based on capacitor series parallel mode switching has enhanced the electrical output of single TENG by several folds. The TENG integrated with this charge excitation circuit achieved a power of 25.8 mW and the volume power density can up to 49.3 W m^−3^. More importantly, its current increased by 208 times to 25.1 mA. Subsequently, the researchers combined the above-mentioned charge excitation circuit with various other structures of TENG and found that this charge excitation circuit can also improve the electrical output of the corresponding TENG by several folds, thus it has good universality (Fig. 6b) [94, 95]. Meanwhile, through triboelectric material optimization and charge excitation circuit with the function of boosting electrical output and multilayer structure design with the high space efficiency, a TENG device was designed to efficiently harvest water wave energy as well as the average volume power density with 27.8 W m^−3^ was realized (Fig. 6c) [96]. Shan et al. reported a dual-mode output wave energy TENG, which utilizes a middle box to impact the contact separation mode TENG on both sides and an independent layer mode TENG with large steel balls inside the box to harvest wave energy (Fig. 6d) [97]. Two working mode TENG were concurrently used to harvest water wave energy and a high power output of 34.3 mW was achieved through an integrated charge mechanism circuit. Wang et al*.* developed an excellent performance TENG using external charge excitation technology and charge shuttle principle (Fig. 6e) [98]. It doubles the output charge by combining the negative and positive symmetrical capacitors with the main TENG and output the corresponding electrical energy by charging and discharging the two capacitors under the driving of the main TENG cycle. Furthermore, an ocean water wave energy harvesting TENG was fabricated by this principle and the volume power density can up to 30.24 W m^−3^ for its high charge density. Due to the high triboelectric performance that can be easily achieved through sliding friction, a synchronous TENG with self-charge excitation through intermittent sliding friction has been designed to realize the advantages of low friction loss and long-term high surface charge density [99]. It mainly supplements charges in a timely manner through the sliding friction of intermittent rotation of the pendulum to obtain high and long-term surface charge density and effectively improves the stability of the entire device by using contact separation mode TENG. Experimental tests have shown that this design can increase the charge density of TENG by 7 times and achieve a high peak power of 8.3 mW compared to TENG without intermittent sliding friction. Yu et al. also developed a contact separation mode TENG with high electrical output based on the principle of sliding friction (Fig. 6f) [100]. Compared to TENG without sliding friction optimization, it can achieve a charge density of 14.8 times and an output power improvement of 173.2 times. The wave energy harvesting TENG based on this design can achieve a volume power density of 20.6 W m^−3^ and a transfer charge of 2.2 μC, respectively.

## Structural Design

The structural scheme of TENG is not only closely related to its electrical output, but also has an great effect on the adaptability of TENG for harvesting wave energy. Therefore, researchers have conducted extensive research in structural design to meet the relevant requirements for efficient harvesting of wave energy as much as possible. The corresponding research content can first be divided into basic structural scheme and maximizing electrical output.

### Basic Structural Scheme

According to the basic motion characteristics of marine energy, the free-standing layer and contact separation modes of TENG are very suitable for the marine energy harvesting for their advantages of high electrical output and simple structure. Due to the large frictional force of the sliding free-standing layer mode TENG, which is not conducive to the harvesting of marine energy, researchers have invented the rolling structure free-standing layer mode TENG, which not only effectively solves this problem, but also further improves the durability of the free-standing layer mode TENG. We will provide a detailed introduction to the research in the following two aspects.

#### Rolling Structure

**Fig. 7:**
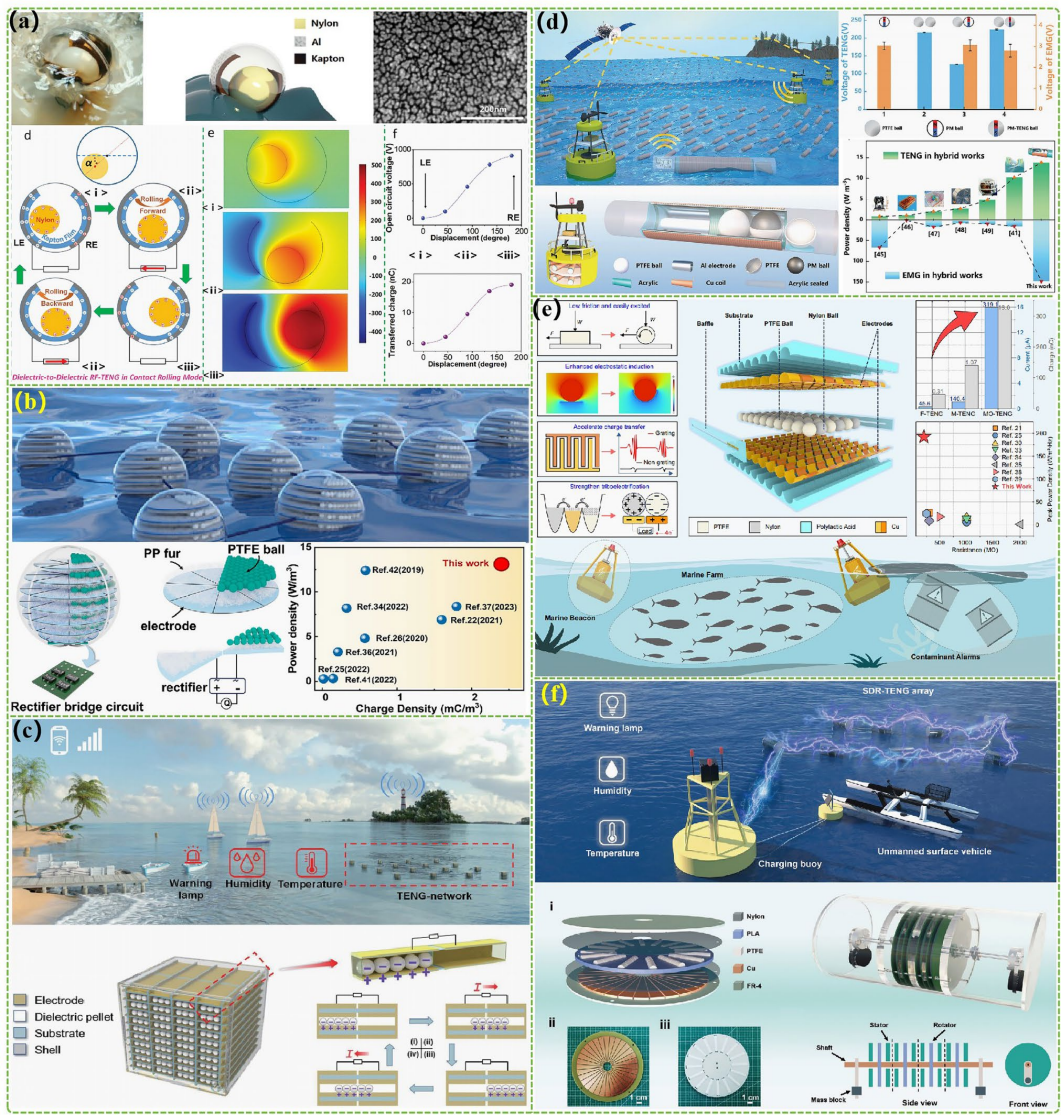
Rolling structural TENGs for the marine energy harvesting. **a** A rolling structure TENG with the function of adaptive barycenter. Reproduced with permission from Ref. [101]. Copyright 2015, Wiley–VCH. **b** Rolling structure TENG with the multipoint contact and optimized triboelectric materials. Reproduced with permission from Ref. [108]. Copyright 2024, Elsevier. **c** A rolling structure TENG by optimizing the shape of the external energy harvester. Reproduced with permission from Ref. [109]. Copyright 2023, Springer Nature. **d** Rolling triboelectric-electromagnetic hybrid generator based on permanent magnet polytetrafluoroethylene. Reproduced with permission from Ref. [110]. Copyright 2023, Wiley–VCH. **e** Rolling mode TENG with ultra-high output power by utilizing the principle of multitrack forked electrodes and positive and negative charge enhancement. Reproduced with permission from Ref. [111]. Copyright 2024, Springer Nature. **f** Stacked disk rolling TENG with high space efficiency. Reproduced with permission from Ref. [114]. Copyright 2024, Wiley–VCH

The first type of wave energy harvesting TENG with a rolling structure was designed in 2015, which mainly consists of a plastic ball shell as a sealing device, a pair of metal aluminum electrodes deposited on the inner wall of the ball shell, polyimide film pasted on the electrode surface, and independently rolling nylon balls (Fig. 7a) [101]. Its working principle is that nylon balls roll on polyimide film under wave drive, generating triboelectric effect, and then harvesting wave energy by generating relevant current between metal electrodes by the principle of electrostatic induction. This design has also become an important foundation for subsequent related research. However, due to the limited point contact area formed by the balls, the electrical output of this design is relatively low. Subsequently, the electrical output of TENG was significantly improved by transforming the original single ball into multiple balls with a larger contact area, resulting in a volume power density of 10.6 W m^−3^ [33, 102]. To further increase the contact area and optimized the spatial utilization of the entire device through three-dimensional structural optimization [103, 104]. The peak volume power density of the wave energy harvesting TENG can be increased to 32.6 W m^−3^ and the average volume power density also reaches 8.69 W m^−3^. At the same time, the magnetic grid design scheme proposed in this study provides important design ideas for subsequent large-scale applications. The triboelectric material research, track number, and unit integration method were proposed to ensure high triboelectric performance of TENG [105]. Meanwhile, by using the coaxial rotation design we ensure that all TENG units are in synchronous motion and combined with a nodding duck structure design to endow efficient power capture performance, enabling the entire TENG device to achieve stable and efficient power output. By using a 3D printing to install a rolling structure TENG with the function of adaptive barycenter, we can adapt to irregular waves by the continuously moving the barycenter position of the entire device and synchronously rotating the center slip ring to achieve random and efficient energy harvesting [106]. A simplified copper particle PTFE tubular TENG was applied to replace the previous dielectric material balls by using copper particles with better rolling characteristics [107]. By designing a multiunit structure, the space efficiency of the entire device has been further improved to achieve maximum electrical output in a unit volume. The designed TENG achieved a high current density of 41.4 mA m^−3^ under 1 Hz frequency driving conditions. Hong et al. improved the electrical output of the entire TENG by utilizing high triboelectric polypropylene fur to enhance the effective contact efficiency between triboelectric materials (Fig. 7b) [108]. Under the test conditions of 0.6 Hz frequency, the corresponding TENG obtained a volume power density of 13 W m^−3^. Systematically optimizing the shape of the external energy harvester and the filling amount of the internal rolling sphere continuously increases the electrical output of spherical rolling TENG (Fig. 7c) [109]. The average volume power density of installed prototype with the 10.08 W m^−3^ has been obtained under conventional driving conditions, and its peak volume power density (80.29 W m^−3^) was much higher than most other research works under actual water wave conditions. A triboelectric-electromagnetic hybrid generator based on permanent magnet polytetrafluoroethylene has been innovatively designed, which has high integration characteristics and ultra-high electrical output (Fig. 7d) [110]. TENG and EMG devices can achieve the output volume power densities of 13.77 and 148.24 W m^−3^, respectively, under extremely low-frequency driving conditions. In addition, the feasibility of harvesting water wave energy from different directions was explored through the design of a center diverging structure. Wang et al. designed a rolling mode TENG with ultra-high output power by utilizing the principle of multitrack forked electrodes and positive and negative charge enhancement (Fig. 7e) [111]. The average volume power density (10.92 W m^−3^) and the peak volume power density (185.4 W m^−3^) set new records for the current rolling mode TENG. To solve the problem of small contact area in point contact, replacing the original point contact with a cylindrical line contact design increases the electrical output of TENG [112, 113]. Meanwhile, the latter of the two studies also utilized a multiunit-integrated design to maximize the space efficiency of the entire device and optimized the electrical output of TENG using positive and negative triboelectric materials, thereby improving the electrical output of the entire TENG device by more than 10 times compared to traditional designs. For the stacked disk rolling TENG with high space efficiency, it can keep 95% of its original electrical output after 860,000 cycles of testing and thus has excellent durability (Fig. 7f) [114]. The device integrating four TENG units under simulated water wave conditions can achieve an effective volume power density output of 16.8 W m^−3^ and a current of 84.4 μA. To further improve the contact area of TENG, the researchers replaced line contact with surface contact formed by soft silicone spheres [115, 116]. After optimizing the hardness of the soft silicone material, the electrical output of TENG has been increased by more than 10 times compared to hard contact TENG. Finally, the output current frequency of the rolling structure TENG was improved and its internal matching impedance was reduced by subdividing more electrode pairs. This design also effectively increases the electrical energy output efficiency of the entire TENG device [117].

#### Contact Separation

**Fig. 8:**
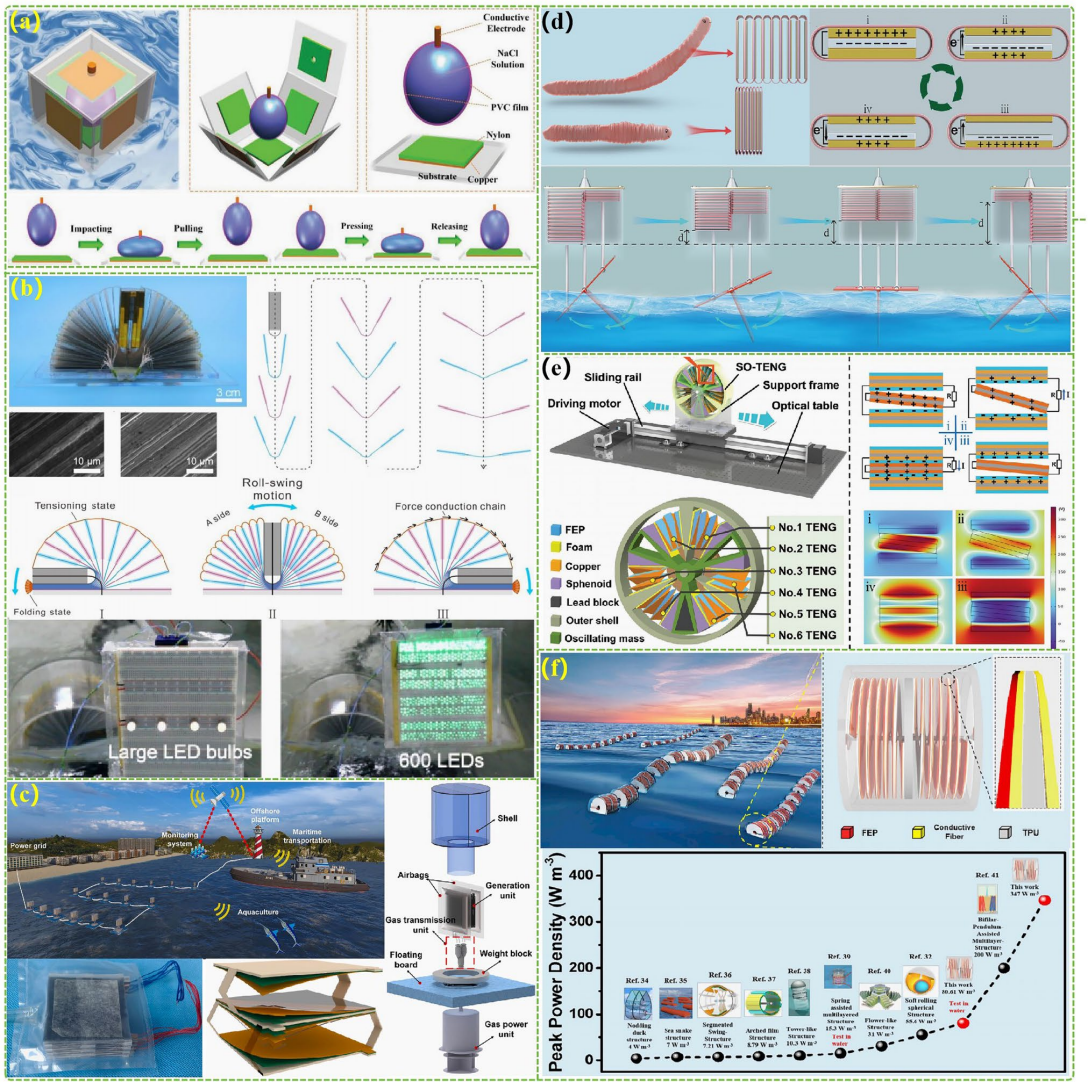
Contact separation TENGs for the marine energy harvesting. **a** The contact separation with the water balloon. Reproduced with permission from Ref. [118]. Copyright 2020, Wiley–VCH. **b** A multiunit-integrated open-book-like type TENG. Reproduced with permission from Ref. [120]. Copyright 2019, The Royal Society of Chemistry. **c** A multilayer TENG assisted by gas. Reproduced with permission from Ref. [122]. Copyright 2024, Elsevier. **d** The optimizing stability of multilayer unit integrated TENG inspired by earthworm. Reproduced with permission from Ref. [125]. Copyright 2024, Wiley–VCH. **e** Origami structure rotates TENG. Reproduced with permission from Ref. [126]. Copyright 2024, Wiley–VCH. **f** A spiral multilayer structured TENGs based on 3D printing technology. Reproduced with permission from Ref. [127]. Copyright 2022, Elsevier

Compared to the rolling structure TENG, the contact separation mode TENG has the advantage of easy surface contact and therefore has higher electrical output. Therefore, the first type of contact separation wave energy harvesting TENG was designed by the left and right slide of slider, which utilizes the contact separation process generated by the collision between the two sides of the slider and the baffle to achieve corresponding energy harvesting [53]. Simultaneously optimizing the working state of the entire device by adding lubricant to the sliding interface achieves higher energy harvesting efficiency. To completely remove the adverse effects of sliding friction on contact separation TENG, a contact separation mode TENG with the water wave energy harvesting was constructed through utilizing the soft characteristics of water balloon that are easy to generate surface contact through impact deformation and the zero friction characteristics of a pendulum structure (Fig. 8a) [118]. Under the same driving conditions, the total transferred charge of this TENG in one working cycle is 28 times that of the aforementioned slider structure TENG. Inspired by kelp structure and combined with flexible materials, a linear array integrated wave energy harvesting TENG was designed [119]. Under wave-driven conditions, the moving parts of each single TENG can work independently and engage in a process of contact separation with adjacent fixed parts to achieve the corresponding energy harvesting. By the inspiration of the dense stacking method of book paper, we design a TENG similar to an open book, which integrates a large number of TENG units in a certain space and greatly improves the contact area (Fig. 8b) [120]. Thanks to its excellent mechanical design, the TENG integrated with 50 units achieved a transfer charge of up to 26 μC, a volume power density of 7.45 W m^−3^ and a short-circuit current of 0.45 mA. Lei et al. were inspired by the vibration of butterfly wings to design a multiunit-integrated butterfly-type TENG, which can significantly harvest the potential energy of waves in the vertical direction and achieve a maximum output volume power density output of 9.559 W m^−3^ [121]. A multilayer TENG assisted by gas has been proposed, in which the gas-assisted device consists of two thin film structured airbags and a flexible material (Fig. 8c) [122]. The gas-assisted device can significantly enhance the effective contact area between triboelectric materials. The volume power density of TENG designed based on this structure can reach 20.4 W m^−3^. A multilayer structure TENG, which integrates multiple units in parallel, was constructed by using the zigzag structure design [123]. Due to its larger contact area, it achieved a high current output of 120 μA under water wave driving conditions. Zhang et al*.* significantly improved the charge density (210 μC m^−2^) of the multilayer structural TENG based on the modification of triboelectric materials [124]. Through optimizing the stability of multilayer unit-integrated TENG, we design the mechanical structures with up- and down-vibrations and worm structures, which was inspired by earthworm (Fig. 8d) [125]. The peak power of the O-shaped multilayer worm TENG structure produced is twice that of the zigzag multilayer structure TENG. Other researchers have further increased the contact area and effective contact between triboelectric materials by using origami structures as integrated substrates for contact separation of TENG, thereby optimizing the electrical output of the entire water wave energy harvesting TENG (Fig. 8e) [34, 126]. To continuously optimize the electrical output of multilayer structured TENGs, various spiral multilayer structured TENGs were designed based on 3D printing technology (Fig. 8f) [127, 128]. Among them, due to the serial spiral structure the ASM-TENG has obtained a larger contact area and space efficiency. Compared with the previous multilayer TENG, its space efficiency and output power have been greatly improved. The space efficiency rate and peak volume power density can reach 93.75% and 347 W m^−3^, respectively. The space efficiency rate of spherical TENG designed by the spiral multilayer structure also reached an astonishing 92.5%. Zhou et al. utilized unique pattern cutting and bridging techniques to design a four-helix multilayer TENG, which not has high electrical output and space efficiency, but eliminates the previous multilayer TENGs for the complex requirements of internal wires, foundation frames, and counterweights [129]. In addition, benefiting from the innovatively developed a multilayer TENG structure with a circular array based on flexible TENG units, this design can efficiently harvesting wave energy in any direction at 360 degrees [130].

### Maximizing Output Performance

In addition to basic structural design, maximizing the electrical output of TENG through the relevant structural scheme is also an effective method. As mentioned earlier, sliding friction can rapidly improve the charge density of triboelectric materials on the surface, thereby improving the electrical output of TENG. In order to overcome the adverse effects caused by sliding friction, researchers have also conducted many research works on the harvesting of ocean wave energy in sliding mode TENG. Furthermore, fully utilizing the advantages of different types of generators through hybrid structural design to increase the converting efficiency of marine energy is also an important solution choice.

#### Sliding Mode

**Fig. 9:**
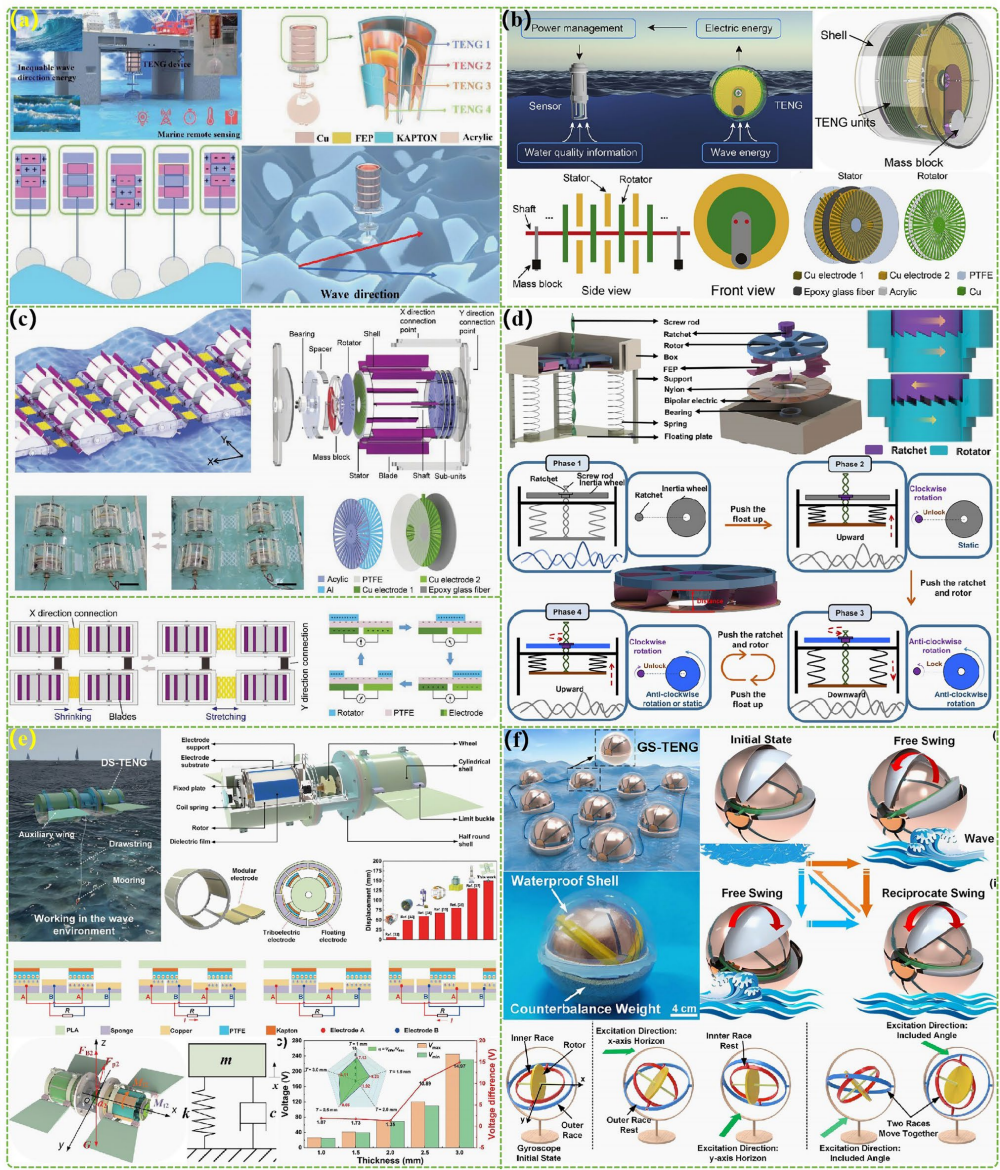
Sliding mode TENGs for the marine energy harvesting. **a** A multiunit-integrated circular tubular TENG. Reproduced with permission from Ref. [134]. Copyright 2024, Springer Nature. **b** A high-performance tandem disk-shaped TENG. Reproduced with permission from Ref. [136]. Copyright 2019, Elsevier. **c** A wheel type TENG network with super elasticity. Reproduced with permission from Ref. [137]. Copyright 2023, Wiley–VCH. **d** The sliding TENG based on elastic adaptive structure. Reproduced with permission from Ref. [138] Copyright 2023, The Royal Society of Chemistry. **e** The intersection of contact and non-contact TENG. Reproduced with permission from Ref. [142]. Copyright 2023, Springer Nature. **f** The multiple sliding TENGs based on fluff structure. Reproduced with permission from Ref. [143]. Copyright 2022, American Chemical Society

Through a dense disk track structure, we designed a TENG structure with the sliding friction of the disk side [131]. In order to ensure the efficient operation of TENG, a certain gap is reserved in the middle of the track for the disk and the wrinkled Al foil structure was utilized to control the contact area and effectively reduce friction. Compared with the rolling structure TENG, this design utilizes surface contact and sliding friction to greatly boost the triboelectric and electrical output of TENG. Under the driving of water waves at 0–0.5 Hz, this TENG device achieved an average volume power density of 1.05 W m^−3^, transfer charge density of 4.622 mC m^−3^, and peak volume power density of 14.71 W m^−3^. Zhong et al*.* further optimized the relation between the frictional force and electrical output roller structures. However, due to the increased roller structure, the space efficiency of the entire TENG device was reduced, resulting in a peak volume power density of only 5.47 W m^−3^. In order to fully leverage the advantages of free-standing mode TENG multiunit integration, a sliding TENG for harvesting potential energy in the vertical direction of waves was designed through material selection and structural design of multiple grating electrode pairs [132]. It can achieve high-frequency current output of 12 μA and peak power output of 54 μW under 8 cm high wave testing conditions. By further optimizing the structural design and adding pressure regulating devices to optimize the friction and electrical output of TENG, the electrical output of this TENG was improved by nearly 10 times under the same testing conditions [133]. Subsequently, an ocean wave energy harvesting TENG was designed by combining a hollow spherical shell with high buoyancy and a multiunit-integrated circular tubular TENG, which can obtain energy from water waves in any direction. Thanks to sliding friction, the device achieves a line power density of 730 mW m^−1^ driven by waves up to 4.8 cm high (Fig. 9a) [134]. Jung et al. utilized a structural design combining high-density TENG arrays and low-density array rabbit fur to obtain a water wave energy harvesting TENG with the function of doubling output frequency while ensuring efficient triboelectric performance [135]. A highest volume power density of 21.4 W m^−3^ was achieved in the frequency of simulative water waves with the 0.2 Hz. In addition, a high-output tandem disk-shaped TENG was designed through surface modification and structural optimization (Fig. 9b) [136]. Under the drive of water waves, this TENG, respectively, achieved highest and average power of 45.0 and 7.5 mW by the high spatial utilization and high-frequency electrical energy output, which are approximately 35 and 24 folds higher than the previous spherical TENG. The average volume power density of 7.3 W m^−3^ has set a new historical high for water wave energy harvesting TENG. In order to obtain higher driving force for sliding TENG and overcome its own high friction force, a wheel-type TENG network with super elasticity was proposed. The external blade array design of TENG can further efficiently obtain the power of waves (Fig. 9c) [137]. Meanwhile, the superelastic network structure can serve as a spring for storing wave energy, further improving the utilization of TENG for the water wave energy. According to relevant research, sliding TENG based on elastic adaptive structure can achieve good results between adjusting friction and electrical output. Therefore, this structural design was also utilized in the research of water wave energy harvesting TENG (Fig. 9d) [138]. To overcome the problems caused by the unidirectional rotation characteristics of TENG, the arched TENG was proposed to adapt to the oscillating characteristics of water waves [139]. This structure cannot increase the contact area but reduce the friction and achieve a two-way working mode, which in the test obtained a high maximal power density and an high average volume power density (8.79 and 2.16 W m^−3^). However, this design still has the problem of large friction due to the deformation of the material when the direction of movement of the TENG is switched. Therefore, a mechanical device integrated with a mechanical regulation device was designed to convert the external reciprocating linear drive into unidirectional rotational motion and then drive the sliding TENG with elastic adaptive structure to achieve wave energy conversion [140]. In addition, a water wave harvesting TENG based on the flabellum, spring, and rotary sliding structure design was proposed to maximize the durability of the TENG [141]. Under the driving of waves, flabellum will drive the rotor and stator of the TENG to contact, and then through sliding friction, the triboelectric material can obtain positive and negative charges with high charge density, respectively. Then flabellum makes the rotor and stator of the TENG separate from each other for a certain distance to swing freely under the action of the spring. This design greatly reduces triboelectric material wear and ensures high electrical output throughout the device. The design scheme of reducing the frictional force of sliding TENG through the intersection of contact and non-contact units has also been used in the research of water wave energy harvesting TENG, which has achieved high electrical output while having good device stability (Fig. 9e) [142]. To minimize the adverse effects of friction, multiple sliding TENGs based on fluff structures have been developed, benefiting from the good triboelectric of fluff under the less pressure (Fig. 9f) [143, 144]. Although these water wave energy harvesting TENGs have low electrical output, they all have good durability. Therefore, based on the cross design of positive and negative triboelectric materials and the rabbit hair brush structure, a non-contact TENG with high output and almost zero wear was developed. A high power output of 21.49 mW was obtained after system optimization of its design parameters [145].

#### Hybrid Integration

**Fig. 10:**
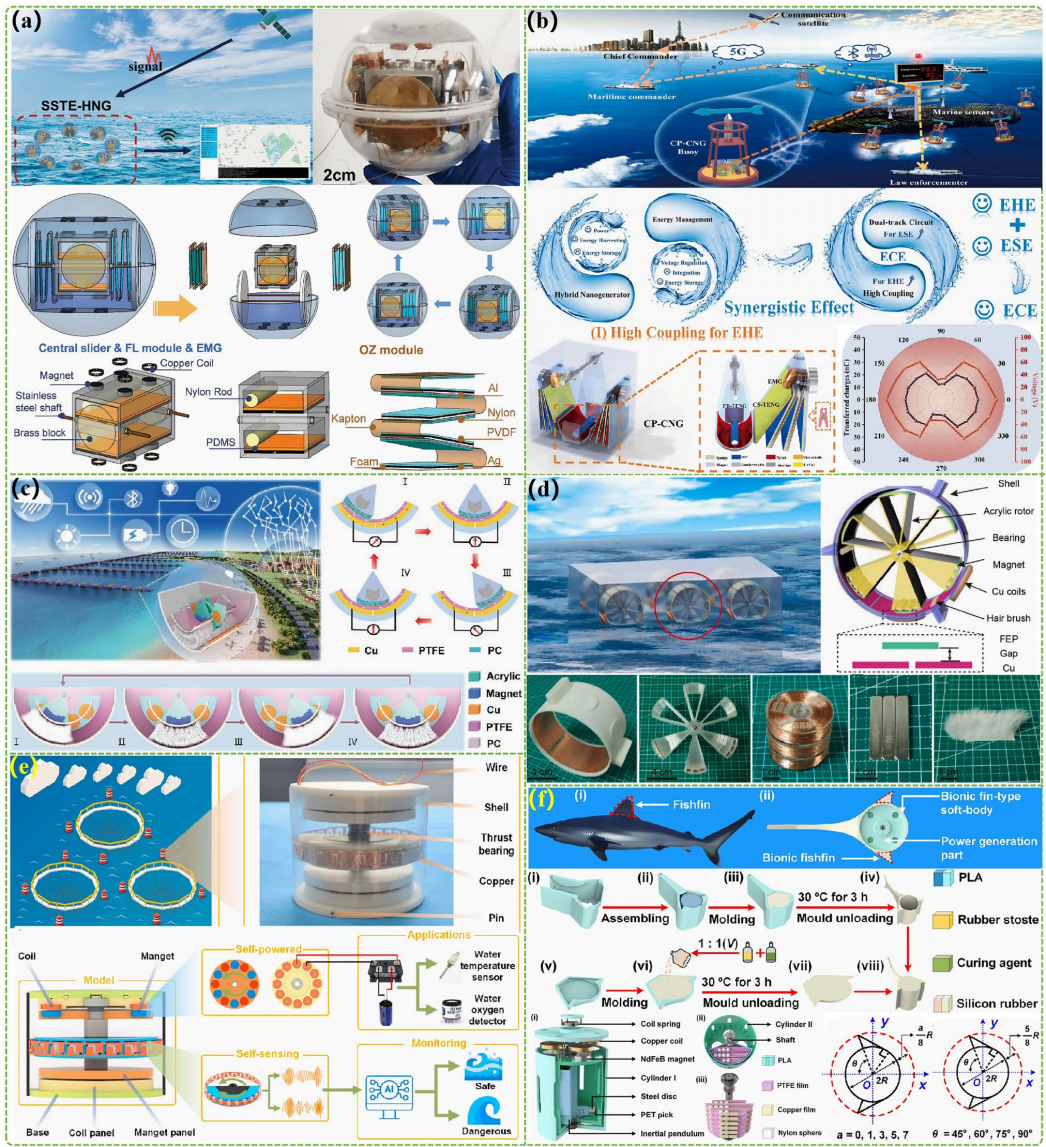
Hybrid generators for the marine energy harvesting. **a** Hybrid generators obtained by simple integration of different types of generators. Reproduced with permission from Ref. [148]. Copyright 2022, The Royal Society of Chemistry. **b** A triboelectric-electromagnetic hybrid generator with swinging structure. Reproduced with permission from Ref. [157]. Copyright 2024, Elsevier. **c** The hybrid generator based on the pile and Halbach array. Reproduced with permission from Ref. [159]. Copyright 2022, Wiley–VCH. **d** A hybrid generator composed of soft contact cylindrical TENG and swinging structure EMG. Reproduced with permission. from Ref. [161]. Copyright 2019, Elsevier. **e** A hybrid rotary generator through eccentric pendulum and rectification enhancement mechanism. Reproduced with permission from Ref. [162]. Copyright 2024, Elsevier. **f** A flexible biomimetic fin hybrid generator with a swinging rotating structure. Reproduced with permission from Ref. [163]. Copyright 2022, American Chemical Society

In the early stage of hybrid generator research, the relevant work was simply to integrate two or more different generators, and then work independently. For example, the hybrid generator is built through the integration of five power generation units: TENG, PENG, EMG, SC, and TG to maximize the harvesting of marine energy [146, 147]. Although this design has a higher electrical output than a single energy harvesting device, this simple integration is difficult to maximize the advantages of each unit through synergies (Fig. 10a) [36, 148–150]. Therefore, the subsequent related research direction immediately changed to a systematic and integrated design research. Firstly, in the research of tribo-electromagnetic hybrid generator, the spherical and cylindrical triboelectric materials in the original rolling structure TENG were replaced by magnets wrapped with the same triboelectric material and shape, and integrated coils with magnetic field changes generated by magnet movement were used to construct EMG with high-output characteristics in many studies [151, 152]. Due to the higher weight of the magnet, this design gives it more excellent water wave power interception ability, which can largely boost the wave energy harvesting efficiency of the hybrid generator. Among them, Ouyang et al. designed a triboelectric-electromagnetic hybrid generator with four generator units to greatly increase the electric output while effectively averting the loss caused by the friction [153]. A maximal volume power density of 79 W m^−3^ was obtained in the simulation test. To fully utilize the large inertia power capture and low center of gravity characteristics endowed by the weight of the magnet, researchers have designed various triboelectric-electromagnetic hybrid generators with swinging structures [154, 155]. The related designs not only effectively increase the space efficiency and electrical output of the entire device, but also greatly improve the stability of the entire device in complex wave environments (Fig. 10b) [156, 157]. There are also many representative research works in the research of sliding TENG through hybridization [158]. Han et al. designed a hybrid generator through the pile and Halbach array, in which the three TENGs constructed by double-sided pile not improved the space efficiency and electrical output of the entire device but effectively improved the durability of TENG (Fig. 10c) [159]. The EMG based on Halbach array gives the best weight composition of the whole device and enhances the overall electrical output. Based on relevant theoretical analysis and experimental comparison, the optimal connection mode of TENG and EMG was optimized. The relevant experimental results display that the peak volume power density of the whole hybrid generator can reach about 19 W m^−3^. A hybrid generator with a pendulum-type permanent magnets as mass blocks has also been developed. Thanks to the pendulum cone with permanent magnets and polyester fibers as the triboelectric layer, as well as the TENG Taiji-shaped electrode structure design, this hybrid generator can convert water wave energy in any direction [160]. In the research of rotating hybrid generators, a hybrid generator composed of soft contact cylindrical TENG and swinging structure EMG has been designed. By utilizing the sliding friction of flexible rabbit fur, the friction resistance was reduced and the durability of the device was enhanced, while the charge is pumped onto the surface of the dielectric material to improve the electrical output of TENG [145]. The hybrid generator based on EMG swing motion can generate more than 60 current outputs within 15 s under external driving. The optimized hybrid generator obtained a maximal volume power density of 10.16 W m^−3^ when driven by 0.1 Hz water waves (Fig. 10d) [161]. A hybrid rotary generator has been developed through eccentric pendulum and rectification enhancement mechanism. The rectification enhancement mechanism can convert the bidirectional rotation of the spindle into the unidirectional rotatory magnet flywheel, thereby driving TENG and EMG more efficiently for operation. Through experiments on a freedom platform with the six degrees, optimization was carried out with respect to the eccentric pendulum mass, resulting in a 36.11% increase in EMG power compared to the unmodified rectification enhancement mechanism (Fig. 10e) [162]. A flexible biomimetic fin hybrid generator with a swinging rotating structure has been proposed for harvesting low flow rate water energy (Fig. 10f) [163]. Specifically, it was originally driven by the vortex effect that the soft fins of the fish would swing and drive the entire hybrid generator to work. Thanks to the excellent hydrodynamic characteristics of the fins, this hybrid generator is very suitable for harvesting low velocity ocean currents undersea. For the liquid–solid TENG and EMG studies, Sun et al*.* built a hybrid generator by adding magnets to water, which is easy to flow [164]. Due to the low wear characteristics of the liquid–solid friction interface, this design has the advantage of reducing friction losses and expanding the size range of recoverable wave energy. It can achieve a current output of nearly 15 mA at 0.2 Hz operating conditions. A highly integrated tubular liquid–solid hybrid generator was developed by using the means of innovative structural design [165]. The electrical output of the whole equipment was largely increased by optimizing the length of the magnetic ball string and the diameter of the magnetic ball. In addition to hybridization with EMG, a hybrid generator coupled with TENG and PENG was designed based on the triboelectric and piezoelectric effects of special materials and finite element modeling analysis [166, 167]. Through experimental testing, it was found that compared with the separative PENG and TENG, the electrical output of the hybrid generator was increased by 2.24 and 3.21 folds, respectively. Two new triboelectric-solar hybrid technologies have been developed by integrating the solar power generation technology using the light shadow effect and the principle of photocatalysis [168, 169]. Although the electrical output of the relevant research is low, it provides a new method for the subsequent development of advanced TENG technology. Finally, in terms of more complex hybrid generator research, the researchers respectively proposed two TENG hybrid EMG and tribo-piezoelectric-electromagnetic hybrid related studies, which mainly focused on the integrated structural design of the entire device to optimize the overall spatial layout while greatly improving the power density output per unit [170–173]. According to the experimental results obtained from relevant studies, the maximum space efficiency rate and volume power density of the current marine energy harvesting hybrid generator can reach 91.9% and 358.5 W m^−3^, respectively.

## Power Take-Off

Power take-off, as an intermediate link in the harvesting procedure of marine energy, plays a very important bridging role. There is no need to consider how to efficiently capture the power of marine energy in related research, and it is also necessary to consider how to use the captured power to efficiently drive TENG work and ensure efficient electricity output. Supported damping devices can effectively increase power capture and output, while diversified exploration can explore more advanced power take-off designs, providing a continuous source of power to drive the sustained development of related research fields.

### Supported Damping Devices

According to relevant research, the damping-type power take-off design can not only effectively improve the capture of marine energy power, but also drive TENG to continue working by caching power, thereby achieving higher electromechanical conversion efficiency. It can also reduce the working conditions of the entire power take-off and achieve effective capture of smaller marine energy power. According to the relevant classification of mechanical devices, common damping structures can be divided into two categories: elastic and gravitational potential energy. Elastic potential energy is mainly used by springs, while the representations of gravitational potential energy utilization are the various pendulum.

#### Assisted Spring

**Fig. 11:**
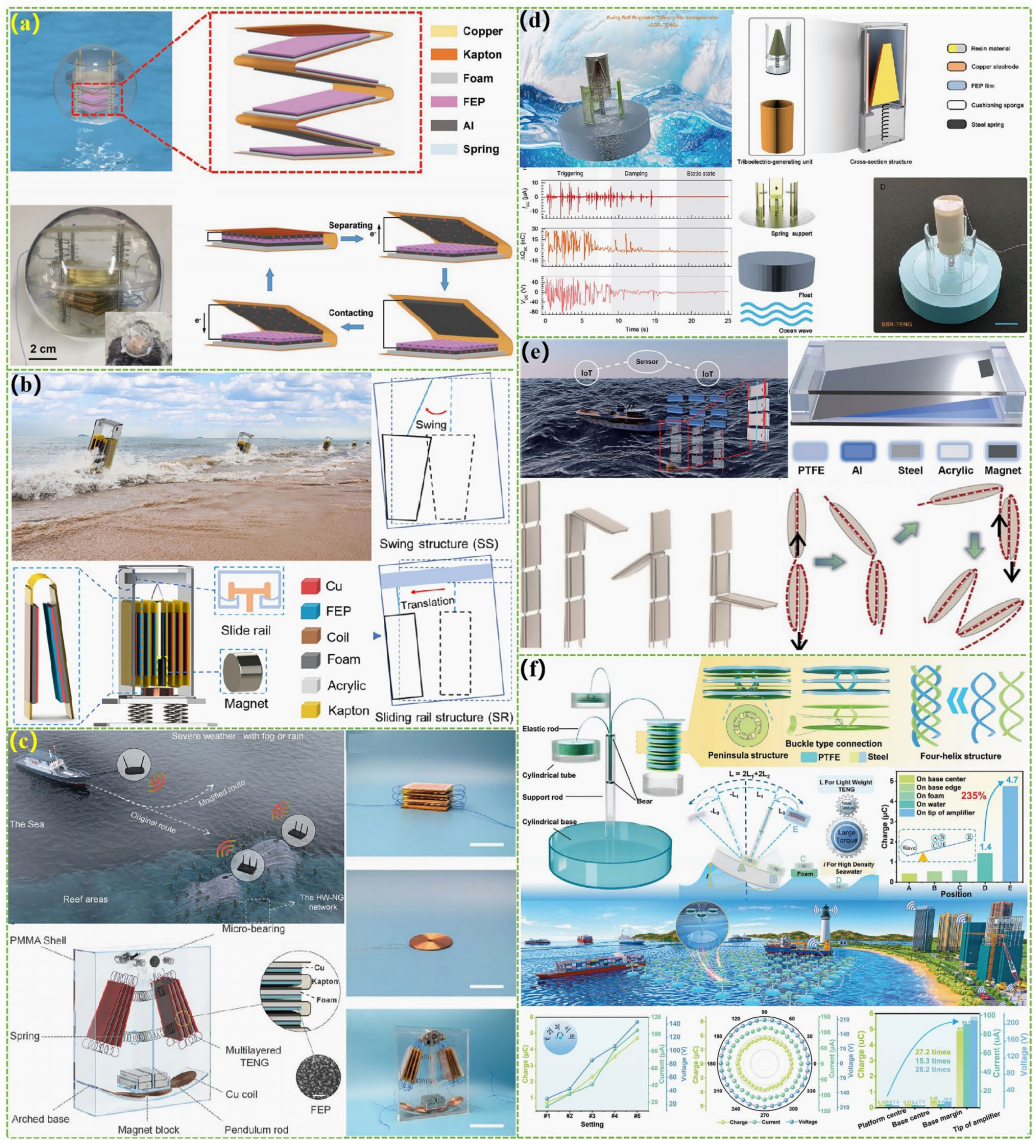
Spring-assisted TENGs for the marine energy harvesting. **a** A high-output wave energy harvesting TENG using a spring, guideway, and multilayer structure. Reproduced with permission from Ref. [123]. Copyright 2018, Wiley–VCH. **b** The swinging TENG by installing a spring base at the bottom. Reproduced with permission from Ref. [156]. Copyright 2022, American Chemical Society. **c** The multilayer structure TENG with the assisted springs and the swinging magnet. Reproduced with permission from Ref. [175]. Copyright 2021, Wiley–VCH. **d** A self-adjusting oscillation TENG based on a dual spring coupling system. Reproduced with permission from Ref. [176]. Copyright 2023, Wiley–VCH. **e** An elastic TENG based on cantilever beam. Reproduced with permission from Ref. [177]. Copyright 2023, Springer Nature. **f** TENG with the amplitude amplifier constructed by the elastic rod. Reproduced with permission from Ref. [129] Copyright 2024, Wiley–VCH

Driven by wave energy, the contact separation mode TENG can only make the less wave energy convert into electric energy for a short period of time and cannot effectively harvest the wave energy in the short time, resulting in serious wave energy loss in the process. As a very efficient mechanical energy cache device, the spring can convert a large amount of mechanical energy into elastic potential energy of spring in a short time and then gradually release it through mechanical damping. Therefore, the researchers introduced the spring into the design of the wave energy harvesting TENG as an important power take-off, which can produce multiple frequency electrical output through mechanical damping of the spring under a single wave drive [53]. By optimizing spring parameters and characteristics, the first type of spring-assisted wave energy harvesting TENG increased the conversion efficiency of the entire electrical energy by 150.3%. In order to further exploit the advantages of the spring, a spring-assisted TENG with the function of vibration frequency and amplitude method is designed. Benefiting from the advantages conferred by frequency and amplitude amplification, the TENG without this spring design has a tenfold increase in power output compared to the TENG [174]. In addition, it is found that after the introduction of spring structure, the TENG can not only obtain more intense working mode under the same driving conditions, but also work under smaller driving conditions to achieve the harvesting of smaller ripple water wave energy. A wave energy harvesting TENG by using the spring and honeycomb three-electrode structure was designed, which can capture the horizontal kinetic energy of the wave through the plane sliding mode and the gravitational potential energy of the wave by the vertical contact separation mode as well as can realize the advantage of any wave energy in a multidirection range [37]. It is found that the mechanical impedance characteristics of the spring-based damping device have a very important influence on the energy collecting efficiency of the whole device. By using system optimization to ensure that the TENG has resonance parameters, the whole wave energy collecting efficiency can be largely increased by making full use of the resonance state. Xiao et al. designed a high-output wave energy harvesting TENG using a spring, guideway, and multilayer structure (Fig. 11a) [123]. It can produce a good working state in the vertical direction and fully absorb the gravitational potential energy of the water wave in the vertical direction. A maximal volume power density with 15.3 W m^−3^ was achieved in the water wave test. A high-output TENG with multiple springs in series was designed for building self-powered ocean buoys with integrated power management, microsensors, signal acquisition and wireless communication modules [88]. Under 2 Hz water wave test conditions, the average surface power density output of TENG can reach 13.2 mW m^−2^ and drive the buoy device to achieve 15 m wireless information transmission. Sun et al. further increased the motion characteristics of the whole device by installing a spring base at the point of the swinging TENG and thus realized the effective harvesting of water wave energy with different wave height parameters (Fig. 11b) [156]. In the research of contact separation TENG through hybridization, the TENG with multilayer structure assisted by springs and the swinging magnet were integrated to construct a corresponding swinging structure, and four copper coils were installed at the bottom according to the motion characteristics of the magnet swinging to maximize the space efficiency of the entire hybrid generator (Fig. 11c) [175]. A wireless signal generation device with a transmission distance of up to 1.5 km was constructed by utilizing its high electrical output, combined with corresponding power management circuits and wireless communication modules. In order to adapt to the characteristics of random changes in wave frequency, a self-adjusting oscillation TENG based on a dual spring coupling system was used to efficiently harvest low energy density wave energy with randomness and short-term distribution characteristics (Fig. 11d) [176]. The MATLAB software was used to simulate and physically model low energy density wave energy. The self-adjusting structure of the dual spring system can generate higher oscillation frequencies and resonance effects, greatly improving the wave energy converting efficiency of TENG. The TENG unit utilizes a circular structural design to effectively harvest wave energy from the arbitrary direction. The electrical output of TENG using a self-adjusting structure was 5.8 folds higher than that of TENG without an integrated self-adjusting structure. In addition to the physical spring structure research, the researchers also carried out other elastic structures with spring function TENG research. An elastic TENG based on cantilever beam was proposed to efficiently convert the gravitational potential energy of waves into electric energy (Fig. 11e) [177]. The elastic structure design of the cantilever beam can largely improve the output frequency of the whole TENG. Relied on the material selection and structure optimization, the TENG realizes a maximum surface power output of 1.5 W m^−2^ per square meter of water in a simulated wave environment. In addition, a magnetic levitation triboelectric-electromagnetic hybrid generator by utilizing magnetic repulsion was designed to improve the overall space efficiency of the device by utilizing a magnetic spring structure, thereby enhancing the electrical output and the water wave energy harvesting efficiency through damping output characteristics [178]. Finally, Zhou et al*.* effectively increased the effective capture of wave power by installing the TENG unit at the top of the amplitude amplifier constructed by the elastic rod (Fig. 11f) [129]. The newly designed four helix TENG eliminates the requirements for internal wires, basic framework, and counterweights of the previous multilayer structure TENG due to the use of special installation techniques. The experimental results display that compared with TENG directly floating on the water surface, the operating amplitude and transferred charge of TENG based on amplitude amplifier can increase by 76% and 235%, respectively. More importantly, this new structural design achieves higher electrical output under the actual sea state test conditions, which fully proves the progressiveness of the relevant design.

#### Auxiliary Pendulum

**Fig. 12:**
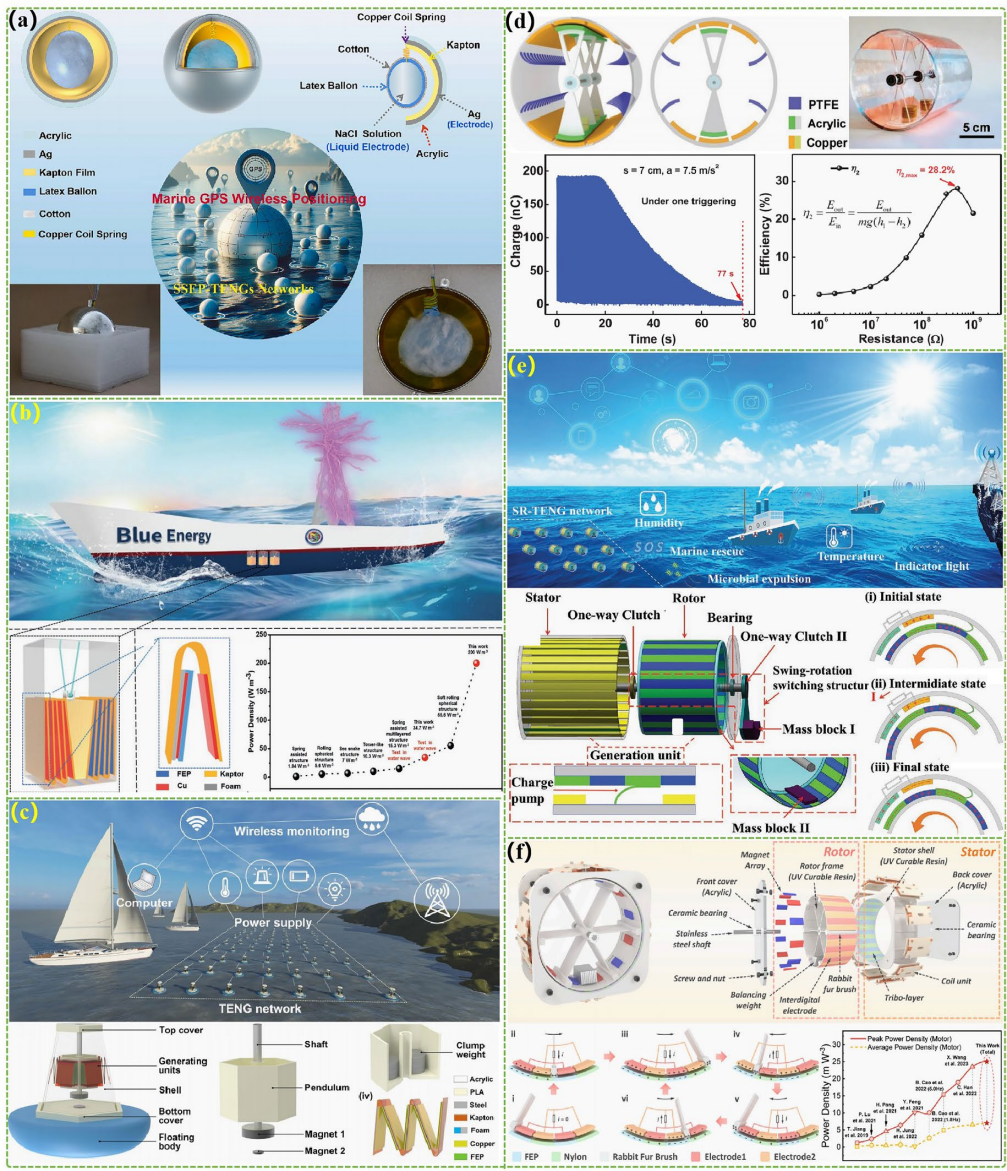
Auxiliary pendulum TENGs for the marine energy harvesting. **a** A swinging TENG with the flexible liquid ball. Reproduced with permission from Ref. [181]. Copyright 2024, Elsevier. **b** The multilayer structural TENG with the high charge density. Reproduced with permission from Ref. [124]. Copyright 2021, Wiley–VCH. **c** A swinging TENG with the high frequency by the introducing magnet. Reproduced with permission from Ref. [184]. Copyright 2024, Springer Nature. **d** A swing structure TENG with high energy conversion efficiency through air gaps and flexible electric brushes. Reproduced with permission from Ref. [185]. Copyright 2020, Wiley–VCH. **e** TENG by integrating a swing rotation switch and charge storage. Reproduced with permission from Ref. [187]. Copyright 2022, Wiley–VCH. **f** A swinging hybrid generator with a high power density output. Reproduced with permission from Ref. [145]. Copyright 2024, Elsevier

Compared with spring, pendulum has higher energy storage efficiency and damping characteristics, so TENG based on pendulum structure has more significant advantages. Shortly after the TENG was invented, the first water wave that could harvest the TENG was designed using a single pendulum structure. It consists of a single pendulum ball wrapped with PA and a PTFE-coated spherical shell, and converts water wave energy into electrical energy through the contact separation process caused by the impact of the pendulum and the spherical shell [179]. However, the small contact area due to point contact greatly limits the electrical output of such TENG. Therefore, the TENG, based on a solid–liquid elastic pendulum, was designed for a full range of water wave energy harvesting [180]. Since the flexible liquid ball can obtain a larger contact area through deformation during impact, the corresponding electrical output was improved several times (Fig. 12a) [181]. In this way, the pendulum cone with spherical structure was transformed into a circular table structure and the corresponding geometric structure design was combined to achieve a good linear contact and the electrical output of the TENG with single pendulum structure is again improved by nearly 10 times by integrating the flexible toroidal TENG array to obtain a larger contact area [182]. By using the seesaw mode of the vessel in the wave, a bifilar pendulum structure of the multilayer structure TENG was designed. In the related design, the bifilar design of the pendulum can make it achieve good unidirectional swing characteristics, the corresponding geometric mechanism design can ensure good surface contact, and the multilayer structural design and triboelectric material optimization make the entire TENG have ultra-high output power (Fig. 12b) [124]. As a result, the whole water wave energy harvesting device achieves the higher peak volume power density of 200 W m^−3^. Compared with previous corresponding water wave energy harvesting TENG, this electrical output was increased by 1–2 orders of magnitude. A TENG based on the principle of seesaw equal-arm lever is proposed to solve the problem that TENG performance output is easily affected by its structure [183]. Taking advantage of the stability bestowed by its rigid moving structure design and the optimized testing of simulated water wave performance, the TENG was designed the maximum current and voltage outputs can up to  72 μA and 1055 V. In order to overcome the problem that single pendulum structure cannot drive multilayer TENG effectively to collect water wave energy in different directions, a multilayer TENG was proposed for collecting multidirectional wave energy by installing multilayer TENG in different directions and multiedge structure design of pendulum cone (Fig. 12c) [184]. The magnetic field was introduced by the magnet to transform the simple swing of the pendulum into three-dimensional space motion, and the efficiency of the entire device with the wave energy converting was increased by boosting the operating frequency of the TENG. Relevant tests show that the electrical output of the TENG increases by 73% after optimizing the whole system by establishing a spring model. A swing structure TENG with the high energy harvesting efficiency was designed through air gaps and flexible electric brushes. Due to the minimization design of frictional resistance, the entire TENG has more sustained electrical energy output and stability (Fig. 12d) [185]. It can achieve 88 damping oscillations under single driving conditions, resulting in high energy conversion efficiency, and can maintain stable electrical output in 400,000 continuous stability tests. Subsequently, relevant researchers further optimized the electrical output of the entire TENG by increasing the number of integrated TENG units through subdivision and utilizing super lubricated bearings. It can achieve energy output of over 5 min under single excitation conditions and achieve a total energy converting efficiency of 23.6% [38]. Under the corresponding water wave testing conditions, an average power of 0.74 mW and a peak power of 6.2 mW were obtained. In addition, an internal oscillating cylindrical TENG with dielectric material suspended on the stator electrode is designed by using the supporting action of the bearing component [186]. Based on the continuous rotation obtained by the internal rotor suspension mode design, the installed TENG device can achieve a damping relaxation electrical output of approximately 85 s and can generate more than 1000 current outputs under a single wave drive. Meanwhile, a TENG that integrates a swing rotation switch and charge storage was also designed to convert the power of waves into electric energy (Fig. 12e) [187]. The swing rotation switch can swing and convert into a single rotation, while the charge storage/release design allows the TENG to realize the highest output under conditions of low friction. The installed TENG achieved a maximal volume power density of 15.4 W m^−3^ and long-term stability. A hybrid generator with a high power density output swing structure was constructed by timely supplementing triboelectric charges through the design of a structure that achieves higher electrostatic induction performance and flexible soft contact by crossing positive and negative triboelectric materials (Fig. 12f) [145]. It can achieve a maximal power of 71.59 mW and a maximal volume power density of 25.08 W m^−3^ under the motor driving conditions at a frequency of 1.0 Hz. Under the simulated water wave driving conditions, an average power of 13.28 mW and a maximal power of 47.52 mW can be obtained, demonstrating excellent water wave adaptation characteristics. Finally, based on the excellent electrical output and stability of the origami structure TENG, a swinging structure origami TENG was used to construct a water wave energy harvesting device by adding a weight at the bottom to build a corresponding oscillating body [126]. The reciprocating motion of its oscillating body achieves effective interception of water wave power, while the swinging structure greatly increases the capture and transmission of water wave power for power take-off. The folded TENG, as an integrated unit, improves the contact area of TENG per unit volume, thereby enhancing the electrical output of the entire device.

### Diversified Exploration

In addition to the traditional damping power take-off research that can increase the efficiency of TENG's harvesting of marine energy, it is also of special significance to use diversified and innovative methods to solve other problems faced by TENG in terms of power take-off for marine energy harvesting. The study of the mechanical structure of gears/turbines can largely increase the electrical output stability of TENG, while the study of omnidirectional energy harvesting characteristics plays a crucial role in promoting the practical application of TENG in marine energy.

#### Gear Set and Turbine

**Fig. 13:**
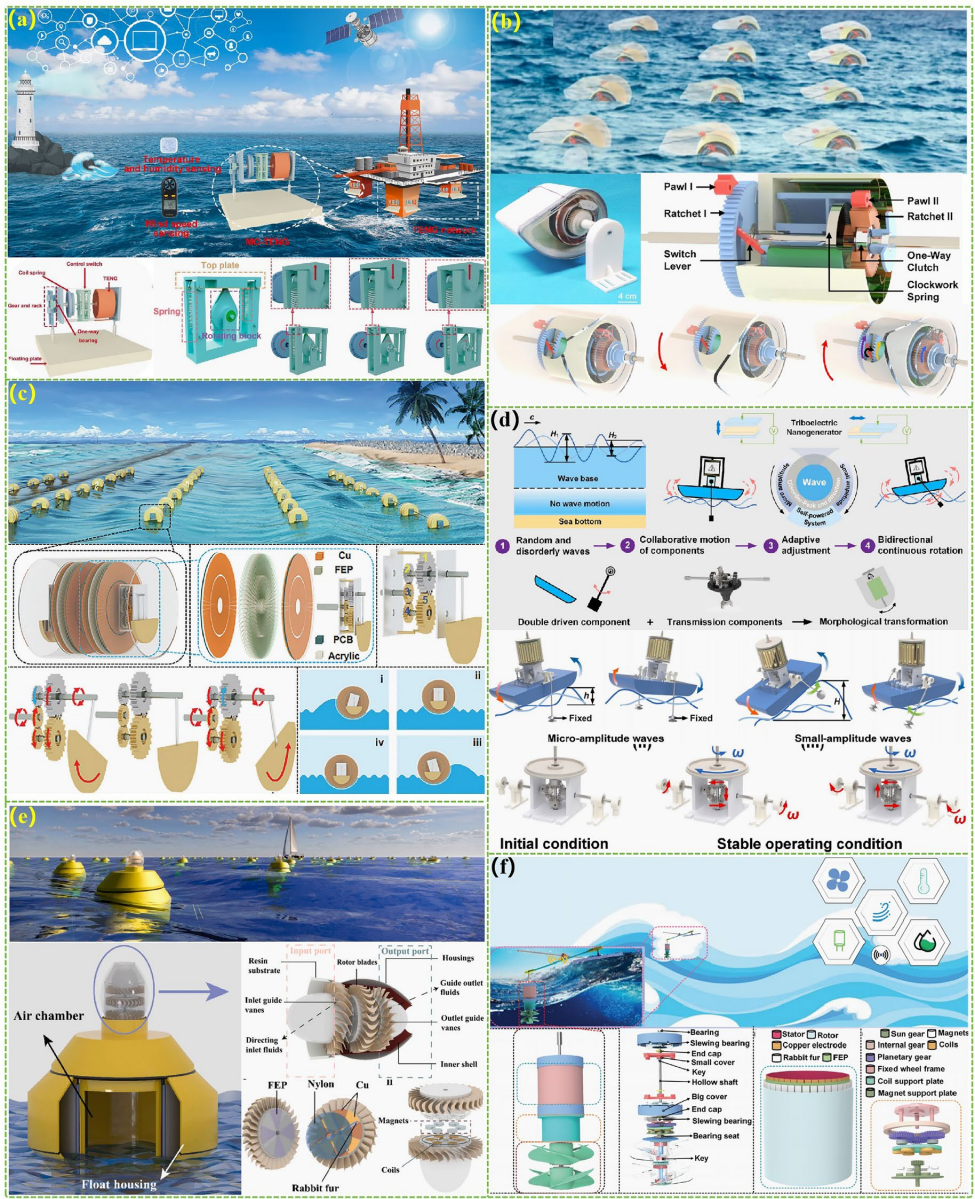
TENGs with the gear sets and turbines for the marine energy harvesting. **a** Rotatory TENG with simple mechanical control switch. Reproduced with permission from Ref. [189]. Copyright 2024, Springer Nature. **b** Rotating TENG with the integrated one-way bearings and energy storage springs. Reproduced with permission from Ref. [191]. Copyright 2022, American Chemical Society. **c** The gear set of rotation TENG that converts bidirectional swing into unidirectional rotation simultaneously. Reproduced with permission from Ref. [193]. Copyright 2023, Wiley–VCH. **d** The rotation TENG with differential gears and two unidirectional gears. Reproduced with permission from Ref. [195]. Copyright 2024, Elsevier. **e** Rotating hybrid generator based on pneumatic turbine. Reproduced with permission from Ref. [200]. Copyright 2024, Elsevier. **f** Rotating hybrid generator based on hydraulic turbine. Reproduced with permission from Ref. [201]. Copyright 2024, Wiley–VCH

A fixed wave energy harvesting device was designed based on a float lever, gear set, adaptive elastic rotating TENG, and rotating EMG [188]. Its working principle is that the float generates corresponding up and down vibration motion under wave drive, and amplifies the driving torque through a lever to transmit the corresponding wave power to the strip guide gears in the gear set. The strip gear will drive the rotating gear to generate corresponding rotational motion, thereby driving TENG to work. In order to adapt to the adaptive elastic rotation TENG, the gear set was utilized to convert the reciprocating motion of up and down vibration into unidirectional rotational motion through grouping driving principle, thereby ensuring the efficient and stable operation of the entire device. Based on the above research, Yang et al*.* designed a simple mechanical control switch using a spring structure to convert the irregular electrical energy output of the wave energy harvesting TENG into the stable electrical energy output [39]. Subsequently, the joystick swing of the one-way clutch made by the integrated escapement structure was driven by waves to generate corresponding one-way rotational motion (Fig. 13a) [189]. Then a part of the power was stored in the coil spring for subsequent continuous pushing of the adaptive elastic rotation TENG to achieve higher wave energy conversion efficiency while driving the adaptive elastic rotation TENG to operate. However, the widespread application of the above fixed structural design has been greatly limited. Therefore, a cylindrical TENG designed through a connecting rod structure was proposed, which utilizes the vertical reciprocating motion of a floating plate driven by waves to obtain corresponding rotational motion, and then drives the rotating TENG to convert the wave’s potential energy into the related electrical energy [190]. In order to avoid power loss caused by directional reciprocating rotational motion, a unidirectional bearing has been proposed to obtain unidirectional rotational motion. By using similar structures, the ratchet principle can be used to store wave energy in a spring. When the stored power reaches a specific value, it is then released to drive the TENG to rotate and convert water wave energy into electric energy (Fig. 13b) [191]. This practice enables the TENG to achieve stable electrical output without being affected by changes in external water wave parameters. Using a rotating pendulum and one-way bearings to temporarily store irregular wave power in a spring as well as combined with the centrifugal speed limiter, the stored power output was controlled to drive TENG for achieving stable electrical energy output. Based on ternary triboelectric materials and multilayer unit stacking, the output performance of the rotating TENG has been effectively improved, resulting in an average volume power density of 15.67 W m^−3^ for the wave energy mobile TENG [192]. However, the above simple oscillating to unidirectional rotation mechanical structure design results in half of the energy wasted. A gear group with a simpler structure that can simultaneously transform the bidirectional swing into unidirectional rotation, which consists of two unidirectional gears and three bidirectional gears (Fig. 13c) [193]. The specific operating principle is when the clockwise swing of the right unidirectional gear is used as the driving gear to actuate the whole gear group work, and when the counterclockwise swing of the left unidirectional gear to actuate the whole gear group work, so that this design can always ensure that the middle output of the two-way gear to keep clockwise rotation. This ingenious design enables the adaptive elastic TENG to achieve efficient utilization of wave power. On the basis of the above gear set structure design, Wang et al*.* proposed to continuously optimize the electrical output of the wave energy harvesting device by adding a variable speed gear structure and combining it with a hybrid generator [194]. A gear set composed of differential gears and two unidirectional gears was subsequently designed to simultaneously convert bidirectional oscillation into unidirectional rotational motion (Fig. 13d) [195]. Its working principle is that when a counterclockwise swing occurs, the left gear swings and drives the upper and lower parts of the differential gear to rotate relative to each other due to the idle action of the one-way gear and the right gear rotates in a passive state without affecting the rotation of the differential gear. When a clockwise swing occurs, the swing of the right gear will drive the operation of the differential gear, while the left gear is in a driven state. Due to the fact that differential gears can further increase the rotational speed of TENG, wave energy harvesting devices designed based on this gear will have higher electrical output. A rotating TENG with a mechanical adjuster was constructed using the principle of automatic winding of gear sets in mechanical watches [196]. It can achieve stable energy harvesting, storage, and release under random driving conditions, making it very suitable for wave energy harvesting with fluctuating power output characteristics. An intelligent energy harvesting hybrid generator with adaptive driving condition fluctuations and output regulation functions has been proposed based on mechanical principles [197]. The designed gravity-driven roller and seesaw structure have particularly sensitive corresponding functions to low-frequency and irregular mechanical movements. It can convert the bidirectional swing of the seesaw into high-speed rotational kinetic energy of the permanent disk through variable speed gears and then drive the hybrid generator to achieve efficient wave energy conversion. A mechanical frequency modulator was designed to increase the electric output of wave energy harvesting TENG by integrating the aforementioned differential gear based swing to rotation gear set, an automatic switching device that adjusts the number of rotations of the main spring using a composite gear set, and a flywheel structure for storing excessive power [198]. Among them, the rotating TENG was connected to the mainspring through gears to achieve automatic adjustment of TENG output function without relying on the flywheel. It can effectively reduce the moment of inertia of the rotating TENG and thus minimize the energy required for the entire device to start. In the field of turbine research, a wave energy harvesting TENG driven by wave motion airflow was designed using an airflow turbine and non-contact rotating TENG [199]. After optimizing the structural design of the pneumatic device through experimental simulation based on the principles of fluid mechanics and theoretical wave models, this TENG can achieve an short-circuit current of 55.45 μA, an electrical power of 5.28 mW and an electrical volume power density of 114.8 W m^−3^. Subsequently, by integrating the ring array EMG design, the entire generator system was able to obtain stable and sustained power output with the maximal volume power density of 362.23 W m^−3^ even under extremely low-frequency driving conditions (Fig. 13e) [200]. Meanwhile, the triboelectric-electromagnetic hybrid generator developed through the design and optimization of the dual turbine collaborative structure and an average volume power density of 541.7 W m^−3^ was achieved by the rotatory TENG (Fig. 13f) [201]. This study has played a significant role in promoting the future large-scale application of TENG.

#### Omnidirection

**Fig. 14:**
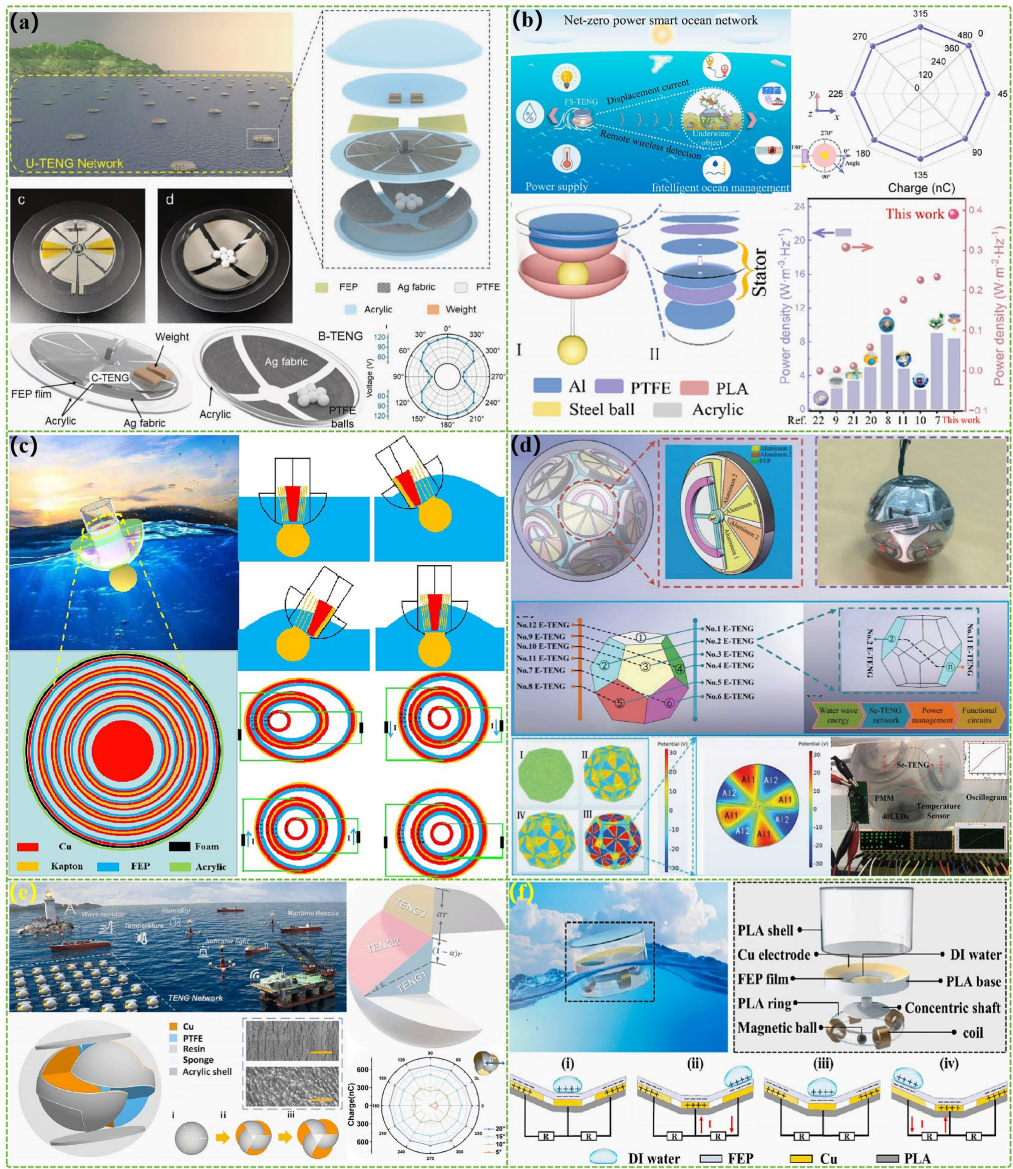
Omnidirectional marine energy harvesting TENGs. **a** A wave energy TENG constructed by integrating the flexible sliding structure and ellipsoidal structure. Reproduced with permission from Ref. [205]. Copyright 2023, Wiley–VCH. **b** A fully symmetrical TENG with the simplified double-layer electrode. Reproduced with permission from Ref. [207]. Copyright 2023, Elsevier. **c** Active resonance omnidirectional wave energy harvesting TENG. Reproduced with permission from Ref. [130]. Copyright 2021, Elsevier. **d** A spherical TENG that integrates 12 eccentric pendulum units. Reproduced with permission from Ref. [212]. Copyright 2022, Wiley–VCH. **e** A dual-mode spherical TENG with 3D fractal structure. Reproduced with permission from Ref. [213]. Copyright 2024, Elsevier. **f** The omnidirectional wave energy harvesting TENG through circular electrode pairs. Reproduced with permission from Ref. [215]. Copyright 2023, Elsevier

Due to the unpredictable direction of various renewable energy in the ocean, such as ocean currents undersea and wave energy on the water surface, TENG, which previously operated in a single direction, will find it difficult to maximize its effectiveness in real marine environments [33]. To address this issue, researchers first thought of exploring the feasibility of omnidirectional ocean renewable energy harvesting through a simple system integration approach by installing multiple TENG units at different locations. A symmetrical bow-shaped TENG is installed with corresponding units in four directions and achieves multidirectional wave energy harvesting through a contact separation working mode under the impact of freely rolling steel balls in the middle [202]. Subsequently, the S-shaped flexible TENG based on flexible thin film structure was also designed as a dodecahedron integrated structure, utilizing the impact generated by the internal steel balls under wave excitation to achieve corresponding electrical energy conversion [203]. According to research on the gravitational potential energy and horizontal kinetic energy of wave energy, it has been found that the gravitational potential energy of wave energy is significantly higher than its kinetic energy [124]. A hybrid TENG has been proposed for the collecting of omnidirectional water wave energy, which harvests wave gravitational potential energy by installing a multilayer structural TENG in the vertical direction and harvests wave energy kinetic energy by installing a petal electrode rolling TENG at the bottom [204]. However, due to the small contact area of a single ball point, its utilization of horizontal wave power was extremely low. Therefore, a wave energy TENG constructed by integrating a flexible sliding structure TENG and an ellipsoidal structure optimization based on optimizing the contact area of multiple small balls further optimizes the space efficiency and electric output of the entire device (Fig. 14a) [205]. However, due to the problem of working dead corners, it is still difficult to achieve omnidirectional wave energy harvesting in the above-mentioned designs. The rolling TENG constructed by designing corresponding circular array electrodes in the bread ring structure and combining with rolling balls was designed by the omnidirectional degrees of freedom of the rolling balls inside the bread ring to harvest omnidirectional water wave energy [206]. Besides, a structurally simple and fully symmetrical TENG was designed by utilizing the arbitrary degrees of freedom in the three-dimensional space of a pendulum ball and the characteristic of a simplified double-layer electrode structure with arbitrary orientation (Fig. 14b) [207]. Based on a completely symmetrical structural design, this TENG has high adaptability and can achieve stable output under any water wave drive. In order to boost the electrical output of wave energy harvesting TENG in the different direction, Liang et al. integrated six springs and steel balls assisted multilayer TENG into a spherical TENG device by fully utilizing the damping characteristics of springs and the high electrical output of multimeasurement structure TENG to collect wave energy in six degrees of freedom directions in three-dimensional space [208]. The experimental results show that under the driving of simulated water waves at 1.0 Hz and 90° vertically, this spherical TENG can achieve an output short-circuit current of 80 μA and a peak power of 8.5 mW. By studying the relation between the electrical output and angle of this spherical TENG, it was found that it can have good energy converting efficiency for water wave energy in different directions. A flower-shaped TENG was designed using a similar structural design, which was consisted of six multilayer TENGs with folded structures and two flower core TENGs with vertical contact separation modes [209, 210]. The flower core TENG was driven by a top heavy object to harvest one degree of vertical wave gravitational potential energy, while the petal TENG was driven by an integrated cube that integrates the flower core TENG to harvest two degrees of horizontal and three degrees of rotational kinetic energy. Due to the excellent elasticity of the origami structure TENG, this design eliminates the need for spring components. In addition, a circular wave energy harvesting device is designed using 12 fan ring TENG units spliced together [211]. Thanks to its denser unit integration design, it has better responsiveness to wave energy in different directions. By utilizing the omnidirectional degrees of freedom of both pendulum and single pendulum structures as well as the active resonance effect coupled by their damping effects, a flexible circular array TENG was designed to achieve effective harvesting of waves with the omnidirection and full frequency bands (Fig. 14c) [130]. For the research of sliding TENG, Qu et al*.* developed a spherical TENG that integrates 12 eccentric pendulum TENG units [212]. Based on the inspiration from the burr puzzle structure, a dual-mode spherical TENG with 3D fractal structure was designed by using the three module multiphase coupling principle of ingenious 3D space geometric structure design. Relied on the collaborative coupling characteristics between modules, the output characteristics with the contact separation and the lateral sliding modes were obtained (Fig. 14e) [213]. By fully utilizing the high electrical output and high space efficiency provided by sliding friction, the dual-mode TENG achieved an average volume power density of 1.1 W m^−3^ and a maximal volume power density of 8.3 W m^−3^. Regarding the research on liquid–solid TENG, a double-layer disk-shaped liquid–solid TENG utilizing the variation of upper and lower contact areas has been designed to achieve omnidirectional wave energy collecting by the omnidirectional degrees of freedom of the liquid [214]. Due to the ultra-high surface efficiency of the disk structure, it can achieve a transfer charge of 1.263 μC under the drive of a seesaw swing with the frequency of 0.5 Hz and the amplitude of 30°. A TENG for harvesting omnidirectional wave energy was designed by preparing concentric circular electrode pairs on a satellite dish with oblique angles and utilizing the omnidirectional gravity driven characteristics of liquids (Fig. 14f) [215]. Compared to other solid–liquid TENGs, this design further improves the controllability of liquid movement in any direction.

## Engineering Study

As the ultimate goal of any scientific and technological development, the engineering research conducted prior to it has a crucial impact on accelerating the large-scale development of the entire technology. Engineering application study is different from previous related theoretical research. It mainly considers various problems that may be faced in the process of large-scale application and studies feasible solutions. It has many requirements such as strong targeting, high economy, and excellent stability. The engineering study on TENG for marine energy harvesting can be mainly divided into two aspects: practical optimization and in situ utilization.

### Practical Optimization

Although TENG has unique technological advantages in the development and research of marine energy, it inevitably encounters various problems in practical applications. The very important research directions are topology design and pilot-scale testing. The pilot-scale testing plays a decisive role in meeting various requirements for commercial development of TENG, while the topology design has a vital impact on the electrical output, cost-effectiveness, and environmental protection of the entire TENG scale application process.

#### Pilot Testing

**Fig. 15:**
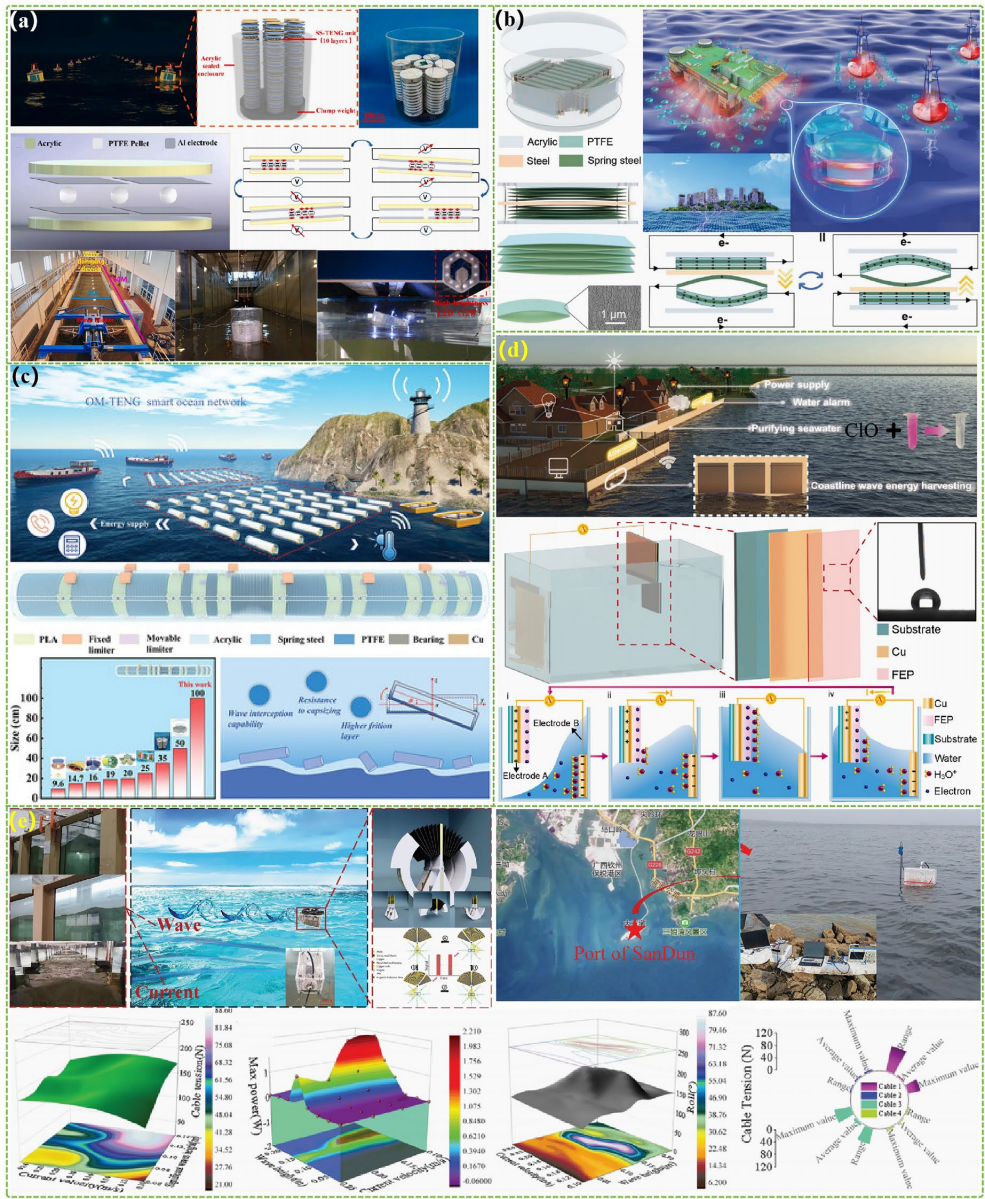
The pilot testing of TENG for the marine energy harvesting. **a** A sandwich rolling TENG device with the 70 units. Reproduced with permission from Ref. [216]. Copyright 2021, Elsevier. **b** The large wave energy harvesting device with a diameter of up to 0.5 m based on the double-bow structure of the TENG unit. Reproduced with permission from Ref. [217]. Copyright 2022, Wiley–VCH. **c** A wave energy harvesting TENG with a size of 1 m. Reproduced with permission from Ref. [218]. Copyright 2024, Wiley–VCH. **d** The instantaneous contact liquid–solid TENG with the electrode area of 0.12 m^2^. Reproduced with permission from Ref. [219]. Copyright 2024, Elsevier. **e** A coaxial hybrid energy generator with the large size. Reproduced with permission from Ref. [220]. Copyright 2024, Wiley–VCH

To test the size effect of TENG in the harvesting of marine energy, a spherical rolling TENG grid based on a 4 × 4 array integration mode was proposed [55]. Relevant studies have found that the integration mode of multiple elements can further optimize the synergistic effect of each TENG element to achieve more efficient wave energy harvesting. Therefore, this study also proves that the scheme based on TENG grid structure design has high feasibility in large-scale harvesting of marine energy. A sandwich rolling TENG unit with a multipoint contact structure design and high electrical output achieved a volume power density output of 34.65 W m^−3^ due to a larger contact area and space efficiency, which is more than twice as high as the previous TENG with the same structure (Fig. 15a) [216]. Thanks to its simple structure, it has the advantages of easy stacking and parallel integration. The experimental surface shows that the electrical performance improves linearly with the increase of the number of parallel units within 70. This feature laid a solid foundation for the scale-up of the entire TENG pilot scale. Therefore, a large-sized wave energy harvesting TENG device was constructed by arranging 70 TENG units in the most densely packed six sided plane and 10 units in parallel in the longitudinal direction, which exhibited excellent performance in real marine environments. Subsequently, researchers used three-dimensional potential flow theory and boundary element method to establish a numerical model of this large-sized TENG. The influence of mooring configuration, incident wave height, and frequency on its hydrodynamic characteristics and electrical output was studied through numerical simulation analysis and experimental verification, providing important theoretical basis for continuous improvement and optimization in future. Feng et al*.* designed a large wave energy harvesting TENG with a diameter of up to 0.5 m by the double-bow structure of the TENG unit, in which the double-bow structure enables the TENG unit to obtain good contact separation characteristics and thus ensure its excellent electrical output (Fig. 15b) [217]. By adding steel plates to the center of each TENG unit based on the operative conditions, the TENG unit can obtain good electrical output even at a small deflection Angle of 7 degrees. As a result, this half-meter TENG can achieve excellent electrical output in the frequency range of 0.1 to 2 Hz, with charge transfer up to 67.2 μC. Due to the excellent stability and mobility of the ellipsoid shell and the way the TENG units are integrated in different directions, the related design can be easily adapted to complex marine environments. On this basis, a wave energy harvesting TENG with a size of up to one meter has been designed (Fig. 15c) [218]. It contains multiple sliders that can move freely along the central axis of the circular tube with low friction. Under the drive of waves, these sliders use their own reciprocating motion to drive the densely arranged double-bow TENG units between them and achieve the electromechanical conversion of wave energy. To continuously reduce the large fixed space occupied by a single TENG, all TENG units were designed to maximize the use of common space to complete the contact separation process and greatly improve the space efficiency of the entire device. The use of lantern-shaped spring steel sheets further increases the installation quantity of TENG units, thereby increasing the contact area density of TENG to the highest level of current wave energy harvesting TENG research and reaching 1.76 cm^−1^. In addition, each multiunit integrated TENG structure was connected at both ends using sliders to prevent the collapse of the entire series structure and avoid energy loss caused by asynchronous operation between each TENG unit. The added segmented limiter can accurately control the motion amplitude of all sliders, thereby controlling the center of gravity change of the entire device within a safe orientation to prevent the problem of working condition failure caused by large waves. At the same time, theoretical analysis, multiphysics field parameter simulation, sensor testing, and control group experimental research have verified that this TENG scheme has good anti overturning ability in complex waves. After diversified parameter optimization design and experimental testing, the designed and installed giant TENG achieved a highest volume power density of 28.9 W m^−3^ and a charge transfer amount of 60.82 μC per cycle. In the research of liquid–solid TENG, the method of designing liquid–solid TENG through instantaneous contact has been developed to solve the problem of adverse effects on the electrical output of liquid–solid TENG caused by ion adsorption in the double layer (Fig. 15d) [219]. The related design has expanded the liquid used in liquid–solid TENG from deionized water to many other liquids such as lake water and seawater, greatly improving the application range of liquid–solid TENG. At the same time, a large-sized liquid–solid TENG with an electrode area of up to 0.12 m^2^ was installed, which can achieve high transfer charge output of 2.5 μC and high instantaneous current output of 2 mA under saltwater testing conditions. More importantly, the self-powered electrolytic saltwater device constructed by it can achieve a degradation rate of 98.83% for the organic dye RhB within 6 h. In the research of hybrid generators, a coaxial hybrid energy generator with a large size has been developed for testing in real ocean wave environments (Fig. 15e) [220]. Combined with the relevant experimental tests of the wave making pool can be found: 1) the designed hybrid generator has a lower electrical output under irregular wave driving than under regular wave driving; 2) the variation pattern of electrical output under actual ocean wave testing is basically consistent with laboratory research, but the output size is slightly lower than laboratory testing; and 3) the electrical output of the designed hybrid generator is closely related to its draft depth and ocean currents have a suppressive effect on the electrical output.

#### Topology Design

**Fig. 16:**
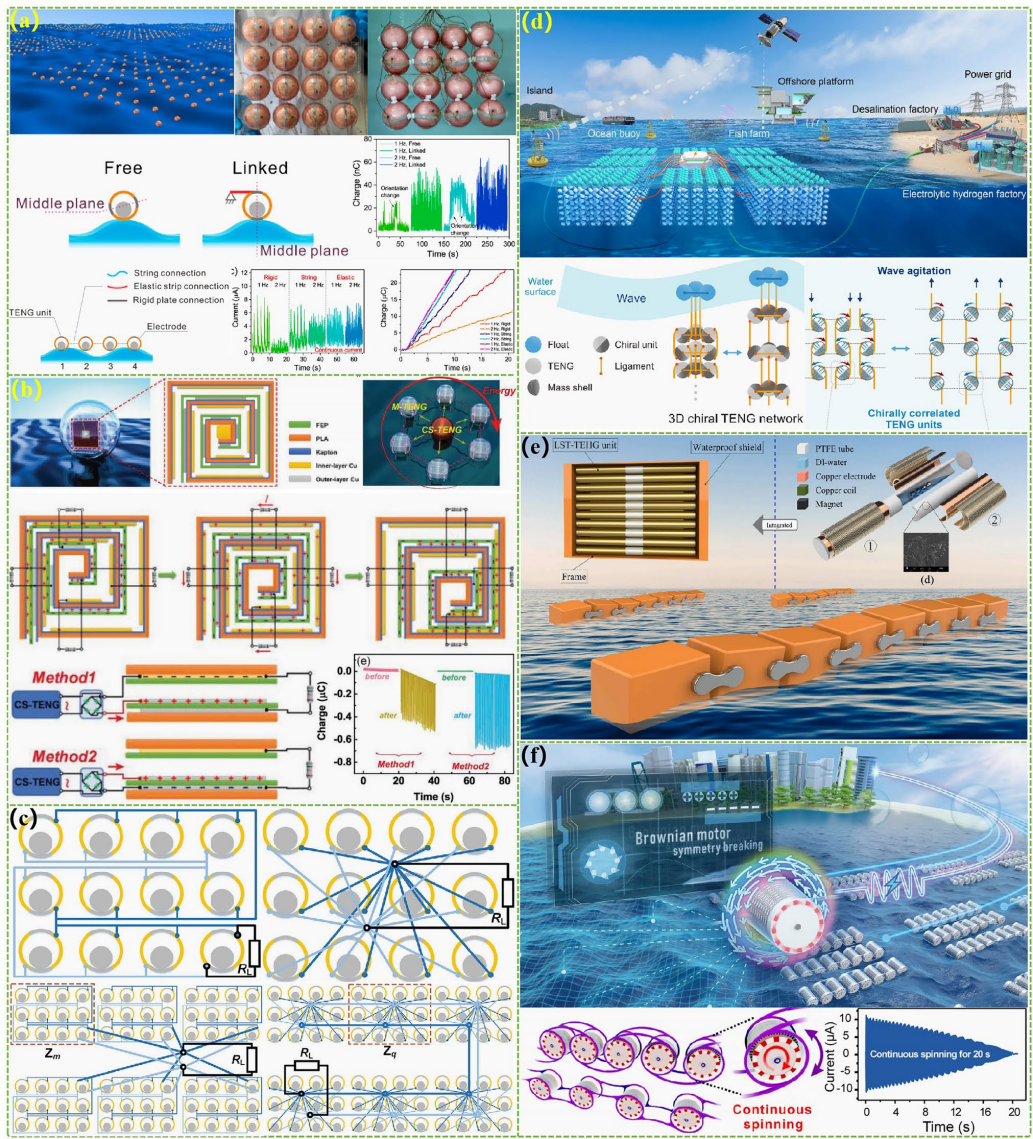
Topological structure researches of TENG network. **a** The research of the TENG connection method. Reproduced with permission from Ref. [55]. Copyright 2018, American Chemical Society. **b** The design of TENG network with the external charge excitation. Reproduced with permission from Ref. [91]. Copyright 2019, Wiley–VCH. **c** The TENG network of four network integrating methods. Reproduced with permission from Ref. [224]. Copyright 2020, Elsevier. **d** A TENG network with a three-dimensional chiral structure. Reproduced with permission from Ref. [225]. Copyright 2023, The Royal Society of Chemistry. **e** A hinged rotating array topology design with the circular tube TENG unit. Reproduced with permission from Ref. [165]. Copyright 2024, Elsevier. **f** The TENG network based on directed rotation of Brownian motor. Reproduced with permission from Ref. [27]. Copyright 2023, The Royal Society of Chemistry

Regarding the topology research of TENG ocean renewable energy harvesting, initially, by drawing on traditional wave energy research experience, researchers proposed to construct a cavity along the coast, with its opening surface placed below the water surface, and then use wave motion to generate corresponding airflow to drive TENG to work, in order to harvest corresponding wave energy [221, 222]. However, its reliance on coastal design severely limits its application scope. More importantly, compared to mature research on EMG in this area, this design has no advantages and has not fully considered the advantages of TENG. Inspired by the excellent hydraulic characteristics of jellyfish, Chen et al*.* proposed a biomimetic TENG to harvest wave energy, which consists of a float, spring, TENG unit, and a weight block connected in series [223]. Its work principle is to drive the contact separation TENG by the tension between the buoyancy generated by the float and the gravity of the weight under wave driving. However, this topology design has not been continuously studied due to its complex structural composition and low electrical output. Based on the spherical structure TENG unit, Wang proposed using a network designed topology to achieve a large-scale wave energy harvesting scheme, fully leveraging the advantages of TENG in constructing floating wave energy harvesting devices [22]. Through in-depth research on the TENG connection method, it was found that the connected TENG network units have higher electrical output compared to free TENG units as well as the elastic connection method has more sustained and stable electrical output than the rigid and string connection methods (Fig. 16a) [55]. Subsequently, based on the principle of external charge excitation, a topology structure was designed to greatly boost the electric output of the entire TENG network by using one TENG unit as a charge pump to charge other main TENG units (Fig. 16b) [91]. Liu et al*.* designed a planar cable obtained by the integrating spring steel sheets and three-layer polymer films as the wires and ropes for connecting the various units of the TENG network [42]. This type of cable uses spring steel plates to have a triple function as a structural skeleton, wire, and electrode. This multilayer structure design not only avoids the clothing effect of seawater, but also has the function of reducing the influence of ions in seawater on electrical energy transmission through electrostatic induction. It can also further increase the power output of this TENG network by constructing corresponding liquid–solid TENG through PTFE on the outside. In addition, four network integrating methods have been proposed for the specific topology design of large TENG networks (Fig. 16c) [224]. Through theoretical calculations, it has been found that the second method has significant advantages in power output and efficiency. Through systematic research, it was also found that reducing the resistance of wires and synchronizing the output phase of each TENG unit have a crucial impact on improving the electrical output of the entire network. A TENG network with a three-dimensional chiral structure was proposed based on the structure derived from mechanical metamaterials (Fig. 16d) [225]. Unlike the previous rigid network structure connection method, this new TENG network uses a distributed structure design to enable chiral connections between unbalanced TENG units. This mechanical chirality similar to metamaterials enables the entire TENG network to flexibly and elastically harvest water wave power in water. More importantly, this network design can be diversified in scale and depth installation according to relevant needs to achieve comprehensive ocean renewable energy harvesting, and the designed power management integrated energy harvesting system can increase the amount of energy stored by more than 300 times. Meanwhile, to enable the shock wave energy harvesting TENG with a single working direction to adapt to the random changes in the propagation direction of deep sea waves, a hinged rotating array topology design with the circular tube TENG unit has also been proposed (Fig. 16e) [164]. However, the complexity of this design and the effective surface area of the circular tube make it difficult for the relevant design to be applied on a large scale. A topology design scheme for a sea python structure has been proposed based on the motion TENG of the relevant TENG unit seesaw type [127]. It uses ropes or rotating hinges to connect the TENG units from both ends of the working direction to form a chain like array similar to a sea python structure. This design anchors one end in the sea, and then the TENG unit converts wave energy into electrical energy through a seesaw motion under the continuous impact of waves. Due to the unidirectional working mode, this topology design is only suitable for nearshore waters where the direction of wave propagation is basically fixed [124]. To address the issues of poor maintainability and system responsibility in previous topology research on TENG network design, a topology design scheme was proposed that integrates a large number of pendulum wave energy harvesting TENG modules into the vessel. The parameters of the relevant vessel and TENG module can be designed based on the hydrological conditions of specific sea areas to fully utilize principles such as resonance effects to maximize the energy converting efficiency of the entire wave energy harvesting vessel. Finally, based on the principle of directed rotation of Brownian motors, a continuous rotating TENG network was designed to harvest irregular wave energy through the methods of double symmetry breaking and inertia flywheel power buffering (Fig. 16f) [27]. The ratchet effect of asymmetric and unidirectional bearings integrated with non-mirror networks enables the designed TENG to convert random wave forces into its own unidirectional rotational motion, while the power buffer device with an inertial flywheel can be used to buffer and accumulate irregular wave forces for long-term rotation. The unidirectional rotational characteristics not only solve the problem of phase mismatch in TENG operation, but also achieve efficient utilization of new wave forces at any time to obtain corresponding rotational acceleration. Therefore, unlike the intermittent wave energy harvesting TENG proposed in previous related studies, this design can effectively harvest wave power to obtain continuous and long-term intermittent outputs, thereby greatly enhancing the overall electrical output. Through water wave testing, it was found that the low-friction and high-output TENG obtained by using radial grid structure and soft contact obtained a high average volume power density output of 4.69 W m^−3^. This work effectively addresses the adverse effects of water wave randomness on TENG energy generation and providing an important solution for TENG to scale up the harvesting of irregular marine energy.

### In Situ Utilization

For the in situ utilization of marine energy, on the one hand, obtaining various undersea information plays a vital role in ensuring the smooth progress of related activities throughout the entire ocean development and utilization process. Currently, undersea monitoring almost entirely relies on various undersea sensors. Undersea sensors based on battery energy storage devices cannot work for a long time due to limited electrical energy, and the undersea environment cannot provide electricity through solar, wind, and wave energy. Therefore, it will be urgent to develop efficient undersea energy harvesting devices. On the other hand, with the continuous promotion of the dual carbon strategy, it is also of great significance to use marine energy as a power source combined with electrochemical technology to produce clean fuels and fresh water *in situ* as well as reduce marine environmental pollution.

#### Undersea Energy

**Fig. 17:**
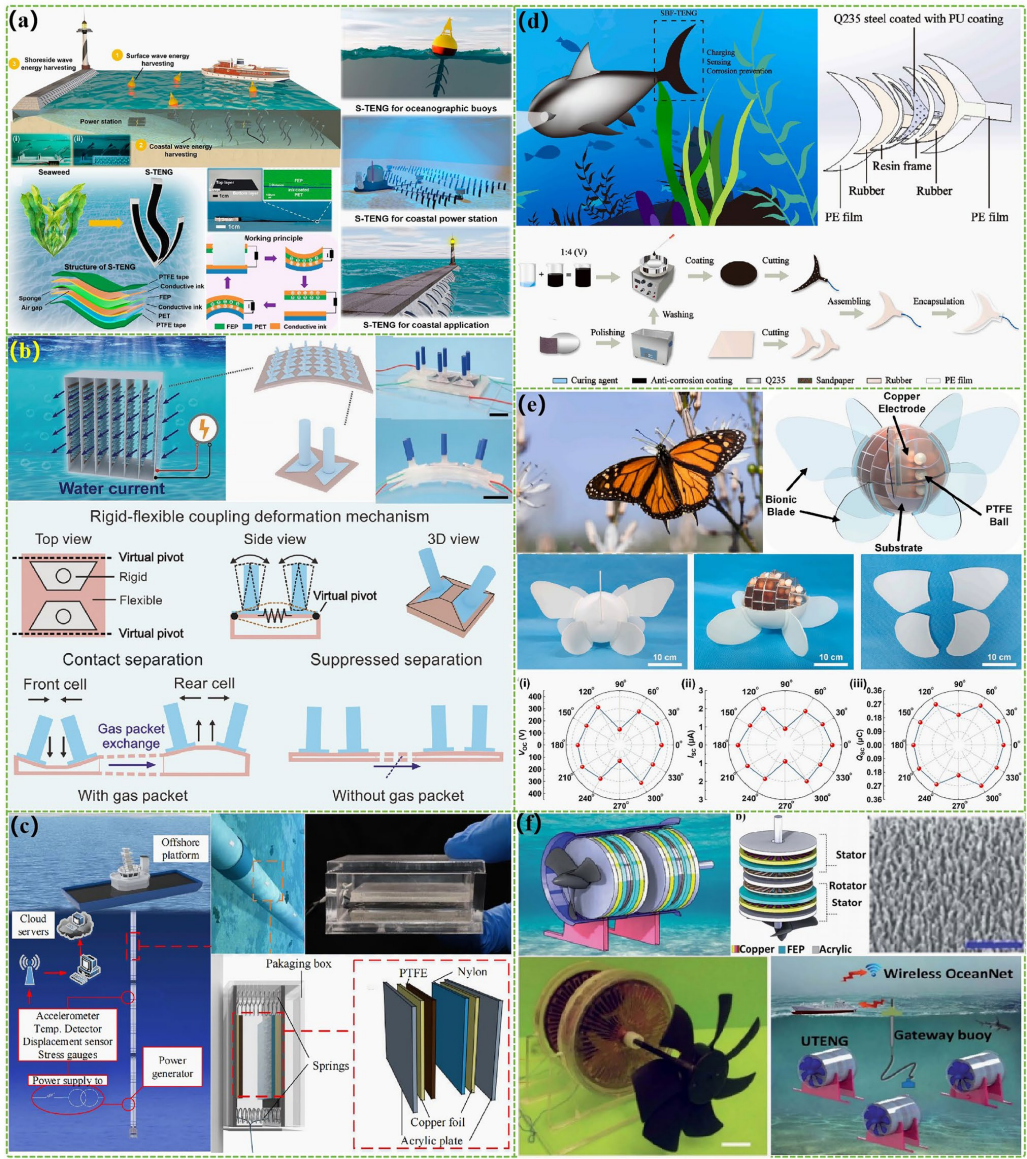
Researches of undersea energy harvesting TENG. **a** An undersea energy harvesting TENG with biomimetic seagrass structure. Reproduced with permission from Ref. [228] Copyright 2021, American Chemical Society. **b** An undersea eddy current energy harvesting TENG. Reproduced with permission from Ref. [229] Copyright 2022, Wiley–VCH. **c** Undersea pipeline vibration energy harvesting TENG. Reproduced with permission from Ref. [230] Copyright 2019, MDPI AG. **d** Undersea energy harvesting TENG with biomimetic fish fin structure. Reproduced with permission from Ref. [233] Copyright 2023, Elsevier. **e** An undersea energy harvesting device consisting of biomimetic butterfly wings and TENG units. Reproduced with permission from Ref. [234] Copyright 2022, Elsevier. **f** An underwater TENG to obtain electricity from the power of currents. Reproduced with permission from Ref. [235] Copyright 2022, The Royal Society of Chemistry

To harvest the energy of ultrasound waves widely distributed undersea in the ocean, a TENG for undersea operation was developed based on the vibratory ball [226]. The TENG prototype, which was optimized by studying the relevant design parameters of TENG, achieved a high current output of 0.12 A and an energy conversion efficiency of 13.1% under 80 kHz ultrasonic drive. A flexible TENG was fabricated by combining conductive ink as an electrode with triboelectric materials, which can obtain corresponding mechanical energy through simple swinging [227]. On this basis, a flag shaped undersea TENG that can be harvested by ocean currents was designed by utilizing the Karman vortex street effect. The relevant experiments show that it can operate under water flow conditions greater than 0.0133 m s^−1^ and achieve a power output of 52.3 μW through a device integrated with 6 TENG units under water flow conditions of 0.461 m s^−1^. Compared to PENG and EMG, this TENG device has significant advantages in harvesting low velocity ocean current energy. In addition, inspired by the structure of undersea seagrass, a sea maneuvering TENG was designed by combining this flexible TENG with an elastic film (Fig. 17a) [228]. Compared to flag-shaped TENGs, this TENG has higher electrical output thanks to its elastic structure that provides efficient contact. Deng et al*.* developed an undersea eddy current energy harvesting TENG, which is easy to integrate and install through a thin flexible layer using ultra-thin structural design (Fig. 17b) [229]. It also has a function as a structured triboelectric electric surface. This TENG can achieve efficient operation under high water pressure in deep water conditions, which is benefiting from the additional gas exchange structure and the rigid flexible coupling deformation mechanism. Research has found that optimizing the composition of TENG arrays on structured triboelectric surfaces can significantly enhance the electrical output of TENG and convert unidirectional water flow energy into high-frequency electrical energy output. The implementation test shows that a 4.5-mm-thick TENG can generate a charge output of 1.59 μC s^−1^ and a high-frequency electrical output of 57 Hz when driven in a water flow of 2.43 m s^−1^. The corresponding power density was more than 100 times that of other TENG devices that harvest ocean currents. In the research of undersea TENG for structural applications, an efficient vibration energy harvester based on contact separation mode TENG has been developed, which can be installed on undersea pipelines to convert the vibration energy of pipelines under water flow excitation into electrical energy (Fig. 17c) [230]. Therefore, it can be applied to the design of self-powered monitoring systems for various undersea pipelines. As the most common undersea animal, fish's own structure provides many innovative inspirations for the design of undersea TENGs. Design a biomimetic undersea TENG by utilizing the characteristics of fish fins swinging up and down in undersea waves and currents [231]. The TENG was endowed with the effective contact and high space efficiency through structural design, and its volume power density can up to 444 W m^−3^. During this period, inspired by the swaying of the fish tail in the water flow, various biomimetic fish tail structures of undersea TENGs were designed. The electrical output of undersea TENG is largely improved by utilizing methods such as structural optimization, fluid dynamics self-defense, and triboelectric material optimization (Fig. 17d) [43, 232, 233]. In addition, an undersea energy harvesting device consisting of biomimetic butterfly wings and TENG units was designed based on the wings of butterflies (Fig. 17e) [234]. Based on butterfly wing array design, it can harvest water flow energy from different directions undersea. By using fluid dynamics simulation to calculate the shape and quantity of butterfly wings, the optimal structural parameters were obtained. Based on the above design parameters, the corresponding prototypes was fabricated. The undersea energy harvesting installed in the relevant tests can achieve sensitive response to water flow in multiple directions, and obtain an output current of 2.9 μA, a transferred charges of 0.31 μC and an output voltage of 400 V driven by water flow at a frequency of 1.25 Hz. In the research of rotating TENG, a parallel stacking design and turbine design based on ordinary rotating TENG have been developed for undersea ocean current energy harvesting TENG (Fig. 17f) [235]. It can obtain a power output of 200 mW undersea to drive stable operation of ocean sensors such as pH and turbidity, thereby achieving self-powering function of undersea ocean IoT network nodes. For the research of undersea hybrid generators, a hybrid generator with a rotating gyroscope structure was designed by wrapping a layer of triboelectric material around a conical magnet and combining corresponding electrodes and coils to efficiently harvest low-frequency and irregular undersea vibration energy [151]. A self-powered undersea tracking device was constructed by integrating it into the body of an undersea vehicle and combining it with a GPS module. The relevant design scheme was effectively validated in the Yellow Sea using an autonomous undersea vehicle. An efficient ocean current energy harvesting device was constructed using a hybrid generator with a turbine and rotating structure [236]. The device can be powered by a lithium battery as an energy storage device to drive a signal processing module to detect its own output signal and obtain corresponding ocean current velocity information. Combined with a wireless communication module, a fully self-powered wireless sensor node for the ocean Internet of Things was constructed.

#### Electrochemistry

**Fig. 18:**
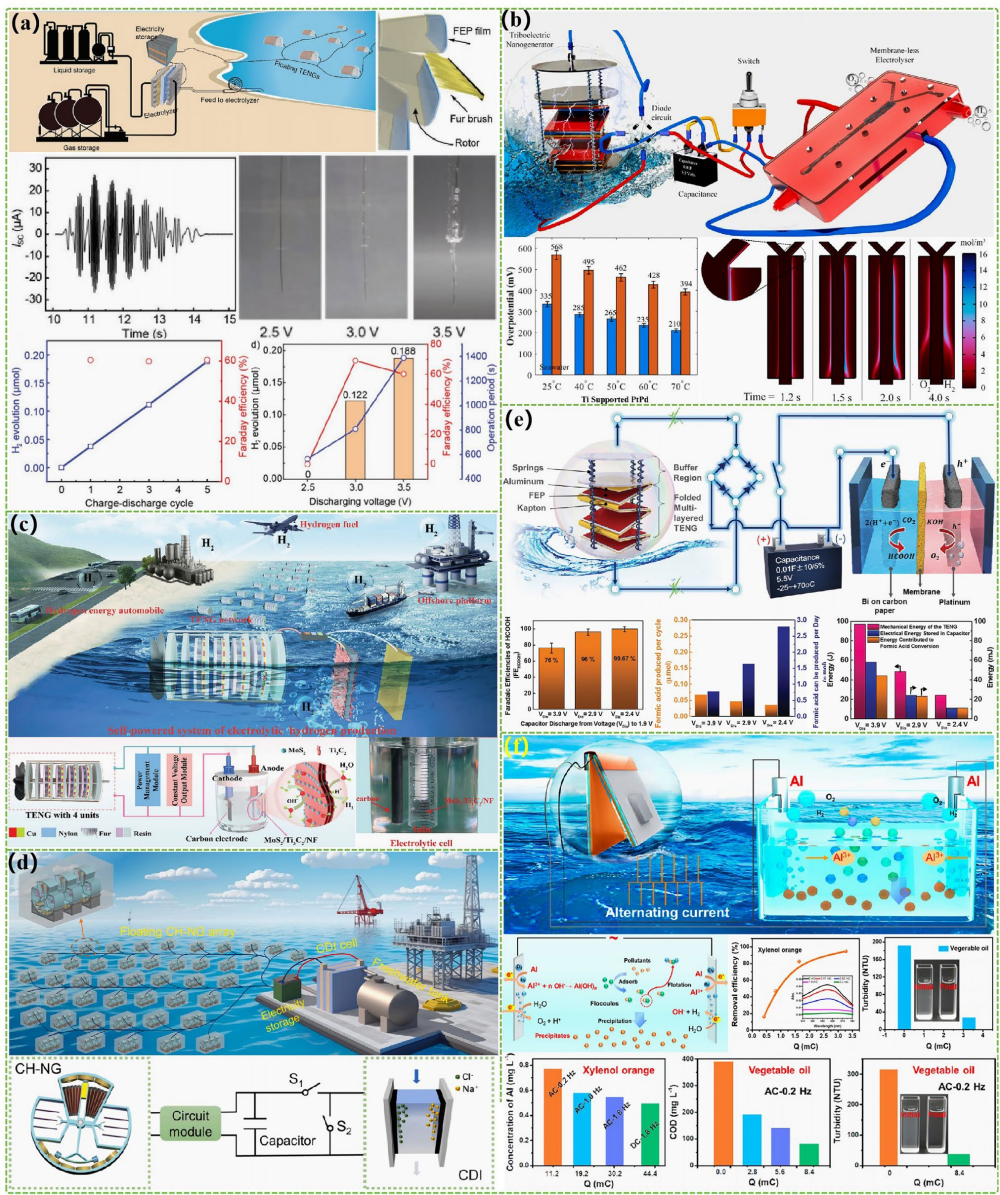
Self-powered electrochemical system relied on marine energy harvesting TENG. **a** Self-powered electrochemical hydrogen production system by pendulum rotating high-output TENG. Reproduced with permission from Ref. [244]. Copyright 2022, Wiley–VCH. **b** A simple water electrolytic device with the membrane free electrochemical cell reactor and TENG. Reproduced with permission from Ref. [245]. Copyright 2024, Elsevier. **c** High-speed hydrogen production system with MoS_2_/Ti_3_C_2_ catalyst and power supply. Reproduced with permission from Ref. [28] Copyright 2024, Wiley–VCH. **d** A self-powered desalination device with low power consumption. Reproduced with permission from Ref. [247]. Copyright 2024, Elsevier. **e** Electrochemical synthesis of formic acid system with wave harvesting. Reproduced with permission from Ref. [248]. Copyright 2020, The Royal Society of Chemistry. **f** Electric flocculation sedimentation system driven by wave energy. Reproduced with permission from Ref. [249]. Copyright 2022, American Chemical Society

Due to the unique environmental characteristics of the ocean, the direct transmission of electricity, which is obtained through marine energy harvesting, back to land use has both economic and technical challenges. Based on the abundant water resources in the ocean, converting the harvested electric energy into high-value hydrogen fuel will have a very broad application value. However, due to the low current and high voltage output features of TENG, the demand for low voltage and high current for hydrogen production by electrolysis is contradictory. In the initial stage of the research on hydrogen production from water electrolysis based on TENG, the rotating TENG with high current output was adopted by the related researches. At the same time, based on the solar photoelectric technology, the high voltage feature of the TENG can improve the voltage of the entire catalytic electrode to improve the hydrogen production rate [44, 237–239]. Compared with the single photoelectric hydrogen production, the generation rate after the integrated TENG can be improved by 50%. With the continuous improvement of TENG electrical output and the rapid development of corresponding power management technology for marine energy harvesting. Self-powered water electrolysis hydrogen production technology based entirely on marine energy harvesting TENG has been rapidly developed [240]. A rotating TENG was proposed to collect the water flow energy and store the corresponding energy to the capacitor [241]. When the stored energy reaches a certain amount, the water electrolysis device is combined to achieve the corresponding hydrogen energy acquisition. However, this kind of design does not have any practical value due to the problem of independent system composition and intermittent work. Therefore, based on the rotating TENG driven by water flow, an efficient self-powered hydrogen production system through half-wave rectification was designed [242]. The designed half-wave rectifier circuit can realize DC energy output with high voltage and low power loss, which enables the rotating TENG to achieve 90 μA and 100 V current output under 140 rpm driving conditions. Combined with the prepared electrochemical catalyst with excellent hydrogen evolution performance, the entire system can achieve a hydrogen production rate of 12.32 μL min^−1^ and a hydrogen conversion efficiency of 2.38%. To cope with the effect of marine corrosion on the performance of TENG, a composite electrode with good corrosion resistance was developed from conductive carbon black and polydimethylsiloxane, and the TENG prepared based on this material can be directly used for the harvesting of marine energy [243]. A self-powered seawater hydrogen decomposition system was constructed by harvesting simulated water wave energy to verify the feasibility of the relevant design. On the basis of increasing the contact area of the TENG by using the gear structure electrode design and reducing the friction force of the brush structure, a high electrical output rotating TENG was designed through combining the pendulum structure (Fig. 18a) [244]. A self-powered blue hydrogen production system was constructed through array design, energy storage device and electrolytic cell simulating seawater electrolysis. It can achieve the production rate of 814.8 μL m^−2^ d^−1^, the faraday efficiency of 69.1% and the power utilization efficiency of 44.3% in the production process of H_2_, which was based on harvesting the wave energy of 0.5 Hz. Based on the construction of the high-output wave energy harvesting TENG, the instantaneous characteristic of the trigger switch was utilized to solve the limitation of TENG operating frequency [245]. In addition, the output short-circuit current of the whole TENG unit was greatly improved by combining the step-down upcurrent circuit and the hydrogen production rate of 64.5 μL min^−1^ was achieved by combining the hydrogen production device under the water wave driving condition. By using mathematical model to optimize the selection of catalyst and electrolyte and the operating temperature, the efficiency of the electrolytic water cell reached 97%. A simple and economical water electrolytic device was constructed by means of a membrane free electrochemical cell reactor. The self-powered electrolytic seawater hydrogen production device constructed by TENG combined with spring-assisted multilayer water wave energy harvesting obtained 0.020 μmol yield per cycle test (Fig. 18b) [246]. Moreover, a multilayer unit rotating wave energy harvesting TENG was installed with high triboelectric performance and low friction force through flexible contact of hair, and its average current density can reach 80 mA m^−2^. A water electrolysis electrode composed of MoS_2_/Ti_3_C_2_ composite material was prepared by utilizing the characteristic of two-dimensional transition metal carbides that can accelerate hydrogen absorption rate (Fig. 18c) [28]. By combining the wave energy harvesting TENG with a power management device with constant voltage function to drive the electrolytic water device constructed by the aforementioned motor, the entire system can obtain a hydrogen evolution rate of 7.1 mL min^−1^ at a working condition of 90 rpm. In the desalination research, a kind of ship-shaped hybrid water wave energy harvesting generator is proposed in combination with electroosmosis technology to realize self-powered desalination research. However, the high power consumption of electrodialysis greatly reduces the rationality of related design. Therefore, Ren et al. combined the designed high-output coaxial hybrid generator with a low-energy capacitive deion battery to design a self-powered desalination device (Fig. 18d) [247]. The practical test in the ocean further proves the huge application feasibility of the whole system. In terms of marine environmental protection research, a wave energy driven electrochemical CO_2_ reduction system was designed based on the spring-assisted multilayer TENG, which can use wave energy to convert CO_2_ into carbon-based liquid fuels that are easy to store and transport (Fig. 18e) [248]. The related principle is that CO_2_ is converted to formic acid under the addition of an electrochemical reaction. A conversion efficiency of close to 100% can be achieved through the optimized system. Through the construction of a prototype, it was found that this device can obtain 2.798 mmol formic acid per day by using 0.04 m^2^ of sea surface. Finally, the electric flocculation precipitation driven by low-frequency AC has better removal performance for some organic pollutants (Fig. 18f) [249]. The results show that under the same conditions, the removal rate of xylenol orange by low-frequency AC-driven electroflocculation is significantly superior to that by high frequency AC and DC. The related reason is that low-frequency AC power plants can enable Al-Al electrodes to have higher hydroxide production rates. At the same time, low-frequency AC driven electroflocculation can also be applied to the rapid separation of oil–water mixture. Therefore, inspired by the wave energy harvesting TENG with the low-frequency AC characteristics, a self-powered AC electric flocculation precipitation device based on wave energy harvesting TENG was proposed to achieve the rapid removal of marine organic pollutants and thus achieve the goal of marine environmental protection.

## Summary and Perspective

**Fig. 19:**
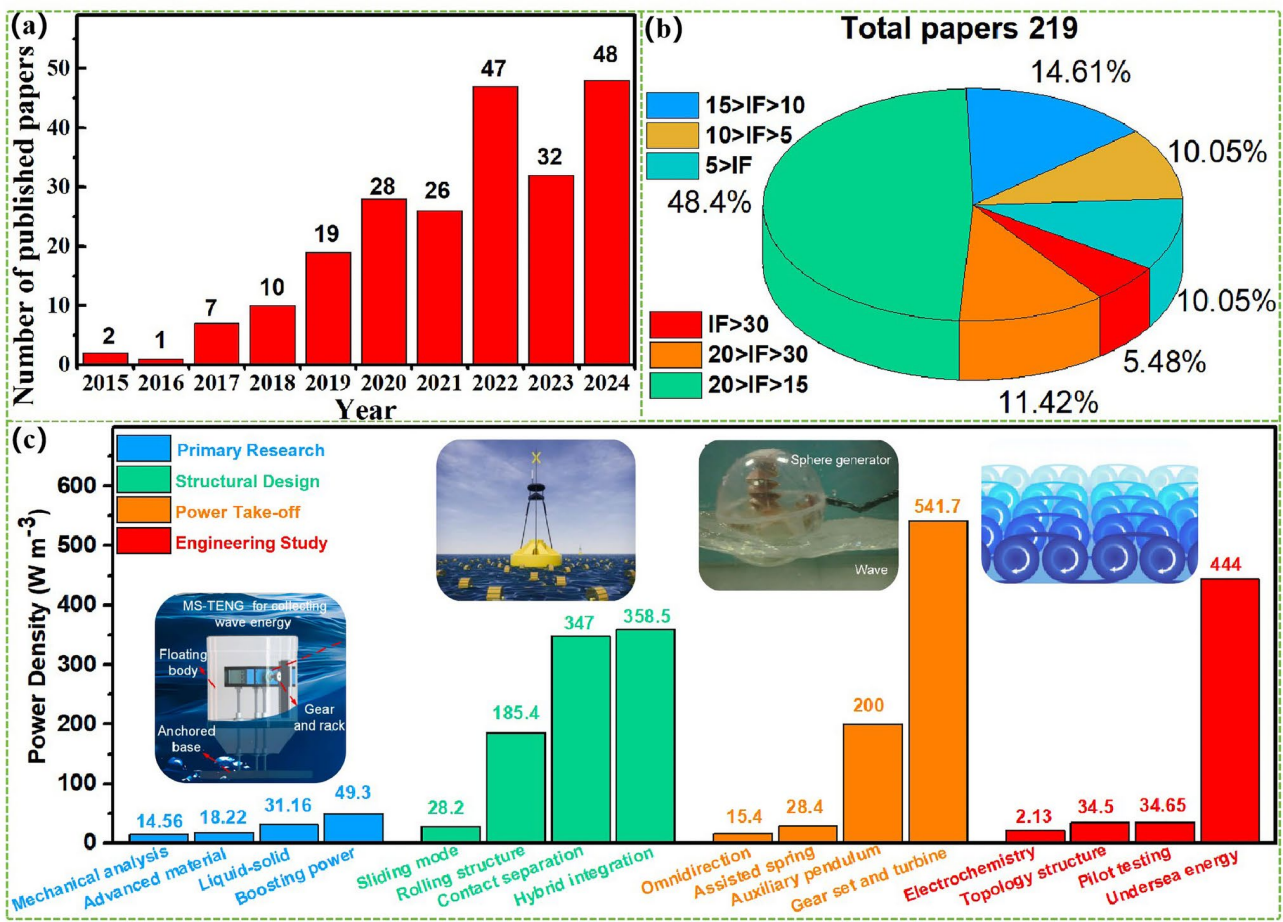
The statistical data analysis of research with the marine energy harvesting TENG. **a** Statistical chart of the number of papers published annually by marine energy harvesting TENG in the last ten years. **b** The data distribution map of marine energy harvesting TENG with the published papers in international journals at different levels. **c** Comparison of maximum power density of TENG for marine energy harvesting TENG in different research directions. Reproduced with permission from Ref. [72]. Copyright 2024, Elsevier. Reproduced with permission from Ref. [35]. Copyright 2020, Elsevier. Reproduced with permission from Ref. [209]. Copyright 2020, Elsevier. Reproduced with permission from Ref. [27]. Copyright 2023, The Royal Society of Chemistry

TENG, as an emerging technology that has only appeared for more than ten years, has ushered in a period of rapid development in the area of marine energy harvesting research in the past dozen years and has achieved many landmark scientific research achievements (Table 1). According to the relevant published data, it can be found that the number of scientific research papers in this research field has shown a rapid growth (Fig. 19a). About 80% of the 218 researches were published in international journals with an impact factor (IF) greater than 10 (Fig. 19b). This paper summarizes and discusses the key researching achievements of TENG in the area of marine energy development in recent years from three aspects: primary research, structural design and power take-off. In addition, the scale application of marine energy is the basic way to generate economic value, and the development of mature and commercialized marine energy harvesting TENG and self-powered electrochemical systems will be the ultimate development goal of this research field. Therefore, this paper also classifies and summarizes the important research work of marine renewable harvesting TENG in the aspect of engineering study. Relied on the relevant statistical data, it can be found that the marine energy harvesting TENG has achieved higher power density output in different research directions (Fig. 19c). Based on these numerous research achievements, we can fully think that the TENG technology has an important technical advantage in the fields of marine energy, undersea monitoring systems and blue hydrogen production based on self-powered electrochemistry. To further accelerate the rapid progress of TENG in the area of marine energy development research and help the new researchers to quickly find their own research topics, we summarized the existing problems in the 16 small research directions based on the above four major research directions and put forward reasonable prospects for future possible solutions.

**Table 1:** Summary of corresponding electrical output of various TENG devices for marine energy harvesting

Abbreviate	Classify	*Q*_*sc*_	*I*_*sc*_	Power	Power Density	Refs	Year
STVE-TENG	Liquid solid	/	880 μA	38.4 mW	1910 W m^−3^ (459 W m^−3^)	[21]	2022
CS-TENG	Topology design	110 nC	18.7 μA	7.07 mW	34.5 W m^−3^ (8.48 W m^−3^)	[22]	2023
MR-TENG	Electrochemistry	180 nC	4.6 μA	/	51.8 W m^−2^ (25.4 mW m^−2^)	[23]	2024
SD-TENG	Advanced material	0.01 μC	0.108 μA	31 mW	260 mW m^−2^	[24]	2020
UL-TENG	Mechanical analysis	/	20 μA	381 μW	1.9 W m^−3^	[25]	2024
LS TENG	Liquid solid	16.725 μC	290 μA	/	/	[26]	2018
CSS-TENG	Boosting power	4 µC	11.1 µA	29.1 mW	33 W m^−3^ (2.4 W m^−3^)	[27]	2024
RF-TENG	Rolling structure Omnidirection	24 nC	1.2 μA	10 mW	/	[28]	2015
HSO-TENGs	Contact separation	/	20 μA	210 μW	/	[29]	2024
CP-TENG	Sliding mode	286 nC	55 μA	22.1 mW	28.2 W m^−3^ (6.6 W m^−3^)	[30]	2020
TE-HGs	Hybrid integration	/	44.6 μA 1.78 mA	90.7 μW 79.6 μW	1.13 μW m^−2^ 2.25 μW m^−2^	[31]	2018
How-NG	Assisted spring	140 nC	4.65 mA	8.23 μW	/	[32]	2018
SSF-TENG	Auxiliary pendulum	286 μC	74 μA	16.3 mW	1.75 W m^−3^ (0.21 W m^−3^)	[33]	2021
DR-TENG	Gear set and turbine	/	36 μA	11 mW	/	[34]	2021
TS-TENG	Omnidirection	26.66 nC	0.79 μA	72.5 μW	0.21 W m^−2^	[35]	2019
S-TENGs	Pilot testing	2.22 μC	20.91 μA	61.2 mW	/	[36]	2022
PC-TENG	Topology design	25 nC	12 μA	0.18 μW	90 μW m^−2^	[37]	2020
BFM-TENG	Undersea energy	2.2 μC	265 μA	45 mW	444 W m^−3^	[38]	2020
RD‑TENG	Electrochemistry	/	1.6 mA	/	/	[39]	2020
SA-TENG	Advanced material Contact separation	162.1 nC	48.5 µA	5.38 mW	/	[49]	2017
SBSA-TENG	Advanced material	58 nC	22.3 μA	4.41 mW	2.4 W m^−3^	[50]	2018
BS-TENG	Advanced material Pilot test	66 nC	1.8 μA	1.3 mW	2.06 W m^−3^ (0.71W m^−3^)	[51]	2018
NMR-TENG	Advanced material	/	8.2 μA	35.4 μW	/	[52]	2021
OC TENGs	Advanced material	/	15 μA	2.88 μW	2.83 mW m^−2^	[45]	2020
FM-TENG	Advanced material	350 nC	6.5 µA	/	2 mW m^−2^	[53]	2020
E-TENG	Advanced material	7.67 μC	20.56 μA	1.278 mW	521 mW m^−2^	[54]	2024
FF-TENG	Advanced material	107 nC	15.5 µA	1 mW	/	[55]	2023
W-TENG	Advanced material	73 nC	36.1 µA	/	6.7 W m^−2^	[56]	2023
MW-TENG	Advanced material	/	/	/	14.7 W m^−3^ (2.8 W m^−2^)	[57]	2023
DMMs	Advanced material	87.82 nC	12.48 µA	0.35 mW	18.22 W m^−3^	[58]	2024
PS-TENG	Advanced material	5.9 nC	705 nA	/	57.3 mW m^−3^	[59]	2023
TENGs	Mechanical analysis	/	2.4 µA	8.03 mW	/	[60]	2021
WD-TENG	Mechanical analysis	19.81 nC	25.07 µA	/	1.3 W m^−2^	[63]	2022
RT-TENG	Mechanical analysis	/	30 µA	14.7 mW	/	[64]	2022
W-TENG	Mechanical analysis	0.87 μC	42 µA	20.1 mW	/	[65]	2022
MS-TENG	Mechanical analysis	692.96 μC	152.16 µA	6.47 W	14.56 W m^−3^	[67]	2024
SWTENG	Liquid–solid	/	25 µA	6.5 mW	/	[70]	2017
NI-TENG	Liquid–solid	2.1 μC	13.5 µA	1.03 mW	/	[71]	2018
U-TENG B- TENG	Liquid–solid	2.46 μC 2.34 μC	15.2 µA 1.25 µA	358.93 μW 463.51 μW	/	[72]	2019
TENGs	Liquid–solid	9.64 nC	4 mA	/	11.7 mW m^−2^	[73]	2021
WDSE-TENG	Liquid–solid	169 nC	74.67 µA	5.18 mW	79.18 mW m^−2^ (0.344 mW m^−2^)	[75]	2020
DB-TENG	Liquid–solid	30.7 nC	52 nA	/	/	[76]	2021
LS-TENG	Liquid–solid	96.9 nC	2.83 µA	/	/	[77]	2021
WT-TENG	Liquid–solid	3 μC	10 µA	/	13.1 mW m^−3^	[78]	2021
LST-TENG	Liquid–solid	62 nC	0.11 µA	215 μW	228 mW m^−3^	[79]	2022
C-TENG	Liquid–solid	/	23.4 nA	0.12 μW	/	[80]	2023
DE-TENG	Liquid–solid	4.57 μC	60 µA	4.05 mW	5.38 W m^−3^	[81]	2023
PT-TENG	Liquid–solid	/	900 µA	17.74 mW	36.16 W m^−3^	[82]	2024
SIBS	Boosting power	3 μC	/	/	13.2 mW m^−2^	[83]	2019
SM-TENG	Boosting power	μC	270 µA	12.2 mW	3.33 W m^−3^	[84]	2019
ID-TENG	Boosting power Electrochemistry	0.36 μC	30 µA	/	21.3 W m^−3^	[85]	2023
WF-TENG	Boosting power	227 μC	100 µA	6.5 mW	12.4 W m^−3^	[86]	2019
MS-TENG	Boosting power	/	180.9 µA	27.6 mW	15.18 W m^−3^ (3.56 W m^−3^)	[87]	2024
CEC-TENG	Boosting power	/	25.1 mA	25.8 mW	49.3 W m^−3^	[88]	2020
AFS-TENG	Boosting power	0.25 μC	23.3 mA	16.6 mW	/	[89]	2021
CEM-TENG	Boosting power	2.9 μC	15.09 mA	24.48 mW	11.4 W m^−3^	[90]	2022
MSCE-TENG	Boosting power	0.61 μC	50.5 µA	194.5 μW	27.8 W m^−3^	[91]	2023
VBE-TENG	Boosting power	1.1 μC	78 µA	34.3 mW	/	[92]	2023
CS-TENG	Boosting power	53 μC	1.3 mA	126.67mW	30.24 W m^−3^	[93]	2020
SC-TENG	Boosting power	864 nC	250 µA	8.3 mW	/	[94]	2022
TEE-TENG	Boosting power	2.2 μC	10.2 mA	25.9 mW	20.6 W m^−3^	[95]	2024
T-TENG	Rolling structure	220 nC	1.3 µA	/	10.6 W m^−3^	[96]	2019
HS-TENG	Rolling structure	157 nC	1.64 µA	544 μW	/	[97]	2019
MSN-TENG	Rolling structure	4.9 μC	5 µA	8.75 mW	8.69 W m^−3^	[98]	2019
S-TENG	Rolling structure	820 nC	15.5 µA	10.7 mW	20.57 W m^−3^	[99]	2021
NDM-FTENG	Rolling structure	125 nC	1.92 µA	/	4 W m^−3^	[100]	2021
BSA-TENG	Rolling structure	87 nC	0.29 µA	0.1 mW	/	[102	2022
CP-TENG	Rolling structure	46 μC	0.96 µA	/	0.91 W m^−3^	[102]	2022
SP-TENG	Rolling structure Omnidirection	430 nC	6.8 μA	/	13 W m^−3^ (2.24 W m^−3^)	[103]	2024
SR-TENG	Rolling structure	0.81 μC	8.3 µA	/	80.29 W m^−3^ (6.02 W m^−3^)	[104]	2023
TEWEH	Rolling structure Hybrid integration	88.36 nC 69.145 nC	1.34 µA 10.43 mA	/	13.77 W m^−3^ 148.24 W m^−3^	[105]	2023
MO-TENG	Rolling structure	379.06 nC	19.42 µA	37.17 mW	185.4 W m^−3^	[106]	2024
EC-TENG	Rolling structure	280 nC	22 µA	12 mW	/	[107]	2022
CE-TENG	Rolling structure	48 nC	30.82 µA	2.27 mW	7.75 W m^−3^	[108]	2023
SDR-TENG	Rolling structure	0.55 μC	84.4 µA	7.6 mW	16.8 W m^−3^	[109]	2024
SS-TENG	Rolling structure	500 nC	5µA	1.8 mW	/	[110]	2019
SB-TENG EMG	Rolling structure	/	/	0.5 mW 8.5 mW	10.1 W m^−3^	[111]	2023
RS-TENG	Rolling structure	/	/	455 nW	/	[112]	2021
WB-TENG	Contact separation	850 nC	147 µA	/	13.52 W m^−3^	[113]	2020
BITENG	Contact separation	/	10 µA	*/*	25 µW m^−2^	[114]	2019
OBL-TENG	Contact separation	26 μC	0.45 mA	38.7 mW	7.45 W m^−3^	[115]	2019
B-TENG	Contact separation	0.838 μC	75.35 µA	82.205mW	9.559 W m^−3^	[116]	2019
GA-TENG	Contact separation	/	/	3 mW	20.4 W m^−3^	[117]	2024
SMS-TENG	Contact separation Assisted spring	0.67 μC	120 µA	15.97 mW	15.2 W m^−3^	[118]	2018
BM-TENG	Contact separation Auxiliary pendulum Topology design	0.6 μC	60 µA	/	200 W m^−3^	[119]	2021
OME-TENG	Contact separation	900 nC	25 µA	10.4 mW	/	[120]	2024
SO-TENG	Contact separation Auxiliary pendulum	1.5 μC	138 µA	6.035 mW	2.6 W m^−2^	[121]	2024
ASM-TENG	Contact separation Topology design	0.9 μC	0.3 mA	45 mW	347 W m^−3^	[122]	2022
MH-TENG	Contact separation	2.9 μC	200.3 µA	16.2 mW	23.2 W m^−3^	[123]	2023
FH-TENG	Contact separation Assisted spring	7.9 μC	156.1 µA	10.9 mW	/	[125	2024
AR-TENG	Contact separation Omnidirection	0.7 μC	122 µA	12.3 mW	/	[125]	2021
SPS-TENG	Sliding mode	1.95 μC	10.73 µA	8.56 mW	14.71 W m^−3^	[126]	2019
MG-TENG	Sliding mode	26 μC	60 µA	54 µW	4.2 mW m^−2^	[127]	2019
MG-TENG	Sliding mode	3.25 μC	92 µA	3.2 mW	502 mW m^−2^	[128]	2022
CMLS-TENG	Sliding mode	130 nC	4.5 µA	/	730 mW m^−3^	[129]	2024
Arctic-TENG	Sliding mode	/	32 µA	14.3 mW	21.4 W m^−3^	[130]	2023
TD-TENG	Sliding mode	3.4 μC	120 µA	45.5 mW	7.3 W m^−3^	[131]	2019
WS-TENGs	Sliding mode	137.5 nC	29.89 µA	1.125 mW	1.578 W m^−3^	[132]	2023
SR-TENG	Sliding mode	/	3.35 µA	/	5.25 W m^−3^	[133]	2023
EING	Sliding mode	300 nC	40 µA	10.97 mW	8.79 W m^−3^	[134]	2021
MR-TENG	Sliding mode	130 nC	12 µA	2.52 mW	/	[135]	2020
RDR-TENG	Sliding mode	75 nC	1.5 µA	74 μW	/	[136]	2020
DS-TENG	Sliding mode	98.03 nC	3.63 µA	923.92 µW	/	[137]	2023
GS-TENG	Sliding mode	150 nC	3.2 µA	0.6 mW	0.28 W m^−3^	[138]	2022
ES- TENG	Sliding mode	27 nC	0.74 µA	28 µW	/	[139]	2021
T-EHG	Sliding mode	/	65.5 µA	71.59 mW	25.08 W m^−3^	[140]	2024
SB-NG	Hybrid integration	/	2 μA 13 mA	700 μW 6 mW	/	[141]	2020
SDEH	Hybrid integration	/	1.42 μA 1.12 mA 0.2 mA	0.25 mW 13.8 mW 1.58 mW	/	[142]	2022
SSTE-HNG	Hybrid integration	472 nC	26.56 μA 14 mA	/	17 W m^−3^ 9.8 W m^−3^	[143]	2022
MS-TENG	Hybrid integration	34 nC	21.5 μA	/	79 W m^−3^	[148]	2022
RSO-HNG	Hybrid integration	150 nC	3.7 μA 7.07 mA	0.5 mW 4 mW	/	[149]	2019
CPTE-NG	Hybrid integration	54 nC	3 μA 4 mA	15.21 μW 1.23 mW	/	[150]	2020
PT-HNG	Hybrid integration		35 μA 12 mA	5 mW 3.5 mW	/	[151]	2022
CP-CNG	Hybrid integration	~ 1.643 μC	140 μA 0.25 mA	16 mW 0.16 mW	/	[152]	2024
ETE-NG	Hybrid integration	/	100 nA 11 mA	85 nW 4mW	/	[153]	2020
FH-NG	Hybrid integration	~ 180 nC	~ 3.5 μA ~ 23 mA	5.2 mW 43.6 mW	2.02 W m^−3^ 16.96 W m^−3^	[154]	2022
H-WWEH	Hybrid integration	/	0.61 μA 6.4 mA	0.26 mW 6.2 mW	/	[155]	2021
SB-NG	Hybrid integration	135 nC	4.57 μA 11.9 mW	1.3 mW 3.5 mW	2.71 W m^−3^ 7.59 W m^−3^	[156]	2021
SF-TEG	Hybrid integration	~ 140 nC	1.832 μA ~ 15 mA	38 μW 120 mW	250 W m^−3^	[159]	2022
TLS-TEHG	Hybrid integration	/	0.2 μA 25 mA	8.8 μW 2.35 mW	/	[160]	2024
BCHNG	Hybrid integration	2.2 μC 11 μC	0.7 mA 7 mA 10.6 mA	39.2 mW 2.5 mW 30 mW	196 W m^−3^ 12.5 W m^−3^ 150 W m^−3^	[165]	2022
SPC-HEH	Hybrid integration	/	12.7 μA 14.3 mA 341 nA	9.025 mW 173.4 mW 4.84 μW	82.4 W m^−3^	[166]	2024
SPC-HEH	Hybrid integration	/	138.7 μA 8.5 mA 10.1 mA	9 mW 34.2 mW 26.2 mW	/	[168]	2024
MA-TENG	Assisted spring	/	~ 6 μA	/	~ 0.9. W m^−2^	[169]	2017
HW-NG	Assisted spring Hybrid Integration	/	28 μA 1.5 mA	1.72 mW 1.48 mW	0.4 W m^−2^ 0.3 W m^−2^	[170]	2021
SSR-TENG	Assisted spring	45 nC	10 μA	0.14 mW	/	[171]	2023
CFP-TENG	Assisted spring	757.4 nC	17.43 μA	3.12 mW	1.5 W m^−2^	[172]	2023
ML-HNG	Assisted spring	420 nC	135 μA	7.1 mW	28.4 W m^−3^	[173]	2024
NS-TENG	Auxiliary pendulum	76 nC	29.7 μA	9.1 mW	/	[175]	2021
SSEP-TENG	Auxiliary pendulum	~ 48 μC	12 μA	/	/	[176]	2024
SLEP-TENG	Auxiliary pendulum	90.9 nC	16.9 μA	7.2 mW	10.26 W m^−3^	[177]	2024
SS-TENG	Auxiliary pendulum	/	53 μA	13.7 mW	/	[178]	2022
MFA-TENG	Auxiliary pendulum	255 nC	18 μA	1.34 mW	/	[179]	2024
SS-TENG	Auxiliary pendulum	195 nC	6.3 μA	456 mW (0.48 mW)	1.29 W m^−3^ (0.14 W m^−3^)	[180]	2020
C-TENG	Auxiliary pendulum	35–38 nC	1.33 μA	159 μW (32 μW)	231.6 mW m^−3^ (39.8 mW m^−3^)	[181]	2020
CP-TENG	Auxiliary pendulum	150 nC	13.4 μA	11.33 mW (3.6 mW)	15.4 W m^−3^ (4.9 W m^−3^)	[182]	2022
SO-TENG	Auxiliary pendulum	0.75 μC	33 μA	2.76 mW	2.62 W m^−2^	[183]	2024
MBC-TENG	Gear set and turbine	576 nC	75 μA	48 mW 31 mW	15.5 mW m^−3^ 10 mW m^−3^	[184]	2021
MC-TENG	Gear set and turbine	389.9 nC	85.4 μA	43.5 mW 8.19 mW	38.46 W m^−3^ 7.24 W m^−3^	[185]	2024
WLM-TENG	Gear set and turbine	1.1 μC	30 μA	50 mW 113 μW	14.1 W m^−3^ 32 W m^−3^	[186]	2022
ES-TENG	Gear set and turbine	110 nC	19 μA	6.2 mW	/	[187]	2023
ER-TENG	Gear set and turbine	392 nC	11.2 μA	9.8 mW (5.54 mW)	(15.67 W m^−3^)	[188]	2024
SR-TRNG	Gear set and turbine	0.3 μC	70 μA	/	45.18 mW kg^−1^	[189]	2023
BSR-HNG	Gear set and turbine Hybrid integration	40 nC	4 μA 120 mA	0.4 mW 0.12 W	~ 90 W m^−3^	[190]	2024
MT-TENG	Gear set and turbine	~ 0.55 μC	55 μA	21.40 mW	30.62 W m^−3^	[191]	2024
CO-TENG	Gear set and turbine	190 nC	6 μA	/	/	[192]	2021
MIWEH	Gear set and turbine Hybrid integration	/	/	173.5 μW 99.7 mW	/	[193]	2023
DSM-TENG	Gear set and turbine	/	1.24 mA	470 mW 130 mW	/	[194]	2024
OWC-TENG	Gear set and turbine	141.42 nC	55.45 μA	5.28 mW	114.8 W m^−3^	[195]	2023
TEH-NG	Gear set and turbine Hybrid integration	184.5 nC 809.2 μC	22.4 μA 142.5 mA	(3.41mW) (40 mW)	(141.7 W m^−3^) (400 W m^−3^)	[196]	2024
RHTEWEH	Gear set and turbine	~ 0.87 μC	122 μA 80 mA	115 mW 350 mW	32.55 W m^−3^ 329.78 W m^−3^	[197]	2024
TENG-NW	Omnidirection	/	0.93 mA	~ 37 mW	2.6 W m^−2^	[198]	2015
WS-TENG	Omnidirection	~ 1.8 μC	150 μA	/	/	[199]	2016
OS-TENG	Omnidirection	270 nC	76 μA	475 μW	/	[200]	2019
U-TENG	Omnidirection	38 nC	2.07 μA	99.8 μW	218.2 mW m^−3^	[201]	2023
FS-TENG	Omnidirection	~ 300 nC	~ 17 μA	3.31 mW	8.38 W m^−3^	[203]	2023
S-TENG	Omnidirection	1 μC	120 μA	8.5 mW	4.84 W m^−3^	[204]	2020
e-TENG	Omnidirection	/	110 μA	11.2 mW	670 W m^−3^	[205]	2020
FL-TENG	Omnidirection	761 nC	51 μA	/	15.4 W m^−3^	[206]	2022
O-TENG	Omnidirection	722 nC	72 μA	10 mW (0.273 mW)	/	[207]	2022
Se-TENG	Omnidirection	/	0.41 μA	/	0.22 mW m^−2^	[208]	2022
FDS-TENG	Omnidirection	680 nC	26 μA	/	8.3 W m^−3^ 1.1 W m^−3^	[209]	2024
DLS-TENG	Omnidirection	1.263 μC	4.56 μA	742.96 μW	~ 3.55 W m^−3^	[210]	2024
i-TENG	Omnidirection	/	2.03 μA	101.5 µW	7.25 W m^−3^	[211]	2023
S-TENG	Pilot testing	0.7 μC	11.94 μA	25.22 mW	34.65 W m^−3^	[212]	2021
HM-TENG	Pilot testing	67.2 μC	18 μA	1.13 mW	2.44 W m^−3^	[213]	2022
OM-TENG	Pilot testing	60.82 μC	444 μA	20.8 mW	28.9 W m^−3^	[214]	2024
SC-TENG	Pilot testing	2.5 μC	2 mA	3 mW	65 mW m^−2^	[215]	2024
CH-EH	Pilot testing Hybrid integration	/	4 mA	/	/	[216]	2024
AMS-TENG	Topology design	15 µC	187 µA	10 mW	13.23 W m^−2^	[217]	2017
DS-TENG	Topology design	47 µC m^−2^	8 μA	/	0.7 W m^−2^	[218]	2018
BJ-TENG	Topology design	6 nC	2.3 μA	/	/	[219]	2017
3D-TENG	Topology design	3.7 μC	27.77 μA	3.14 mW	11.7 W m^−3^	[221]	2023
UWE-TENG	Undersea energy	/	1.43 mA	/	3.62 kW m^−2^	[222]	2017
UF-TENG	Undersea energy	/	1.43 μA	9.1 μW	/	[223]	2021
S-TENG	Undersea energy	43.2 nC	8.7 μA	79.023 μW	/	[224]	2021
P-TENG	Undersea energy	18.5 nC	7.2 μA	193.4 μW 27.6 μW	0.3357 W m^−2^	[225]	2022
CSM-TENG	Undersea energy	5–20 μC m^−2^	/	11.6 μW	5.56 mW m^−2^	[226]	2021
TE-SFT	Undersea energy	0.17 μC	~ 3.18 μA	/	5.56 W m^−3^	[227]	2023
STB-TENG	Undersea energy	75 nC	17.5 μA	0.74 mW	/	[228]	2022
SBF-TENG	Undersea energy	1.23 μC m^−2^	~ 80 μA	0.668 μW	1.67 mW m^−2^	[229]	2023
BCHNG	Undersea energy	0.34 μC	4 μA	0.69 mW	/	[230]	2022
UTENG	Undersea energy	/	~ 0.8 mA	200 mW	5.11 W m^−3^	[231]	2022
TEHN	Undersea energy Hybrid integration	270.46 nC	~ 15 μA 8.19 mA	449.74 mW	/	[232]	2023
MSM-TENG	Electrochemistry	70 nC	0.191 mA	13.6 mW	/	[236]	2022
PTS-TENG	Electrochemistry	16.6 μC m^−2^	16.1 μA	/	31 mW m^−2^	[237]	2022
MSLT	Electrochemistry	/	2.4 μA		16.75 W m^−3^	[239]	2022
SCC-TENG	Electrochemistry	302 nC	27 μA	12.6 mW	/	[240]	2022

(i) The values of the table in brackets represent average power or average power density.

(ii) For the hybrid generator, the first item represents the TENG output parameter, the second item represents the EMG output parameter, and the third item represents the PENG output parameter

### Primary Research

As a source of technological development, primary research fundamentally determines the ultimate development height and application breadth of this technology. In order to better empower the TENG technology development of marine energy, the current relevant research has the following problems. At the same time, the corresponding solution was put forward soon after.

- *Advanced materials*: At present, high-output triboelectric materials are mainly focused on positive triboelectric materials, while research on negative frictional electric materials. In future, the corresponding research should develop new materials with ultra-high negative triboelectric properties through functional group design through theoretical model research and other methods. In addition, more in-depth study on the triboelectric mechanism of different materials to reveal the corresponding mechanism will be an vital researching direction for the design of positive and negative triboelectric materials to greatly boost the electrical output of TENG. Finally, for the demand of TENG research with environmental protection characteristics, the construction of degradable highly conductive electrodes by graphene will be an important way [250].
- *Mechanical analysis*: The most of the relevant studies are theoretical studies that do not fully combine the corresponding experimental verification and the coupling mechanism between multiple factors is still unclear. It is very necessary to conduct further systematic research on all factors affecting the TENG performance of marine energy harvesting through theoretical and experimental methods. In addition, through simulation software and related research on wave equation, there is a serious lack of innovative research, and the guiding significance of relevant research conclusions is very limited. In the subsequent research, we should make full use of such methods for rapid data analysis combined with artificial intelligence technology to break through the current limitations based on traditional models and develop a new TENG structure model with better performance.
- *Liquid–solid TENG*: Since the slow ion adsorption occurring in the double electric layer largely weakens the electrical output and stability of the liquid–solid TENG, it is a significant studying direction for the practical application of liquid–solid TENG to further study the mechanism of liquid–solid triboelectricity as well as explore the material structure design that can minimize the ion adsorption effect on this basis and then develop the corresponding functional materials combined with the corresponding triboelectric material preparation process [251]. As the main body of the ocean, seawater is the most important raw material for the installation of liquid–solid TENG. Therefore, it will have significant significance to accelerate the research in this direction in the subsequent research.
- *Exciting charge*: In the face of the ultra-low-frequency characteristics of marine energy, it is difficult for the charge-excited TENG to obtain sufficient excitation charge under the drive of water wave so that the corresponding technology can fully exert its own advantages. It is an important research point to improve the practicability of charge excitation technology in marine energy harvesting process by studying system composition optimization and circuit management. At the same time, limited by the breakdown of air and dielectric materials, the upper limit of the electrical output of charge-excited TENG is still necessary to be further improved. Developing dielectric materials with higher breakdown field strength and more efficient charge excitation circuits will be the focus of further research.

### Structural Design

The structural design has a great influence on the various properties of the TENG, which in turn has an important impact on the harvesting of marine energy. At present, four kinds of TENG structural design have their own characteristics, but also have corresponding limitations. The development of avant-garde structures with more excellent performance will be an important driving force for the continuous innovation and development of this research field.

- *Rolling structures*: The rolling TENG has been extensively studied for its simplest mounting method and excellent wear resistance. Due to the low electrical output caused by the small contact area, the rolling structure TENG has undergone the development process of single-point contact to multipoint contact, multiline contact mode transition, and multisurface contact. However, the output performance of the rolling ball TENG is still relatively low. Therefore, the related development direction of rolling TENG in future should mainly focus on making full use of its excellent stability due to the development of highly stable charge sources and then combined with air variable capacitance to develop the corresponding high-output marine energy harvesting TENG.
- *Contact separation*: This structure design has problems such as large energy loss caused by inelastic collision and low output frequency, and it is difficult to achieve stable high electrical output. Meanwhile, the multilayer structure integration method is impossible to realize the high-density integration of TENG units due to the problem of backward charging of TENG units caused by asynchronous phase. More importantly, the high electrical output of the contact separation TENG based on the high charge density will generate high electrostatic attraction, which makes the separation process of the TENG more difficult. It is urgent to reduce the energy loss caused by inelastic collision of the contact separation TENG, synchronize the output phase of each unit of the multilayered TENG and optimize the separation process through mechanical research methods.
- *Sliding modes*: The marine energy harvesting TENG often needs to achieve the corresponding mechanical energy harvesting through inertial force capture, which is difficult to provide a large torque to drive sliding mode TENG due to sliding friction. In addition, the higher friction will seriously affect the service life of the TENG. Although the research of sliding TENG in marine energy harvesting can be improved by soft contact, adding lubricants and gap sliding design, these designs were achieved at the expense of the corresponding high electrical output. Thus, by developing triboelectric materials with high electrostatic induction and the air variable capacitors to build a method that relies entirely on electrostatic induction to obtain a high-output TENG will be the ultimate choice.
- *Hybrid integration*: Since the existing marine energy infrastructures are basically designed to full play to the advantages of electromagnetic generators with the high efficiency in high frequency, which have problems large volume, complex system, and low efficiency ratio. Therefore, when the TENGs are integrated with existing marine energy infrastructures, it will face the advantages of not being able to fully utilize the characteristics of high efficiency in low frequency and the lightweight in constructing a floating marine energy harvesting devices. In order to effectively solve the above challenges, it will be an important research direction to fully consider the characteristics of different types of generator in subsequent relevant studies, and make full play to their respective advantages through integrated system optimization design to achieve optimal marine energy harvesting efficiency and promote the industrialization of TENG. Furthermore, the development of a more efficient hybrid generator management module based on the current corresponding power management circuit design will have the dual role of improving the electrical energy output of TENG devices and promoting its application research.

### Power Take-Off

In the process of marine energy harvesting, power take-off captures the power contained in the movement of marine fluctuation and drives the TENG work to realize the electromechanical conversion. Current research on power take-off based on various mechanical structures has greatly improved the corresponding energy harvesting and conversion efficiency. However, the design of more advanced power take-offs and how to maximize its function are still areas that need to be studied in depth.

- *Assisted spring*: The design of the integrated spring structure not can increase the output frequency of the TENG through damping effect, but can effectively reduce the driving force of the TENG to realize the harvesting of ripple energy. However, the mechanical impedance changes caused by its introduction may reduce the electrical output of the entire device through the working phase. In addition, the integrated spring design of the TENG still faces an important problem, which is difficult to make the TENG produce UHF output to achieve high electrical power. How to design the TENG with higher and more stable electrical output through the spring is still an important research direction.
- *Auxiliary pendulum*: The pendulum power take-off has higher energy storage characteristics and more efficient damping characteristics than the spring. In addition, the low center of gravity attribute, on the one hand, gives the whole TENG device higher mobility to further increase the corresponding power capture, on the other hand, helps to reduce the center of gravity of the entire TENG device to improve its stability and safety in complex marine environments. These advantages make pendulum TENG more widely studied. However, pendulum damping has a fatal flaw, its high swing period is very bad for efficient electromechanical conversion. Therefore, it is very important for the utilization of tilting TENG to improve the utilization efficiency of damping effect in wave period as much as possible by structural design and matching optimization of TENG parameters.
- *Gear set and turbine*: The current research of gear group in marine energy harvesting TENG only uses a simple principle to play an auxiliary role, and does not give full play to its excellent mechanical adjustment characteristics through integrated and systematic design. Integrated design of power take-off, gear set and TENG through highly integrated design has an important influence on improving the stability and electrical output of the entire device system. Turbine, as a traditional shore-based power take-off, is only used as a pneumatic device in the current research of TENG and the install TENG devices have the characteristics of huge reminder. In future, related research should develop hybrid energy harvesting devices based on the lightweight characteristics of TENG and the design requirements of turbine for lightweight characteristics.
- *Omnidirectional design*: The TENG designed with the inner and outer ring structure can maintain the optimal working state in any direction, but the low electrical output is common during the relevant period reported by the relevant research work. Therefore, based on the previous relevant research, the basic model of the TENG for omnidirectional marine energy harvesting is constructed through the omnidirectional freedom design of the energy harvester and power take-off as well as the omnidirectional working mode of the TENG. On this basis, optimizing the coordination among the above three system components combined with charge excitation and other methods to significantly improve the corresponding TENG electrical output will be the basic research idea for subsequent development.

### Engineering Study

As the ultimate goal of related technology development, engineering application is the most critical factor to determine the sustainable development of related research fields. Although the relevant engineering research has made some progress at present, TENG technology has not been applied in the area of marine energy harvesting, which comes from the limitation that some indicators have not reached the commercial standard. It is an important way to realize the benign and harmonious development of TENG technology by adopting the mutually promoting development mode of research and application.

- *Pilot testing*: It is found that the wave parameters have vital influence on the electrical output of the one-meter TENG device in the large wave pond and ocean wave test. Therefore, subsequent relevant studies should focus on the hydrological parameters of the TENG installation area, and on this basis, it is necessary to determine the size parameters and internal structural design of the TENG device. In addition, in order to simplify the subsequent engineering design process, the corresponding physical model and technical standard are constructed and optimized through the corresponding pilot test, thus providing universal theoretical basis for the design of TENG in different sea areas, which plays an important cornerstone role in the entire engineering research. Finally, the relevant design of the pilot TENG should be tested according to relevant technical and commercial standards with the deep participation of relevant enterprises.
- *Topological structure*: The most research of topological structure only stay in the theoretical research and simple proof of principle, and do not carry out a very systematic scale test. In addition, topological structure research has not provided any corresponding evaluation criteria so far, and constructing reasonable and accurate evaluation according to relevant needs will be one of the urgent problems to be solved in future. More importantly, the complexity of the marine environment often brings many adverse effects and challenges to the application of all marine equipment. Therefore, the research on the topology of TENG should be implemented step by step according to the requirements of theoretical research and engineering feasibility assessment.
- *Undersea energy*: At present, the undersea energy harvesting TENG is the earliest and most promising research direction to enter commercial application testing. However, the current relevant research has not fully considered the relevant requirements of undersea monitoring equipment for undersea energy harvesting, but is only in a stage of explosive exploration and research. Therefore, in the next few years, the undersea energy harvesting TENG should fully take the structural characteristics of the current undersea exploration robot and the surrounding environment of the undersea fixed sensor device to carry out relevant research work on the basis of ensuring the function of related equipment.
- *Electrochemical application*: In the self-powered electrochemical application research, many researches are carried out by combining the existing electrochemical technology with the existing TENG technology, which fails to give full play to the best technical performance of the relevant electrochemical technology. Therefore, the subsequent relevant researches should be based on the output characteristics of TENG harvested by marine energy, and targeted development of corresponding electrochemical technology to achieve efficient energy utilization and high-speed target material production. These studies are questions that the field will have to address in the coming years. In addition, it is also an momentous researching direction to continue to develop low-power and high-value seawater resource separation technologies based on electrochemical technology and then accelerate the rapid development of the entire industry with higher economic benefits.

To sum up, although TENG still faces some challenges in the development of commercial applications for marine energy harvesting, based on the numerous milestone research achievements as well as the issues and prospects raised above, we firmly believe that the rapid development of TENG technology for harvesting marine energy will enable the blue energy dream of all mankind to be realized as scheduled in the near future.
